# Supplementary material for: The Chemical Space of Marine Antibacterials: Diphenyl Ethers, Benzophenones, Xanthones, and Anthraquinones
Source: Molecules. 2023 May 13;28(10):4073. doi: 10.3390/molecules28104073 (PMC10221046; doi:10.3390/molecules28104073)
Supplement: Supplementary file 1 [file molecules-28-04073-s001.zip › molecules-2349831-supplementary.pdf]

Supplementary Materials

The Chemical Space of Marine Antibacterials: Diphenyl Ethers, Benzophenones, Xanthones, and Anthraquinones

José X. Soares [1,2,3], Inês Afonso [1], Adaleta Omerbasic [1], Daniela R. P. Loureiro [1,2,3], Madalena M. M. Pinto [1,2] and Carlos M. M. Afonso [1,2,*]

[1] Laboratory of Organic and Pharmaceutical Chemistry, Department of Chemical Sciences, Faculty of Pharmacy, University of Porto, Rua de Jorge Viterbo Ferreira, 228, 4050-313 Porto, Portugal; jfxsoares@ff.up.pt (J.X.S.); dloureiro@ff.up.pt (D.R.P.L.); madalena@ff.up.pt (M.M.M.P.)

[2] Interdisciplinary Center of Marine and Environmental Investigation (CIIMAR/CIMAR), Edifício do Terminal de Cruzeiros do Porto de Leixões, Av. General Norton de Matos s/n, 4050-208 Matosinhos, Portugal

[3] LAQV-REQUIMTE, Department of Chemical Sciences, Faculty of Pharmacy, University of Porto, Rua de Jorge Viterbo Ferreira, 228, 4050-313 Porto, Portugal

* Correspondence: cafonso@ff.up.pt

Table of Tables

Table S1. Anti-infective Marine polyketides isolated from marine sources....................3
Table S2. Loading plots obtained using different scaling techniques............................84

Table of Figures

Figure S1. Heatmap of the distribution of the scaled dataset using different scaling techniques: a) without scaling; b) StandardScaler; (c) QuantileTransformer; (d) RobustScaler; (e) Normalizer; (f) MaxAbsScaler; (g) MinMaxScaler; (h) PowerTransformer. 96

Figure S2. Loading plots obtained using different scaling techniques: a) without scaling; b) StandardScaler; (c) QuantileTransformer; (d) RobustScaler; (e) Normalizer; (f) MaxAbsScaler; (g) MinMaxScaler; (h) PowerTransformer. 97

Figure S3. Number of unique fingerprints using different bits lengths. 98

Figure S4. Number of clusters and the number of compounds per cluster using different threshold values. 99

Figure S5. Distribution of log DD values according to the MP scaffolds (a) and clusters (b). 100

Figure S6. TMAP visualization of the studied MPs colored according to the scaffold. 101

Table S1. Anti-infective Marine polyketides isolated from marine sources.

ID	Name and structure	Activity	Source	Ref.
AQ001	6,6′-oxybis(1,3,8-trihydroxy-2-((S)-1-methoxyhexyl)anthracene-9,10-dione) 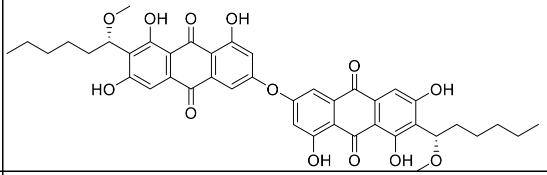	*S. aureus* (zone of inhibition 14 mm)	*Aspergillus versicolor* isolated from a marine clam collected at the East China Sea, China.	[1]
AQ002	6,6′-oxybis(1,3,8-trihydroxy-2-((S)-1-hydroxyhexyl) anthracene-9,10-dione) 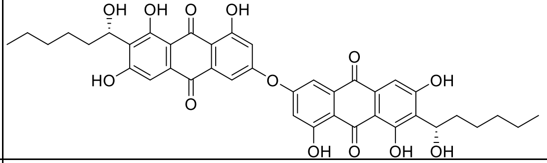	*S. aureus* (zone of inhibition 19 mm)		
AQ003	1′-*O*-methylaverantin 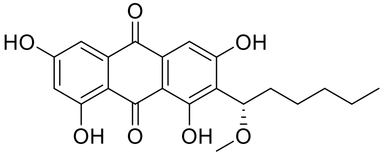	*S. aureus* (zone of inhibition 0 mm)<br>*B.subtilis* (MIC > 25 μM)<br>*P. aeruginosa* (MIC > 25 μM)<br>*E. coli* (MIC > 25 μM)<br>*S. aureus* (MIC > 25 μM)	*Aspergillus versicolor* HBU-2017-7 collected at the Bohai Sea.	[1,2]
AQ004	Averantin 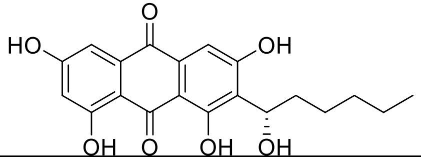	*S. aureus* (zone of inhibition 0 mm)	*Aspergillus versicolor* isolated from a marine clam collected at the East China Sea, China.	[1]

AQ005	Averythrin 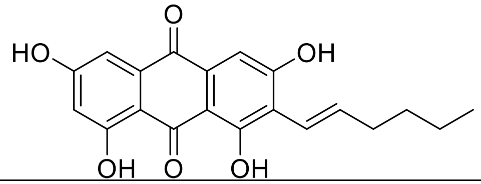	*S. aureus* (zone of inhibition 0 mm)		
AQ006	3,8-dihydroxy-1-propylanthraquinone-2-carboxylic acid 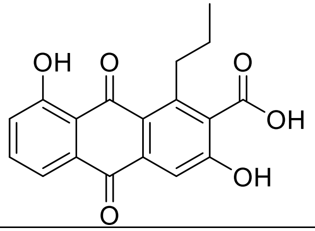	*S. viridochromogenes* (zone of inhibition 12 mm)<br>*S. aureus* (zone of inhibition 14 mm)	*Streptomyces sp.* B8000 isolated from a marine sediment collected at the Laguna de Terminos, Gulf of Mexico.	[3]
AQ007	8-Acetoxy-3-hydroxy-1-propylanthraquinone-2-carboxylic acid 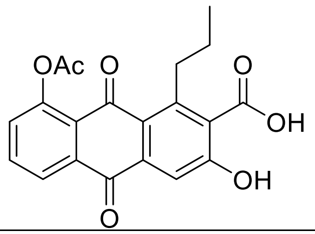	*S. viridochromogenes* (zone of inhibition 0 mm)<br>*S. aureus* (zone of inhibition 0 mm)	Semi-synthetic compound obtained from AQ006.	
AQ008	8-Acetoxy-3-methoxy-1-propylanthraquinone-2-carboxylic acid methyl ester	*S. viridochromogenes* (zone of inhibition 0 mm)<br>*S. aureus* (zone of inhibition 0 mm)	Semi-synthetic compound obtained from AQ006.	

	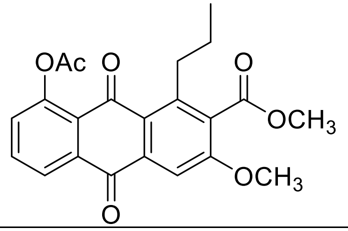			
AQ009	8-hydroxy-3-methoxy-1-propylanthraquinone 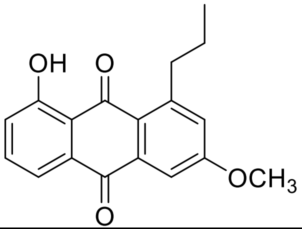	*S. viridochromogenes* (zone of inhibition 12 mm) *S. aureus* (zone of inhibition 14 mm)	*Streptomyces sp.* B8000 isolated from a marine sediment collected at the Laguna de Terminos, Gulf of Mexico.	
AQ010	8-acetoxy-3-methoxy-1-propylanthraquinone 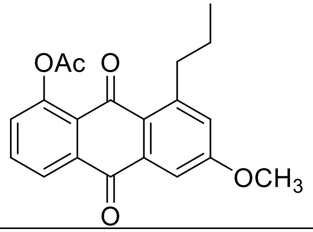	*S. viridochromogenes* (zone of inhibition 0 mm) *S. aureus* (zone of inhibition 0 mm)	Semi-synthetic compound obtained from AQ009.	
AQ011	3,8-dihydroxy-1-propylanthraquinone 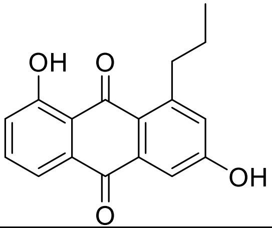	*S. viridochromogenes* (zone of inhibition 0 mm) *S. aureus* (zone of inhibition 0 mm)	*Streptomyces sp.* B8000 isolated from a marine sediment collected at the Laguna de Terminos, Gulf of Mexico.	

AQ012	3,8-dimethoxy-1-propylanthraquinone<br>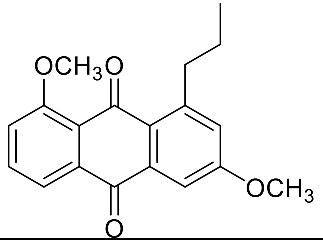 	*S. viridochromogenes* (zone of inhibition 0 mm)<br>*S. aureus* (zone of inhibition 0 mm)	Semi-synthetic compound obtained from AQ011.	
AQ014	Auxarthrol D<br>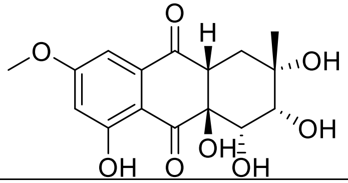 	*B. subtilis* (MIC = 100 μM)<br>*E. coli* (MIC = 100 μM)<br>*Proteus sp.* (MIC = 50 μM)<br>*V. parahaemolyticus* (MIC = 50 μM)<br>*P. aeruginosa* (MIC = 50 μM)<br>*M. phlei* (MIC = 25 μM)		
AQ015	Auxarthrol E<br>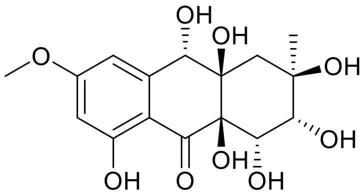 	*B. subtilis* (MIC > 200 μM)<br>*E. coli* (MIC > 200 μM)<br>*Proteus sp.* (MIC > 200 μM)<br>*V. parahaemolyticus* (MIC > 200 μM)<br>*P. aeruginosa* (MIC > 200 μM)<br>*M. phlei* (MIC > 200 μM)	*Sporendonema casei* HDN16-802 isolated from a marine sediment collected in Zhangzi Island, Dalian, Liaoning Province, China.	[4]
AQ016	Auxarthrol F<br>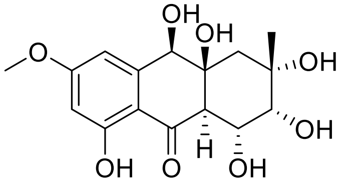 	*B. subtilis* (MIC = 200 μM)<br>*Proteus sp.* (MIC = 200 μM)<br>*P. aeruginosa* (MIC = 200 μM)<br>*M. phlei* (MIC = 200 μM)<br>*E. coli* (MIC > 200 μM)<br>*V. parahaemolyticus* (MIC > 200 μM)		
AQ017	Auxarthrol G	*V. parahaemolyticus* (MIC = 100 μM)		

	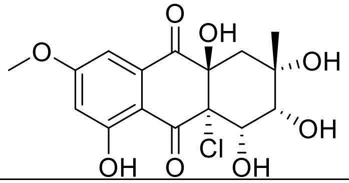	*P. aeruginosa* (MIC = 100 μM)<br>*M. phlei* (MIC = 50 μM)<br>*B. subtilis* (MIC = 25 μM)<br>*Proteus sp.* (MIC = 25 μM)<br>*E. coli* (MIC > 200 μM)		
AQ018	Auxarthrol H<br>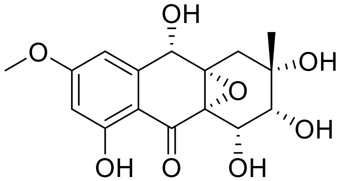	*B. subtilis* (MIC > 200 μM)<br>*E. coli* (MIC > 200 μM)<br>*Proteus sp.* (MIC > 200 μM)<br>*V. parahaemolyticus* (MIC > 200 μM)<br>*P. aeruginosa* (MIC > 200 μM)<br>*M. phlei* (MIC > 200 μM)		
AQ019	4-dehydroxyaltersolanol A<br>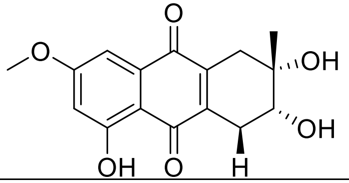	*Proteus sp.* (MIC = 50 μM)<br>*M. phlei* (MIC = 25 μM)<br>*B.subtilis* (MIC = 25 μM)<br>*V.parahaemolyticus* (MIC = 25 μM)<br>*P. aeruginosa* (MIC = 25 μM)<br>*E. coli* (MIC > 200 μM)		
AQ020	Altersolanol B<br>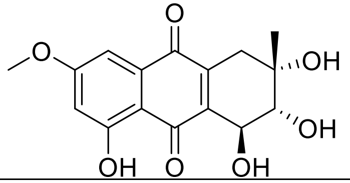	*Proteus sp.* (MIC = 100 μM)<br>*M. phlei* (MIC = 25 μM)<br>*B. subtilis* (MIC = 25 μM)<br>*V.parahaemolyticus* (MIC = 25 μM)<br>*P. aeruginosa* (MIC = 12.5 μM)<br>*E. coli* (MIC > 200 μM)		
AQ021	Aloesaponarin II	*B. subtilis* (ATCC 6633) (MIC) (zone of inhibition 22 mm)<br>*E. coli* (zone of inhibition 21 mm)<br>*P. aeruginosa* (zone of inhibition 21 mm)	*Streptomycetes* M097 collected in Jiaozhou Bay, China.	[5,6]

	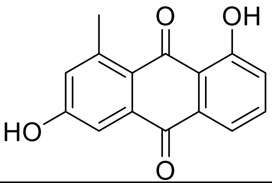	*S. aureus* (ATCC 25923) (zone of inhibition 18 mm)		
AQ022	Chrysophanol 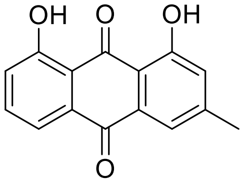	*V. parahaemolyticus* (ATCC 17802) (MIC = 25 μM)<br>*V. anguillarum* (ATCC 19019) (MIC > 50 μM)<br>*P. putida* (ATCC 17848) (MIC > 50 μM)<br>*E. coli* (ATCC 35218) (zone of inhibition 0 mm)<br>*B. subtilis* (ATCC 6633) (zone of inhibition 10 mm)<br>*P. aeruginosa* (ATCC 27853) (zone of inhibition 12 mm)<br>*S. aureus* (ATCC 25923) (zone of inhibition 10 mm)	*Trichoderma* sp. isolated from a marine clam collected in the Yellow Sea, Chengshantou Island, Weihai City, China.	[6,7]
AQ023	6,8-di-*O*-methylaverantin 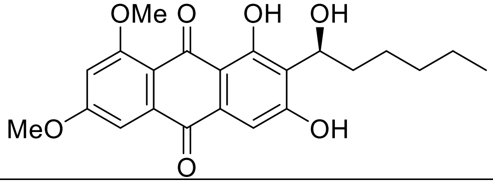	*E. coli* (zone of inhibition 7 mm)<br>*S. aureus* (zone of inhibition 0 mm)	*Aspergillus versicolor* EN-7 isolated from a marine alga *Sargassum thunbergii* collected in Qingdao coastline of Shandong Province, China.	[8]
AQ024	6,8-di-*O*-methylversiconol	*E. coli* (zone of inhibition 6.5 mm)<br>*S. aureus* (zone of inhibition 0 mm)		

	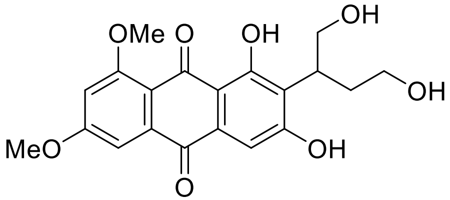			
AQ025	6,8-di-*O*-methylaverufin<br>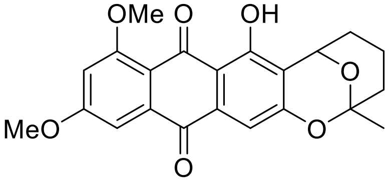	*E. coli* (zone of inhibition 0 mm)<br>*S. aureus* (zone of inhibition 0 mm)<br>*B. subtilis* (MIC > 25 μM)<br>*P. aeruginosa* (MIC > 25 μM)<br>*E. coli* (MIC > 25 μM)<br>*S. aureus* (MIC > 25 μM)	*Aspergillus versicolor* EN-7 isolated from a marine alga *Sargassum thunbergii* collected in Qingdao coastline of Shandong Province, China.	[8],[2]
AQ026	6-*O*-methylaverufin<br>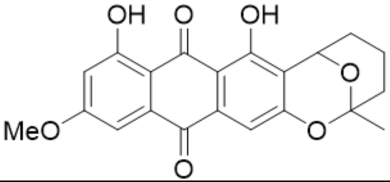	*E. coli* (zone of inhibition 0 mm)<br>*S. aureus* (zone of inhibition 0 mm)	*Aspergillus versicolor* EN-7 isolated from a marine alga *Sargassum thunbergii* collected in Qingdao coastline of Shandong Province, China.	[8]
AQ027	6,8-di-*O*-methylnidurufin	*E. coli* (zone of inhibition 6.5 mm)<br>*S. aureus* (zone of inhibition 7 mm)		

	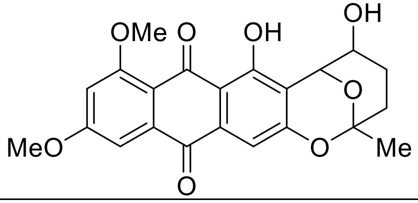			
AQ028	(-)-aversin 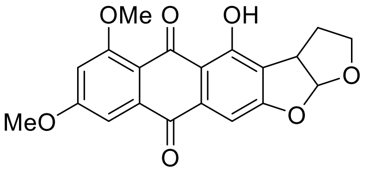	*E. coli* (zone of inhibition 0 mm) *S. aureus* (zone of inhibition 0 mm)		
AQ029	6,8-di-*O*-methylversicolorin A 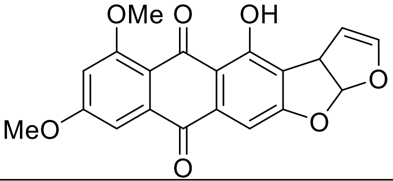	*E. coli* (zone of inhibition 0 mm) *S. aureus* (zone of inhibition 0 mm)		
AQ030	Coniothyrinone A 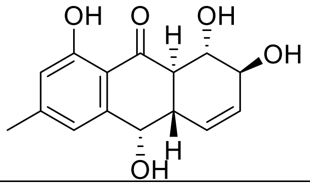	*V. parahaemolyticus* (ATCC 17802) (MIC = 6.25 μM) *P. putida* (ATCC 17848) (MIC = 3.13 μM) *V. anguillarum* (ATCC 19019) (MIC = 1.56 μM)	*Trichoderma* sp. isolated from a marine clam collected in the Yellow Sea, Chengshantou Island, Weihai City, China.	[7]
AQ031	Lentisone	*V. parahaemolyticus* (ATCC 17802) (MIC = 12.5 μM) *P. putida* (ATCC 17848) (MIC = 6.25 μM) *V. anguillarum* (ATCC 19019) (MIC = 1.56 μM)		

	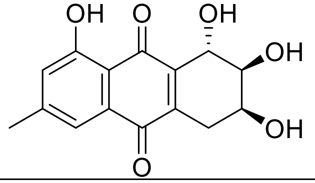			
AQ032	Emodin 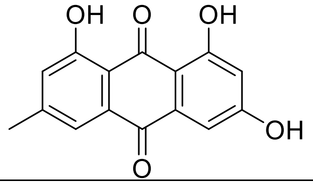	*P. putida* (ATCC 17848) (MIC = 25 μM)<br>*V. parahaemolyticus* (ATCC 17802) (MIC > 50 μM)<br>*V. anguillarum* (ATCC 19019) (MIC > 50 μM)		
AQ042	Monodictyquinone A 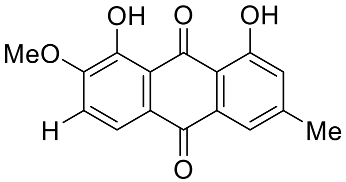	*B. subtilis* (zone of inhibition 7 mm)<br>*E. coli* (zone of inhibition 8 mm)<br>*B. subtilis* (zone of inhibition 10 mm)<br>*E. coli* (zone of inhibition 12 mm)<br>*B. subtilis* (zone of inhibition 15 mm)<br>*E. coli* (zone of inhibition 15 mm)	*Monodictys* sp. isolated from a sea urchin *Anthocidaris crassispina* collected in the Sea of Japan, Toyama Bay, Japan.	[9]
AQ043	3-*O*-(*α*-D-ribofuranosyl) questinol 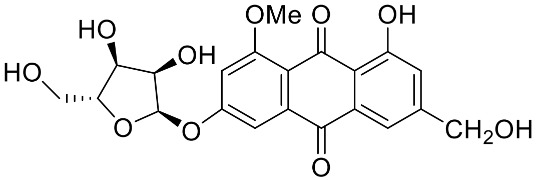	*E. coli* (MIC = 0 μg/mL)<br>*S. aureus* (MIC = 0 μg/mL)	*Eurotium cristatum* EN-220 isolated from a marine alga *Sargassum thunbergii* collected in Qingdao Coast, China.	[10]
AQ050	(+)-variecolorquinone A			

	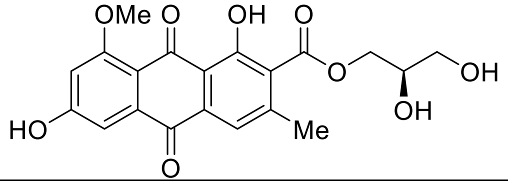			
AQ051	Saliniquinone G<br>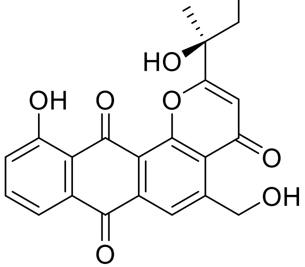	*Proteus sp.* (MIC = 12.5 µM)<br>MRCNS (MIC = 6.2 µM)<br>*B. subtilis* (MIC = 6.2 µM)<br>*B. cereus* (MIC = 6.2 µM)<br>*E. coli* (MIC = 6.2 µM)<br>*M. phlei* (MIC = 6.2 µM)		
AQ052	Saliniquinone H<br>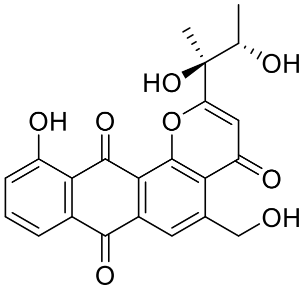	*Proteus sp.* (MIC = 6.2 µM)<br>MRCNS (MIC = 6.2 µM)<br>*B. subtilis* (MIC = 6.2 µM)<br>*B. cereus* (MIC = 6.2 µM)<br>*E. coli* (MIC = 6.2 µM)<br>*M. phlei* (MIC = 3.1 µM)	*Nocardiopsis aegyptia* HDN19-252 isolated from a marine animal sample collected in Antarctica.	[11]
AQ053	Saliniquinone I	*Proteus sp.* (MIC > 50 µM)<br>MRCNS (MIC > 50 µM)<br>*B. subtilis* (MIC > 50 µM)<br>*B. cereus* (MIC > 50 µM)<br>*E. coli* (MIC > 50 µM)<br>*M. phlei* (MIC > 50 µM)		

	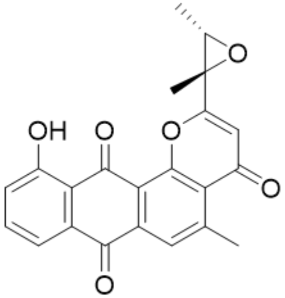			
AQ054	Heraclemycin E<br>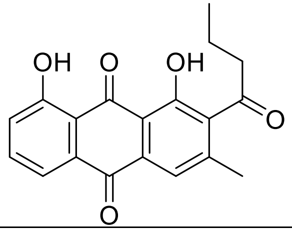	*Proteus sp.* (MIC > 50 µM)<br>MRCNS (MIC > 50 µM)<br>*B. subtilis* (MIC > 50 µM)<br>*B. cereus* (MIC > 50 µM)<br>*E. coli* (MIC > 50 µM)<br>*M. phlei* (MIC > 50 µM)		
AQ055	Urdamycinone E<br>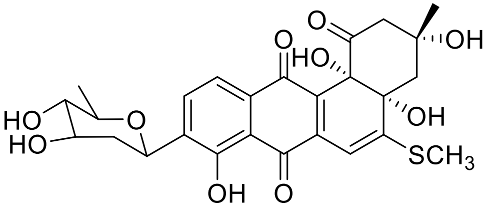	*M. tuberculosis* (H37Ra) (MIC = 3.13 µg/mL)	*Streptomyces* sp. BCC45596 isolated from the marine sponge *Xestospongia* sp. collected in the Sichang Island, Chonburi, Thailand.	[12]
AQ056	Urdamycinone G<br>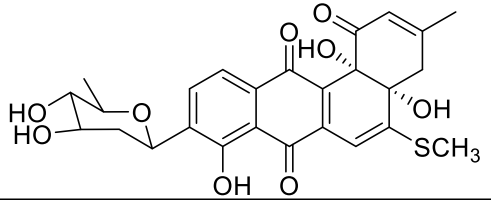	*M. tuberculosis* (H37Ra) (MIC = 12.50 µg/mL)		

AQ057	Dehydroxyaquayamycin 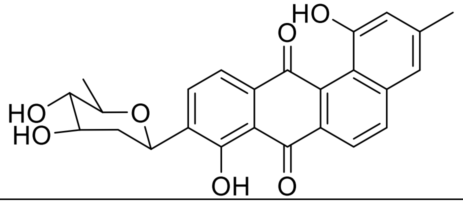	*M. tuberculosis* (H37Ra) (MIC = 6.25 µg/mL)		
AQ058	Urdamycin E 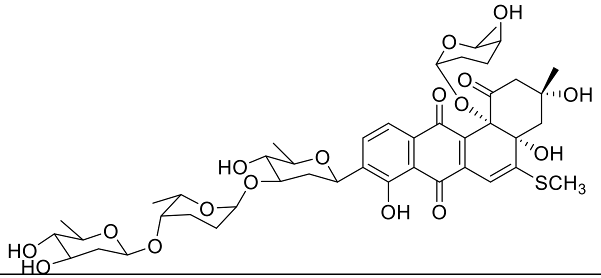	*M. tuberculosis* (H37Ra) (MIC = 12.50 µg/mL)		
AQ059	Penipurdin A 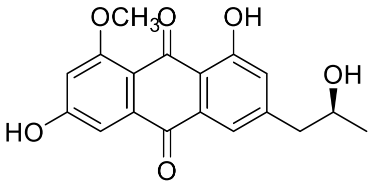	*E. faecalis* (ATCC 29212) (MIC > 64 µg/mL)<br>*E. faecalis* (B3/101) (MIC > 64 µg/mL)<br>*S. aureus* (ATCC 29213) (MIC > 64 µg/mL)<br>MRSA (MIC > 64 µg/mL)	*Neosartorya spinosa* KUFA1047 isolated from the marine sponge *Mycale* sp. collected in Samae San Island, Chonburi province, Thailand.	[13]
AQ060	Acetylquestinol 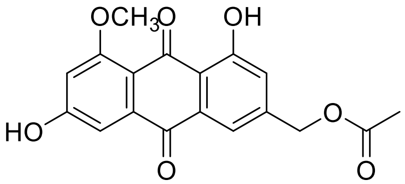	*S. aureus* (ATCC 25923) (MIC > 64 µg/mL)<br>*E. coli* (ATCC 25922) (MIC > 64 µg/mL)<br>*P. aeruginosa* (ATCC 27853) (MIC > 64 µg/mL)<br>*E. faecalis* (ATCC 29212) (MIC > 64 µg/mL)	*Neosartorya spinosa* KUFA1047 isolated from the marine sponge *Mycale* sp. collected in the Samae San Island, Chonburi province, Thailand.	[13,14]

AQ063	Trichodermaquinone 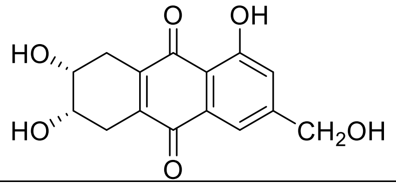	MRSA (MIC = 200 μg/mL)<br>*S. aureus* (MIC > 200 μg/mL)		
AQ064	Coniothranthraquinone 1 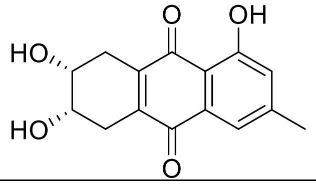	*S. aureus* (MIC = 16 μg/mL)<br>MRSA (MIC = 8 μg/mL)		
AQ065	Isorhodoptilometrin 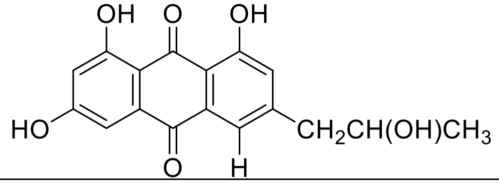	MRSA (MIC = 16 μg/mL)<br>*S. aureus* (MIC > 200 μg/mL)	*Trichoderma aureoviride* PSU-F95 isolated from the gorgonian sea fan *Annella* sp.	[15]
AQ066	Pachybasin 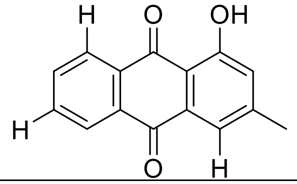	MRSA (MIC > 200 μg/mL)<br>*S. aureus* (MIC > 200 μg/mL)		
AQ067	ω-hydroxyemodin (citreorosein)	*S. aureus* (MIC = 200 μg/mL)<br>MRSA (MIC = 32 μg/mL)		

	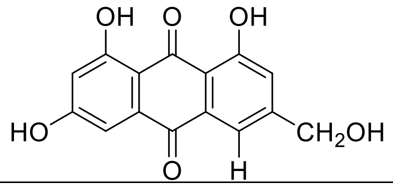			
AQ068	2-(dimethoxymethyl)-1-hydroxyanthracene-9,10-dione<br>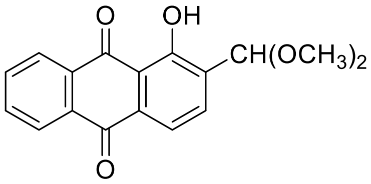	*V. rotiferianus* (MCCC E385) (MIC = 62.5 μg/mL)<br>*V. vulnificus* (MCCC E1758) (MIC = 31.3 μg/mL)<br>*V. campbellii* (MCCC E333) (MIC = 15.6 μg/mL)<br>MRSA (CGMCC 1.12409) (MIC = 7.8 μg/mL)<br>MRSA (ATCC 43300) (MIC = 3.9 μg/mL)		
AQ069	Damnacanthal<br>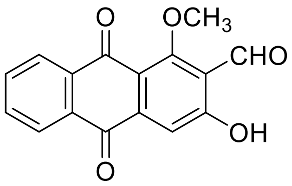	*V. campbellii* (MCCC E333) (MIC = 125 μg/mL)<br>*V. vulnificus* (MCCC E1758) (MIC = 62.5 μg/mL)<br>*V. rotiferianus* (MCCC E385) (MIC = 62.5 μg/mL)<br>MRSA (ATCC 43300) (MIC = 62.5 μg/mL)<br>MRSA (CGMCC 1.12409) (MIC = 31.3 μg/mL)	*Aspergillus versicolor* isolated from a marine sediment collected in the West Pacific Ocean (141.7608°E, 9.7942°N).	[16]
AQ070	Xanthopurpurin	*V. rotiferianus* (MCCC E385) (MIC = 125 μg/mL)<br>MRSA (CGMCC 1.12409) (MIC = 125 μg/mL)		

	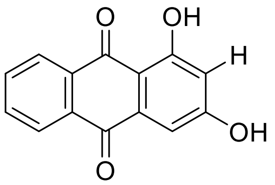	*V. vulnificus* (MCCC E1758) (MIC = 62.5 μg/mL)<br>*V. campbellii* (MCCC E333) (MIC = 62.5 μg/mL)<br>MRSA (ATCC 43300) (MIC = 62.5 μg/mL)		
AQ071	Methyl-averantin<br>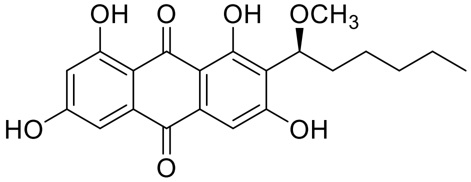	*S. pyogenes* (308A) (MIC = 6.25 μg/mL)<br>*S. aureus* (285) (MIC > 12.5 μg/mL)<br>*S. aureus* (503) (MIC > 12.5 μg/mL)<br>*S. pyogenes* (77A) (MIC > 12.5 μg/mL)<br>*S. aureus* (SG511) (MIC > 12.5 μg/mL)		
AQ072	Averufin<br>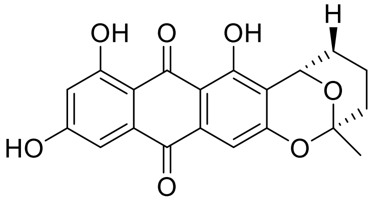	*S. aureus* (503) (MIC = 6.25 μg/mL)<br>*S. pyogenes* (308A) (MIC = 6.25 μg/mL)<br>*S. aureus* (285) (MIC > 12.5 μg/mL)<br>*S. pyogenes* (77A) (MIC > 12.5 μg/mL)<br>*S. aureus* (SG511) (MIC > 12.5 μg/mL)	*Aspergillus versicolor* isolated from the marine sponge *Petrosia* sp. collected in Jeju Island, Korea.	[17]
AQ073	Nidurufin<br>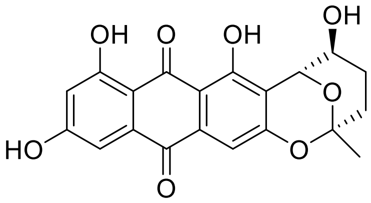	*S. pyogenes* (77A) (MIC = 6.25 μg/mL)<br>*S. aureus* (SG511) (MIC = 6.25 μg/mL)<br>*S. aureus* (285) (MIC = 3.13 μg/mL)<br>*S. aureus* (503) (MIC = 3.13 μg/mL)<br>*S. pyogenes* (308A) (MIC = 3.13 μg/mL)		

AQ074	Tetrahydrobostrycin 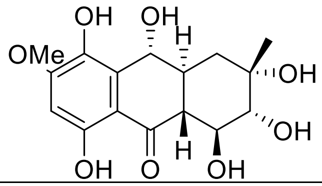	*E. coli* (IAM 12119T) (zone of inhibition 9.2 mm)<br>*S. aureus* (IAM 12544T) (zone of inhibition 15 mm)	*Aspergillus* sp. 05F16 isolated from an alga collected in a coral reef in Manado, Indonesia.	[18]
AQ075	1-deoxytetrahydrobostrycin 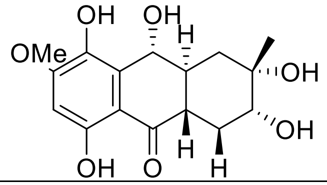	*E. coli* (IAM 12119T) (zone of inhibition 0 mm)<br>*S. aureus* (IAM 12544T) (zone of inhibition 12 mm)		
AQ076	Karimunone A 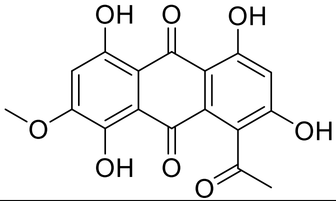	*S. enterica* ser. Typhi (MDR) (MIC = 0 µg/mL)	*Fusarium* sp. KJMT.FP.4.3 isolated from the sponge *Xestospongia* sp. collected in the Karimunjawa National Park, Central Java, Indonesia.	[19]
AQ077	Boshramycinone A 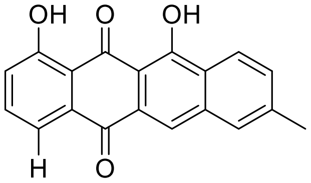	*B. subtilis* (ATCC 6051) (zone of inhibition 0 mm)<br>*S. viridochromogenes* (Tü 57) (zone of inhibition 0 mm)<br>*E. coli* (zone of inhibition 0 mm)<br>*S. aureus* (zone of inhibition 0 mm)	*Streptomyces* sp. Mei 16-1,2 isolated from a soil sample collected in Wadden Sea, Caeciliengroden salty marshland, Jade Bay, Germany.	[20]
AQ078	Boshramycinone B	*B. subtilis* (ATCC 6051) (zone of inhibition 0 mm)<br>*S. viridochromogenes* (Tü 57) (zone of inhibition 0 mm)		

	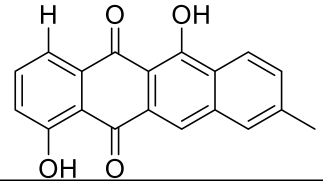	*E. coli* (zone of inhibition 0 mm) *S. aureus* (zone of inhibition 0 mm)		
AQ079	Boshramycinone C 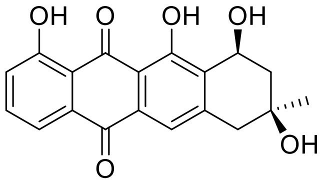	*B. subtilis* (ATCC 6051) (zone of inhibition 0 mm) *S. viridochromogenes* (Tü 57) (zone of inhibition 0 mm) *E. coli* (zone of inhibition 0 mm) *S. aureus* (zone of inhibition 0 mm)		
AQ080	2-acetyl-1,8-dihydroxy-3-methylanthraquinone 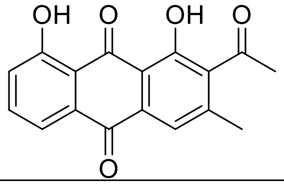	*E. coli* (zone of inhibition 18 mm) *B. subtilis* (ATCC6051) (zone of inhibition 14 mm) *S. aureus* (zone of inhibition 19 mm)	*Streptomyces* sp. Mei 16-1,2 isolated from a soil sample collected in Wadden Sea, Caeciliengroden salty marshland, Jade Bay, Germany.	[20,21]
AQ081	Isorhodoptilometrin-1-methyl ether 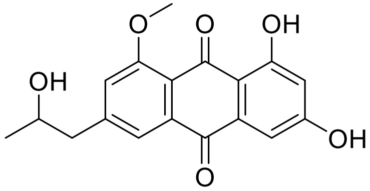	*B. megaterium* (zone of inhibition 0 mm) *E. cloacae* (zone of inhibition 0 mm) *K. pneumoniae* (zone of inhibition 0 mm) *E. coli* (zone of inhibition 0 mm) *B. cereus* (zone of inhibition 11 mm) *B. subtilis* (zone of inhibition 12 mm)	*Aspergillus versicolor* isolated from the marine alga *Halimeda opuntia* collected in the Egyptian Red Sea, Rass Mohamed, South Sinai, Egypt.	[22]

		S. aureus (zone of inhibition 14 mm)		
AQ082	1-Methyl emodin 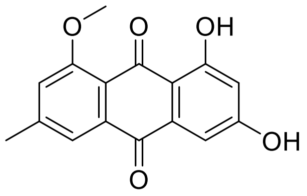	*B. megaterium* (zone of inhibition 0 mm)<br>*E. cloacae* (zone of inhibition 0 mm)<br>*K. pneumoniae* (zone of inhibition 0 mm)<br>*E. coli* (zone of inhibition 0 mm)<br>*B. cereus* (zone of inhibition 0 mm)<br>*B. subtilis* (zone of inhibition 0 mm)<br>*S. aureus* (zone of inhibition 0 mm)		
AQ088	Mersaquinone 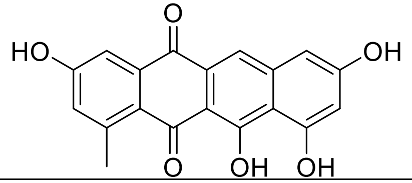	MRSA TCH1516 (MIC = 3.36 μg/mL)	*Streptomyces* sp. EG1 isolated from a sediment collected in the Mediterranean Sea, Mersa Matruh city, Egypt.	[23]
AQ089	Tetracenomycin D 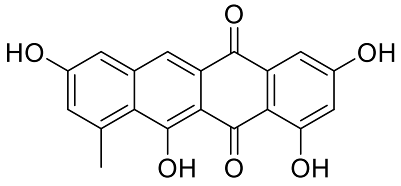	*S. aureus* (ATCC 29213) (MIC > 128 μg/mL)<br>*B. subtilis* (ATCC 6633) (MIC > 64 μg/mL)<br>*B. pumilus* (ATCC 19164) (MIC > 32 μg/mL)<br>*E. coli* (ATCC 26) (MIC > 128 μg/mL)<br>*P. aeruginosa* (ATCC 27853) (MIC > 128 μg/mL)	*Streptomyces corchorusii* $AUBN_1/7$ isolated from a marine sediment collected in the Machilipatnam coast, Bay of Bengal, India.	[24]

		P. vulgaris (ATCC 6897) (MIC > 64 μg/mL)		
AQ092	9-dehydroxyeurotinone 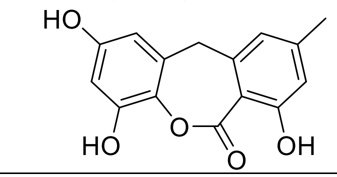	*E. coli* (zone of inhibition 7 mm) *S. aureus* (zone of inhibition 0 mm)	*Eurotium rubrum* G2 isolated from the mangrove plant *Hibiscus tiliaceus* Linn collected in Hainan Island, China.	[25]
AQ094	Emodic acid 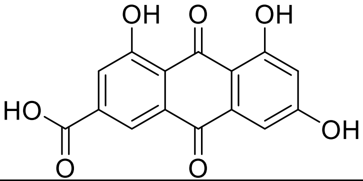	MRSA *S. aureus* (MIC = 0 μg/mL) *E. faecalis* (MIC = 0 μg/mL) *A. baumannii* (MIC = 0 μg/mL) *K. pneumoniae* (MIC = 0 μg/mL) *E. coli* (MIC = 0 μg/mL)	*Aspergillus sydowii* SCSIO41301 isolated from the marine sponge *Phakellia fusca* collected in Xisha Islands, China.	[25,26]
AQ095	4a-epi-9α-methoxydihydrodeoxybostrycin 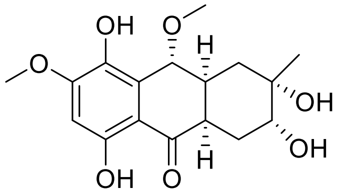	*B. cereus* (ACCC 11077) (MIC = 50 μM) *V. anguillarum* (ATCC 19019) (MIC = 25 μM) *V. parahemolyticus* (ATCC 17802) (MIC = 25 μM) *S. aureus* (ATCC 27154) (MIC = 1.56 μM) *M. tetragenus* (ATCC 13623) (MIC = 0.78 μM) *E. coli* (ATCC 25922) (MIC = 0.78 μM) *B. subtilis* (ATCC 6633) (MIC > 100 μM) *M. luteus* (ATCC 49732) (MIC > 100 μM)	*Nigrospora* sp. (ZJ-2010006) isolated from a sea anemone (GX-WZ-20100026) collected in the South China Sea, Weizhou coral reef, China.	[27]

		S. albus (ATCC 8799) (MIC > 100 μM)		
AQ098	9α-Hydroxydihydrodesoxybostrycin<br>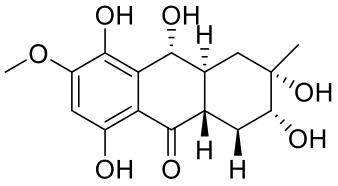 	*M. luteus* (ATCC 49732) (MIC = 25 μM)<br>*S. aureus* (ATCC 27154) (MIC = 25 μM)<br>*B. subtilis* (ATCC 6633) (MIC = 12.5 μM)<br>*B. cereus* (ACCC 11077) (MIC = 12.5 μM)<br>*V. parahemolyticus* (ATCC 17802) (MIC = 12.5 μM)<br>*E. coli* (ATCC 25922) (MIC = 6.25 μM)<br>*M. tetragenus* (ATCC 13623) (MIC = 1.56 μM)<br>*V. anguillarum* (ATCC 19019) (MIC = 1.56 μM)<br>*S. albus* (ATCC 8799) (MIC > 100 μM)		
AQ099	9α-Hydroxyhalorosellinia A<br>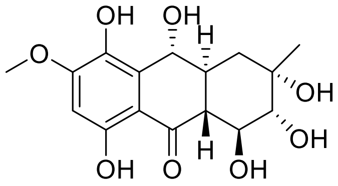 	*B. subtilis* (ATCC 6633) (MIC = 12.5 μM)<br>*B. cereus* (ACCC 11077) (MIC = 12.5 μM)<br>*M. tetragenus* (ATCC 13623) (MIC = 3.12 μM)<br>*V. anguillarum* (ATCC 19019) (MIC = 3.12 μM)<br>*V. parahemolyticus* (ATCC 17802) (MIC = 3.12 μM)		

		E. coli (ATCC 25922) (MIC = 1.56 μM)<br>*S. aureus* (ATCC 27154) (MIC = 0.78 μM)<br>*M. luteus* (ATCC 49732) (MIC > 100 μM)<br>*S. albus* (ATCC 8799) (MIC > 100 μM)		
AQ104	3,5,8-trihydroxy-7-methoxy-2-methylanthracene-9,10-dione<br>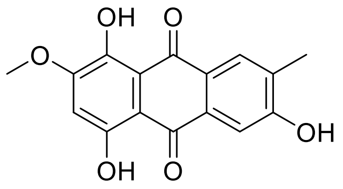	*B. subtilis* (ATCC 6633) (MIC > 200 μM)<br>*B. cereus* (ACCC 11077) (MIC > 200 μM)<br>*M. luteus* (ATCC 49732) (MIC > 200 μM)<br>*S. albus* (ATCC 8799) (MIC > 200 μM)<br>*M. tetragenus* (ATCC 13623) (MIC > 200 μM)<br>*E. coli* (ATCC 25922) (MIC > 200 μM)<br>*V. anguillarum* (ATCC 19019) (MIC > 200 μM)<br>*V. parahemolyticus* (ATCC 17802) (MIC > 200 μM)<br>*S. aureus* (ATCC 27154) (MIC > 200 μM)	*Nigrospora* sp. (ZJ-2010006) isolated from a sea anemone (GX-WZ-20100026) collected in the South China Sea, Weizhou coral reef, China.	
AQ105	8-Acetoxy-3,5-dihydroxy-7-methoxy-2-methylanthracene-9,10 dione	*S. albus* (ATCC 8799) (MIC = 100 μM)<br>*S. aureus* (ATCC 27154) (MIC = 100 μM)	Semi-synthetic compound obtained from AQ104.	

	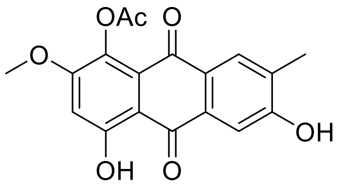	*B. subtilis* (ATCC 6633) (MIC > 200 μM)<br>*B. cereus* (ACCC 11077) (MIC > 200 μM)<br>*M. luteus* (ATCC 49732) (MIC > 200 μM)<br>*M. tetragenus* (ATCC 13623) (MIC > 200 μM)<br>*E. coli* (ATCC 25922) (MIC > 200 μM)<br>*V. anguillarum* (ATCC 19019) (MIC > 200 μM)<br>*V. parahemolyticus* (ATCC 17802) (MIC > 200 μM)		
AQ106	5-Acetoxy-3,8-dihydroxy-7-methoxy-2-methylanthracene-9,10-dione<br>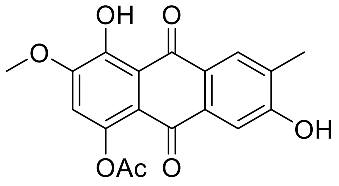	*B. subtilis* (ATCC 6633) (MIC > 200 μM)<br>*B. cereus* (ACCC 11077) (MIC > 200 μM)<br>*M. luteus* (ATCC 49732) (MIC > 200 μM)<br>*S. albus* (ATCC 8799) (MIC > 200 μM)<br>*M. tetragenus* (ATCC 13623) (MIC > 200 μM)<br>*E. coli* (ATCC 25922) (MIC > 200 μM)<br>*V. anguillarum* (ATCC 19019) (MIC > 200 μM)<br>*V. parahemolyticus* (ATCC 17802) (MIC > 200 μM)		

		S. aureus (ATCC 27154) (MIC > 200 μM)		
AQ107	3-Acetoxy-5,8-dihydroxy-7-methoxy-2-methylanthracene-9,10-dione 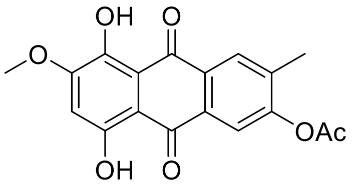	*B. cereus* (ACCC 11077) (MIC = 50 μM)<br>*B. subtilis* (ATCC 6633) (MIC = 12.5 μM)<br>*S. albus* (ATCC 8799) (MIC > 200 μM)<br>*S. aureus* (ATCC 27154) (MIC > 200 μM)<br>*M. luteus* (ATCC 49732) (MIC > 200 μM)<br>*M. tetragenus* (ATCC 13623) (MIC > 200 μM)<br>*E. coli* (ATCC 25922) (MIC > 200 μM)<br>*V. anguillarum* (ATCC 19019) (MIC > 200 μM)<br>*V. parahemolyticus* (ATCC 17802) (MIC > 200 μM)		
AQ108	5,8-Diacetoxy-3-hydroxy-7-methoxy-2-methylanthracene-9,10-dione 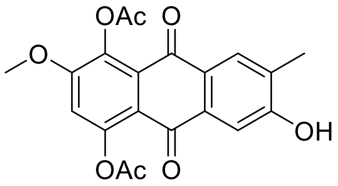	*V. parahemolyticus* (ATCC 17802) (MIC = 75 μM)<br>*B. cereus* (ACCC 11077) (MIC = 37.5 μM)<br>*M. tetragenus* (ATCC 13623) (MIC = 9.4 μM)<br>*B. subtilis* (ATCC 6633) (MIC = 5 μM)<br>*V. anguillarum* (ATCC 19019) (MIC = 4.7 μM)		

		M. luteus (ATCC 49732) (MIC > 150 μM)<br>*S. albus* (ATCC 8799) (MIC > 150 μM)<br>*E. coli* (ATCC 25922) (MIC > 150 μM)<br>*S. aureus* (ATCC 27154) (MIC > 150 μM)		
AQ109	3,8-Diacetoxy-5-hydroxy-7-methoxy-2-methylanthracene-9,10-dione<br>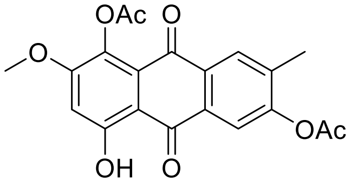	*M. luteus* (ATCC 49732) (MIC = 200 μM)<br>*M. tetragenus* (ATCC 13623) (MIC = 200 μM)<br>*B. subtilis* (ATCC 6633) (MIC > 200 μM)<br>*B. cereus* (ACCC 11077) (MIC > 200 μM)<br>*S. albus* (ATCC 8799) (MIC > 200 μM)<br>*E. coli* (ATCC 25922) (MIC > 200 μM)<br>*V. anguillarum* (ATCC 19019) (MIC > 200 μM)<br>*V. parahemolyticus* (ATCC 17802) (MIC > 200 μM)<br>*S. aureus* (ATCC 27154) (MIC > 200 μM)		
AQ110	3,5-Diacetoxy-8-hydroxy-7-methoxy-2-methylanthracene-9,10-dione	*B. subtilis* (ATCC 6633) (MIC = 50 μM)<br>*S. albus* (ATCC 8799) (MIC = 50 μM)		

	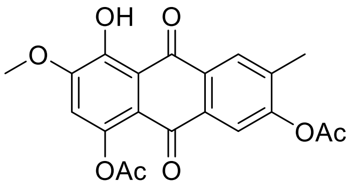	*M. luteus* (ATCC 49732) (MIC > 200 μM)<br>*M. tetragenus* (ATCC 13623) (MIC > 200 μM)<br>*B. cereus* (ACCC 11077) (MIC > 200 μM)<br>*E. coli* (ATCC 25922) (MIC > 200 μM)<br>*V. anguillarum* (ATCC 19019) (MIC > 200 μM)<br>*V. parahemolyticus* (ATCC 17802) (MIC > 200 μM)<br>*S. aureus* (ATCC 27154) (MIC > 200 μM)		
AQ111	2-methoxy-7-methyl-9,10-dioxo-9,10-dihydroanthracene-1,4,6-triyl triacetate<br>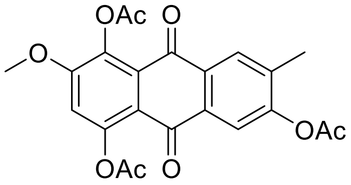	*B. cereus* (ACCC 11077) (MIC = 140 μM)<br>*M. luteus* (ATCC 49732) (MIC = 140 μM)<br>*V. anguillarum* (ATCC 19019) (MIC = 140 μM)<br>*S. albus* (ATCC 8799) (MIC = 70 μM)<br>*B. subtilis* (ATCC 6633) (MIC = 35 μM)<br>*M. tetragenus* (ATCC 13623) (MIC > 140 μM)<br>*E. coli* (ATCC 25922) (MIC > 140 μM)<br>*V. parahemolyticus* (ATCC 17802) (MIC > 140 μM)		

		S. aureus (ATCC 27154) (MIC > 140 μM)		
AQ112	Austrocortirubin<br>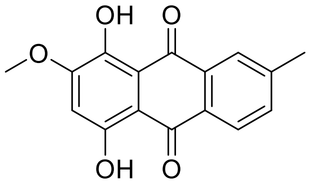	*B. subtilis* (ATCC 6633) (MIC > 200 μM)<br>*B. cereus* (ACCC 11077) (MIC > 200 μM)<br>*M. luteus* (ATCC 49732) (MIC > 200 μM)<br>*S. albus* (ATCC 8799) (MIC > 200 μM)<br>*M. tetragenus* (ATCC 13623) (MIC > 200 μM)<br>*E. coli* (ATCC 25922) (MIC > 200 μM)<br>*V. anguillarum* (ATCC 19019) (MIC > 200 μM)<br>*V. parahemolyticus* (ATCC 17802) (MIC > 200 μM)<br>*S. aureus* (ATCC 27154) (MIC > 200 μM)	*Nigrospora* sp. (ZJ-2010006) isolated from a sea anemone (GX-WZ-20100026) collected in the South China Sea, Weizhou coral reef, China.	
AQ113	8-Acetoxyaustrocortirubin<br>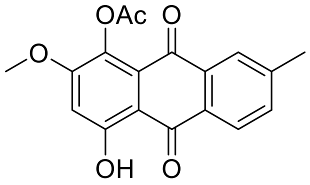	*B. subtilis* (ATCC 6633) (MIC > 200 μM)<br>*B. cereus* (ACCC 11077) (MIC > 200 μM)<br>*M. luteus* (ATCC 49732) (MIC > 200 μM)<br>*S. albus* (ATCC 8799) (MIC > 200 μM)<br>*M. tetragenus* (ATCC 13623) (MIC > 200 μM)	Semi-synthetic compound obtained from AQ112.	

		E. coli (ATCC 25922) (MIC > 200 μM)<br>*V. anguillarum* (ATCC 19019) (MIC > 200 μM)<br>*V. parahemolyticus* (ATCC 17802) (MIC > 200 μM)<br>*S. aureus* (ATCC 27154) (MIC > 200 μM)		
AQ114	Versicolorin B<br>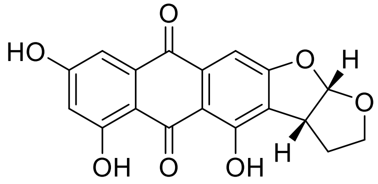 	MRSA (MRSAa) (MIC = 12.5 μg/mL)<br>*S. aureus* (ATCC 6538) (MIC = 6.25 μg/mL)<br>Bacillus Calmette–Guérin (Pasteur 1173P2) (MIC > 100 μg/mL)<br>*B. subtilis* (ATCC 6633) (MIC > 100 μg/mL)<br>*P. aeruginosa* (PAO1) (MIC > 100 μg/mL)	*Aspergillus versicolor* MF180151 isolated from a marine sediment collected in the Bohai Sea, China.	[28]
AQ123	Versiconol B<br>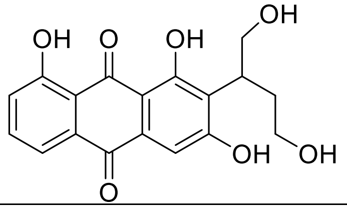 	*S. aureus* (ATCC 25923) (MIC = 48 μg/mL)<br>*V. parahemolyticus* (ATCC 17802) (MIC = 24 μg/mL)	*Aspergillus* sp. F40 isolated from the marine sponge *Callyspongia* sp.	[29]
AQ125	Lunatin	*E. coli* (ATCC 25922) (zone of inhibition 9 mm)<br>*E. coli* (HBI 101) (zone of inhibition 8 mm)	*Curvularia lunata* isolated from the marine sponge *Niphates olemda* collected in the Bali Bata National Park, Indonesia.	[30]

	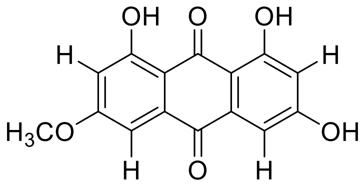	*B. subtilis* (168) (zone of inhibition 7.5 mm)<br>*S. aureus* (ATCC 25923) (zone of inhibition 8.5 mm)<br>*E. coli* (ATCC 25922) (zone of inhibition 11 mm)<br>*E. coli* (HBI 101) (zone of inhibition 10.5 mm)<br>*B. subtilis* (168) (zone of inhibition 9 mm)<br>*S. aureus* (ATCC 25923) (zone of inhibition 10 mm)		
AQ126	Cytoskyrin A<br>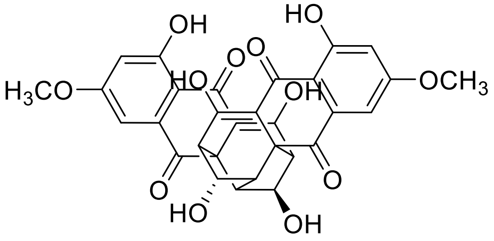	*E. coli* (ATCC 25922) (zone of inhibition 9 mm)<br>*E. coli* (HBI 101) (zone of inhibition 8 mm)<br>*B. subtilis* (168) (zone of inhibition 8 mm)<br>*S. aureus* (ATCC 25923) (zone of inhibition 8.5 mm)<br>*E. coli* (ATCC 25922) (zone of inhibition 11 mm)<br>*E. coli* (HBI 101) (zone of inhibition 9 mm)<br>*B. subtilis* (168) (zone of inhibition 12 mm)<br>*S. aureus* (ATCC 25923) (zone of inhibition 10 mm)		
AQ127	4-acetylchrysophanol	MRSA (669) (MIC > 128 μg/mL)<br>MRSA (991) (MIC > 128 μg/mL)<br>MRSA (1862) (MIC > 128 μg/mL)	*Streptomyces sampsonii* SCSIO 054 isolated from the marine gastropod	[31]

	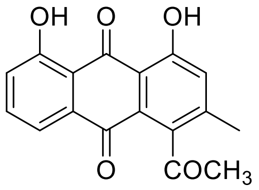	MRSA (GDE4P037P) (MIC > 128 μg/mL)<br>*S. aureus* (cfr, GDQ6P012P) (MIC > 128 μg/mL)<br>*S. aureus* (ATCC 29213) (MIC > 128 μg/mL)<br>*M. luteus* (MIC > 128 μg/mL)<br>*B. subtilis* (MIC > 128 μg/mL)<br>*S. simulans* (AKA1) (MIC > 128 μg/mL)	*Batillaria zonalis* collected in the South China Sea, Daya Bay, China.	
AQ128	Islandicin<br>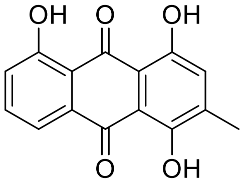	MRSA (669) (MIC > 128 μg/mL)<br>MRSA (991) (MIC > 128 μg/mL)<br>MRSA (1862) (MIC > 128 μg/mL)<br>MRSA (GDE4P037P) (MIC > 128 μg/mL)<br>*S. aureus* (cfr, GDQ6P012P) (MIC > 128 μg/mL)<br>*S. aureus* (ATCC 29213) (MIC > 128 μg/mL)<br>*M. luteus* (MIC > 128 μg/mL)<br>*B. subtilis* (MIC > 128 μg/mL)<br>*S. simulans* (AKA1) (MIC > 128 μg/mL)		
AQ129	Huanglongmycin A<br>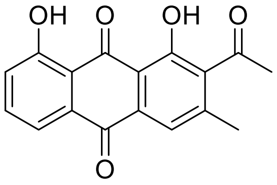	MRSA (669) (MIC > 128 μg/mL)<br>MRSA (991) (MIC > 128 μg/mL)<br>MRSA (1862) (MIC > 128 μg/mL)<br>MRSA (GDE4P037P) (MIC > 128 μg/mL)<br>*S. aureus* (cfr, GDQ6P012P) (MIC > 128 μg/mL)		

		S. aureus (ATCC 29213) (MIC > 128 μg/mL)<br>*M. luteus* (MIC > 128 μg/mL)<br>*B. subtilis* (MIC > 128 μg/mL)<br>*S. simulans* (AKA1) (MIC > 128 μg/mL)		
AQ130	Isoversicolorin B<br>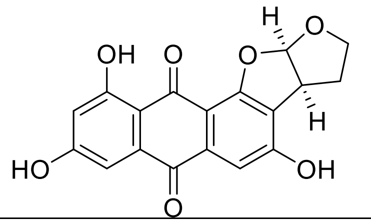	*S. aureus* (ATCC 29213) (MIC = 0 μM)<br>*E. faecalis* (ATCC 29212) (MIC = 0 μM)<br>*E. faecalis* (ATCC 51299) (MIC = 0 μM)<br>*E. faecium* (ATCC 35667) (MIC = 0 μM)<br>*E. faecium* (ATCC 700221) (MIC = 0 μM)	Co-culture of *Aspergillus versicolor*, isolated from the marine sponge *Agelas oroides* and collected in Aliağa-İzmir, Turkey, with *Bacillus subtilis* 168 trpC2.	[32]
AQ131	6,8-O-Dimethylbipolarin<br>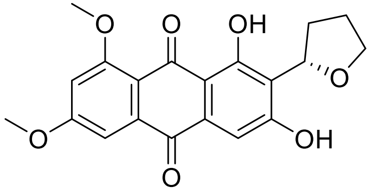			
AQ132	Deoxynyboquinone<br>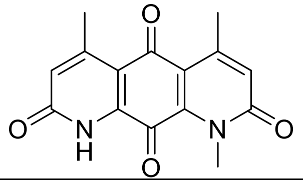	*S. aureus* (ATCC 29213) (MIC = 1 μg/mL)<br>*E. faecalis* (ATCC 29212) (MIC = 1 μg/mL)<br>*B. thuringensis* (SCSIO BT01) (MIC = 1 μg/mL)	*Pseudonocardia sp.* SCSIO 01299 isolated from a marine sediment collected in the South China Sea.	[33]
AQ133	Pseudonocardian A	*S. aureus* (ATCC 29213) (MIC = 4 μg/mL)<br>*E. faecalis* (ATCC 29212) (MIC = 2 μg/mL)		

	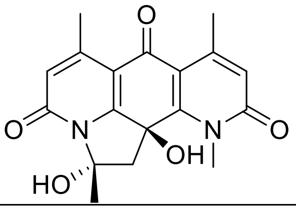	*B. thuringensis* (SCSIO BT01) (MIC = 4 μg/mL)		
AQ134	Pseudonocardian B 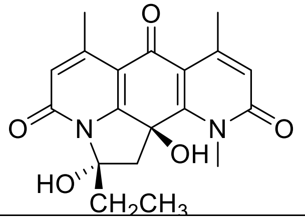	*S. aureus* (ATCC 29213) (MIC = 2 μg/mL) *E. faecalis* (ATCC 29212) (MIC = 2 μg/mL) *B. thuringensis* (SCSIO BT01) (MIC = 2 μg/mL)		
AQ136	Chrysophanol 8-methyl ether 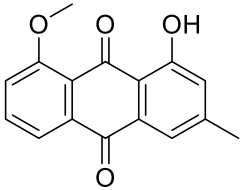	*E. coli* (ATCC 10536) (MIC = 10 μg/mL) *M. smegmatis* (ATCC 607) (MIC = 9.5 μg/mL) *C. xerosis* (NRRL B-1397) (MIC = 8 μg/mL) *P. aeruginosa* (ATCC10145) (MIC = 6 μg/mL) *M. luteus* (ATCC 9341) (MIC = 4.5 μg/mL) *S. aureus* (ATCC 6538) (MIC = 3 μg/mL) *B. subtilis* (ATCC 6051) (MIC = 2 μg/mL) *B. cereus* (ATCC 9634) (MIC = 1.5 μg/mL)	*Nocardia* sp. ALAA 2000 isolated from the marine alga *Laurenica spectabilis* collected in the Red Sea, Ras-Gharib coast, Egypt.	[34]
AQ137	Asphodelin	*E. coli* (ATCC 10536) (MIC = 8 μg/mL)		

	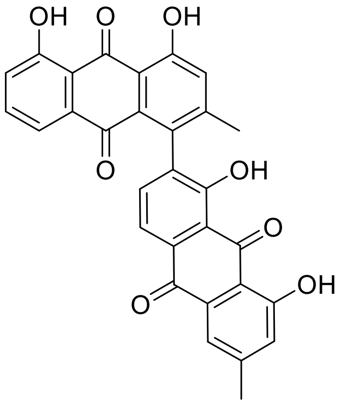	*P. aeruginosa* (ATCC10145) (MIC = 6.5 μg/mL)<br>*M. smegmatis* (ATCC 607) (MIC = 6.5 μg/mL)<br>*C. xerosis* (NRRL B-1397) (MIC = 3 μg/mL)<br>*B. cereus* (ATCC 9634) (MIC = 2.5 μg/mL)<br>*S. aureus* (ATCC 6538) (MIC = 1.6 μg/mL)<br>*M. luteus* (ATCC 9341) (MIC = 1.2 μg/mL)<br>*B. subtilis* (ATCC 6051) (MIC = 0.5 μg/mL)		
AQ140	BE-43472B<br>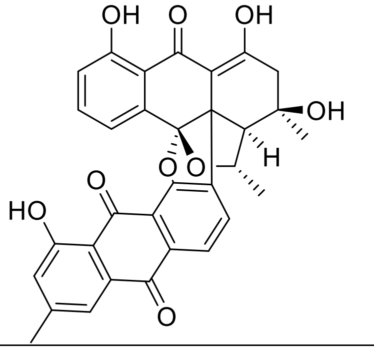	*E. faecium* (VRE) (MIC = 0.24 μM)<br>MSSA (MIC = 0.05 to 0.11 μM)<br>MRSA (MIC = 0.11 to 0.45 μM)	*Streptomyces* sp. N1-78-1 isolated from a marine alga collected from the tunicate *E. turbinata* collected in La Paragua, Puerto Rico.	[35]
AQ141	BE-43472A	MSSA (MIC = 0.22 to 0.88 μM)<br>*E. faecium* (VRE) (MIC = 0.44 to 1.8 μM)<br>MRSA (MIC = 0.44 to 0.88 μM)		

	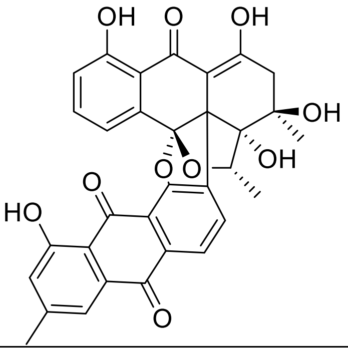			
AQ142	Bisanthraquinone metabolite 3 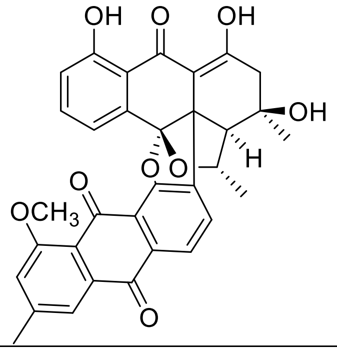	*E. faecium* (VRE) (MIC = 56 μM)<br>MSSA (MIC = 7.1 to 14 μM)<br>MRSA (MIC = 7.1 to 14 μM)		
AQ143	Bisanthraquinone metabolite 4 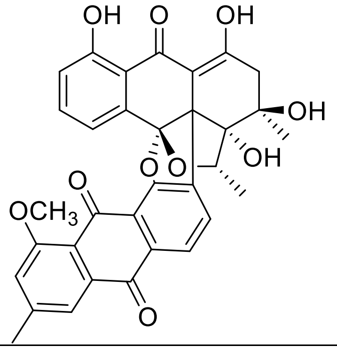	*E. faecium* (VRE) (MIC = 56 μM)<br>MSSA (MIC = 0.86 to >55 μM)<br>MRSA (MIC = 0.86 to >55 μM)		

AQ144	Bisanthraquinone derivative 5 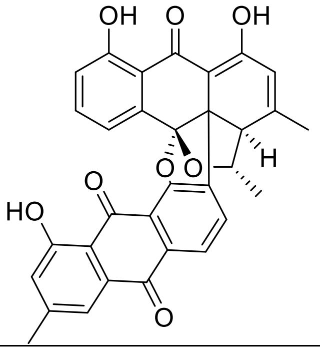	MSSA (MIC = 7.5 μM)<br>MRSA (MIC = 7.5 μM)<br>*E. faecium* (VRE) (MIC > 60 μM)	Semi-synthetic compound obtained from AQ140.	
AQ145	Bisanthraquinone derivative 6 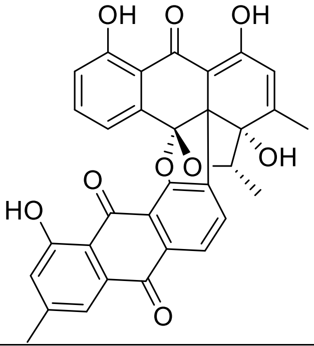	*E. faecium* (VRE) (MIC = 1.8 μM)<br>MRSA (MIC = 0.11 μM)<br>MSSA (MIC = 0.11 to 0.22 μM)	Semi-synthetic compound obtained from AQ141.	
AQ146	Bisanthraquinone derivative 7	MSSA (MIC = 1.8 to 3.6 μM)<br>*E. faecium* (VRE) (MIC = 15 to 29 μM)<br>MRSA (MIC = 0.23 to 0.91 μM)	Semi-synthetic compound obtained from AQ144.	

	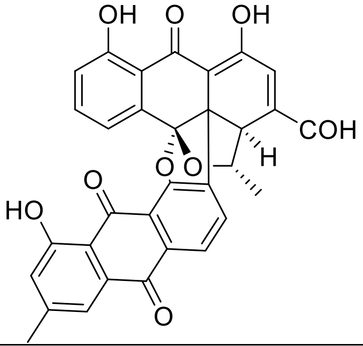			
AQ147	Bisanthraquinone derivative 8<br>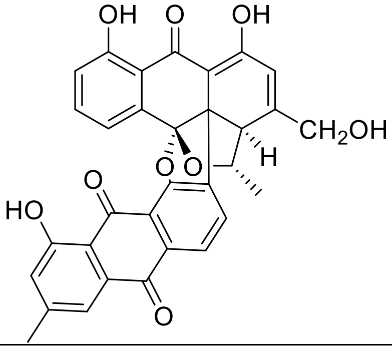	MSSA (MIC = 0.23 μM)<br>*E. faecium* (VRE) (MIC = 0.23 μM)<br>MRSA (MIC = 0.23 to 0.91 μM)	Semi-synthetic compound obtained from AQ145.	
AQ148	Grincamycin I<br>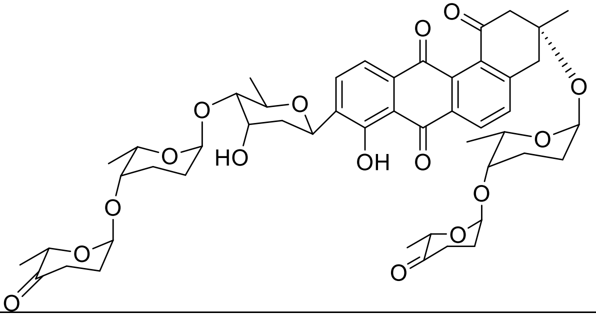	*S. aureus* (ATCC 29213) (MIC > 100 μg/mL)<br>*B. thuringiensis* (MIC > 100 μg/mL)<br>*M. luteus* (MIC > 100 μg/mL)<br>*S. aureus* (shhs-A1) (MIC > 100 μg/mL)	*Streptomyces lusitanus* SCSIO LR32 isolated from a marine sediment in the South China Sea.	[36]

AQ149	Grincamycin J 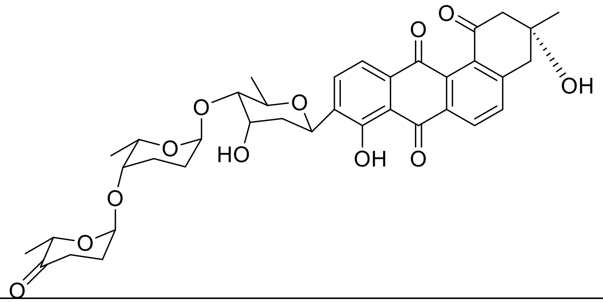	*S. aureus* (ATCC 29213) (MIC > 100 μg/mL) *B. thuringiensis* (MIC > 100 μg/mL) *M. luteus* (MIC > 100 μg/mL) *S. aureus* (shhs-A1) (MIC > 100 μg/mL)		
AQ150	Grincamycin K 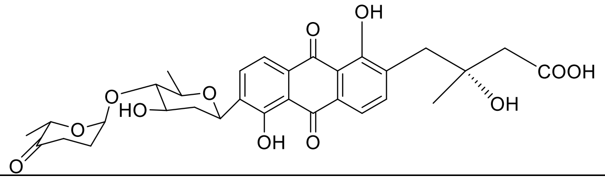	*S. aureus* (ATCC 29213) (MIC > 100 μg/mL) *B. thuringiensis* (MIC > 100 μg/mL) *M. luteus* (MIC > 100 μg/mL) *S. aureus* (shhs-A1) (MIC > 100 μg/mL)		
AQ151	A-7884 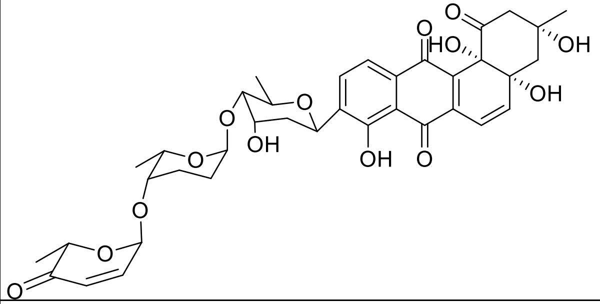	*M. luteus* (MIC = 1.95 μg/mL) *S. aureus* (ATCC 29213) (MIC > 100 μg/mL) *B. thuringiensis* (MIC > 100 μg/mL) *S. aureus* (shhs-A1) (MIC > 100 μg/mL)		
AQ152	Gephyyamycin	*S. aureus* (ATCC 25922) (MIC = 0 μM)	*Streptomyces* sp. HN-A124	[37]

	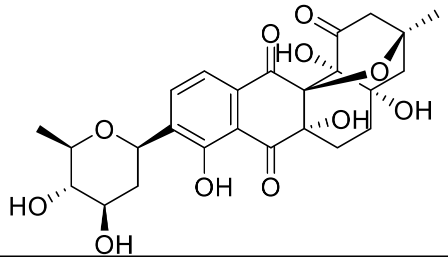		isolated from a marine sediment collected in Hainan Province, China.	
AQ153	Cysrabelomycin 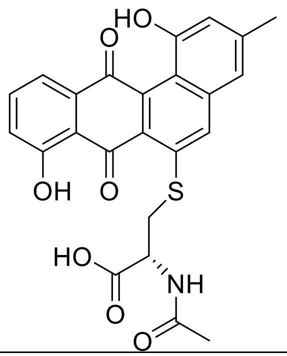	*S. aureus* (ATCC 25922) (MIC = 20 μM)		
AQ154	Erythroglaucin 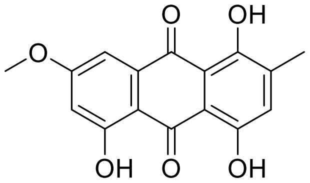	*P. glicinea* (growth inhibition of treated bacteria 25%) *P. fluorescens* (growth inhibition of treated bacteria 15%) *P. phaseolicola* (growth inhibition of treated bacteria 0%) *B. mycoides* (growth inhibition of treated bacteria 0%)	*Chaetomium globosum* isolated from the marine alga *Polysiphonia urceolata* collected in Qingdao, China.	[38,39]
AQ155	Parietin	*S. enterica* ser. Typhi (Clinically isolated - CI) (MIC = 62.5 μg/mL) *E. cloacae* (CI) (MIC = 62.5 μg/mL) *P. aeruginosa* (CI) (MIC = 62.5 μg/mL)		[38,40]

	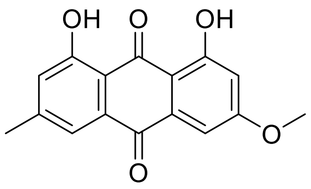	*K. pneumoniae* (Cl) (MIC = 62.5 μg/mL) *P. vulgaris* (CI) (MIC = 31.3 μg/mL) *P. mirabilis* (CI) (MIC = 31.3 μg/mL) *S. enterica* ser. Typhi (ATCC 19430) (MIC = 31.3 μg/mL) *E. cloacae* (ATCC 10699) (MIC = 31.3 μg/mL) *E. aerogenes* (CI) (MIC = 31.3 μg/mL) *P. aeruginosa* (ATCC 27853) (MIC = 31.3 μg/mL) *K. pneumoniae* (ATCC 27736) (MIC = 31.3 μg/mL) *S. aureus* (CI) (MIC = 15.6 μg/mL) *E. faecalis* (CI) (MIC = 15.6 μg/mL) *P. vulgaris* (ATCC 12454) (MIC = 15.6 μg/mL) *P. mirabilis* (ATCC 7002) (MIC = 15.6 μg/mL) *E. aerogenes* (ATCC 13048) (MIC = 15.6 μg/mL) *S. aureus* (ATCC 13709) (MIC = 7.8 μg/mL) *E. faecalis* (ATCC 14428) (MIC = 7.8 μg/mL)		
AQ156	7-chloro-1′-hydroxyisorhodoptilometrin	*E.coli* (MIC > 128 μg/mL) *P. aeruginosa* (MIC > 128 μg/mL) *A. baumannii* (MIC > 128 μg/mL)	*Penicillium* sp. SCSIO sof101 isolated from a marine sediment collected in the South China Sea.	[41]

	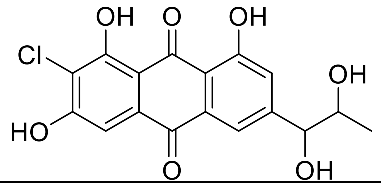	*S. aureus* (MIC > 128 μg/mL)		
BZ001	Iso-monodictyphenone<br>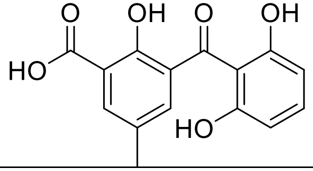	*A. hydrophilia* (MIC = 8 μg/mL)<br>*V.alginolyticus* (MIC = 0 μg/mL)<br>*V. anguillarum* (MIC = 0 μg/mL)<br>*V. harveyi* (MIC = 0 μg/mL)<br>*E. tarda* (MIC = 0 μg/mL)	*Penicillium* sp. MA-37 isolated from the mangrove plant *Bruguiera gymnorrhiza* collected in Hainan Island, China.	[42]
BZ002	Pestalachloride B<br>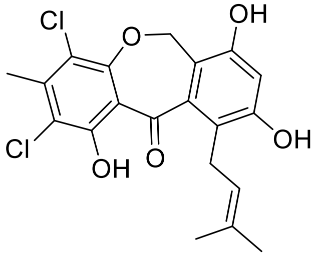	*B. subtilis* (ATCC 6633) (MIC = 3 μg/mL)<br>*S. aureus* (ATCC 25923) (MIC = 3 μg/mL)<br>*E. coli* (ATCC 25922) (MIC > 100 μg/mL)	*Pestalotiopsis heterocornis* isolated from the sponge *Phakellia fusca* collected in Xisha Islands, China.	[43]
BZ004	Monochlorsulochrin<br>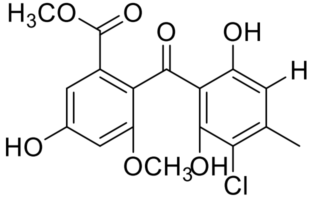	*S. aureus* (ATCC 25923) (MIC = 1.56 μg/mL)<br>*S. aureus* (ATCC 33591) (MIC = 1.56 μg/mL)<br>*S. aureus* (ATCC 29213) (MIC = 3.13 μg/mL)<br>*S. aureus* (ATCC 43300) (MIC = 12.5 μg/mL)<br>*E. faecalis* (ATCC51299) (MIC = 50 μg/mL)	*Aspergillus flavipes* DL-11 isolated from a marine sediment collected in Dalian, Liaoning province, China.	[44]

		E. faecium (ATCC35667) (MIC = 100 µg/mL)<br>*V. parahemolyticus* (ATCC17802) (MIC > 100 µg/mL)		
BZ005	Dihydrogeodin<br>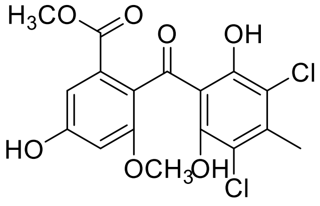 	*S. aureus* (ATCC 25923) (MIC = 1.56 µg/mL)<br>*S. aureus* (ATCC 33591) (MIC = 1.56 µg/mL)<br>*S. aureus* (ATCC 29213) (MIC = 3.13 µg/mL)<br>*S. aureus* (ATCC 43300) (MIC = 6.25 µg/mL)<br>*E. faecalis* (ATCC51299) (MIC = 12.5 µg/mL)<br>*E. faecium* (ATCC35667) (MIC = 12.5 µg/mL)<br>*V. parahemolyticus* (ATCC17802) (MIC > 100 µg/mL)		
BZ008	2,2',3,5-Tetrahydroxy-3'-methylbenzophenone<br>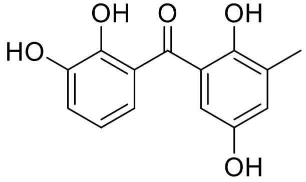 	*E.coli* (MIC = 4 µg/mL)<br>*P. aeruginosa* (MIC = 4 µg/mL)<br>*V. alginolyticus* (MIC = 4 µg/mL)<br>*V. parahaemolyticus* (MIC = 4 µg/mL)<br>*V. harveyi* (MIC = 8 µg/mL)<br>*S. aureus* (MIC = 8 µg/mL)	*Talaromyces islandicus* EN-501 isolated from the marine alga *Laurencia okamurai* collected in Qingdao, China.	[45]
BZ009	2,2′,5′-Trihydroxy-3-methoxy-3'-methylbenzophenone	*E.coli* (MIC > 64 µg/mL)<br>*P. aeruginosa* (MIC > 64 µg/mL)<br>*V. alginolyticus* (MIC > 64 µg/mL)<br>*V. parahaemolyticus* (MIC > 64 µg/mL)<br>*V. harveyi* (MIC > 64 µg/mL)		

	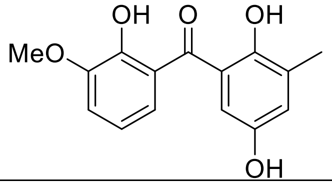	*S. aureus* (MIC > 64 μg/mL)		
BZ010	Penibenzophenone C<br>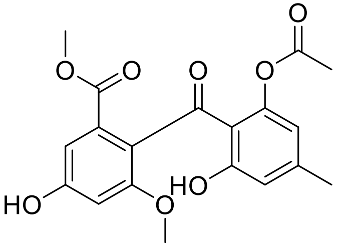	MRSA (ATCC 33951) (MIC = 3.12 μg/mL)<br>*S. aureus* (ATCC 27154) (MIC = 6.25 μg/mL)<br>*B. cereus* (ATCC 11778) (MIC = 12.5 μg/mL)<br>*E. coli* (ATCC 25922) (MIC > 25 μg/mL)<br>*S. albus* (ATCC 8799) (MIC > 25 μg/mL)	*Penicillium* sp. isolated from the mangrove plant *Acanthus ilicifolius* L. collected in the Beibu gulf, Guangxi Province, China.	[46]
BZ011	Penibenzophenone D<br>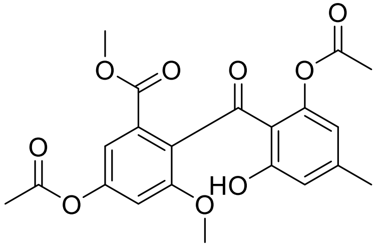	MRSA (ATCC 33951) (MIC = 6.25 μg/mL)<br>*S. aureus* (ATCC 27154) (MIC = 12.5 μg/mL)<br>*E. coli* (ATCC 25922) (MIC = 25 μg/mL)<br>*S. albus* (ATCC 8799) (MIC = 25 μg/mL)<br>*B. cereus* (ATCC 11778) (MIC > 25 μg/mL)		

BZ012	Sulochrin 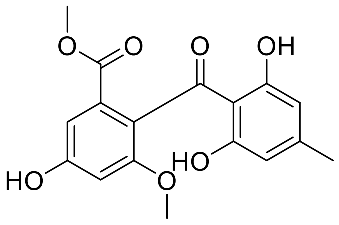	*S. aureus* (ATCC 27154) (MIC = 25 μg/mL) MRSA (ATCC 33951) (MIC = 25 μg/mL) *S. albus* (ATCC 8799) (MIC = 25 μg/mL) *B. cereus* (ATCC 11778) (MIC = 25 μg/mL) *E. coli* (ATCC 25922) (MIC > 25 μg/mL)		
BZ013	Hydoxysulochrin 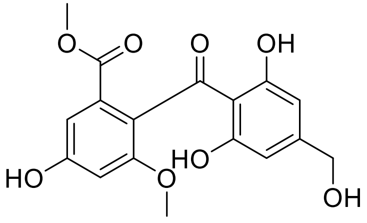	*S. aureus* (ATCC 27154) (MIC = 6.25 μg/mL) *E. coli* (ATCC 25922) (MIC = 12.5 μg/mL) *B. cereus* (ATCC 11778) (MIC = 12.5 μg/mL) MRSA (ATCC 33951) (MIC > 25 μg/mL) *S. albus* (ATCC 8799) (MIC > 25 μg/mL)		
BZ014	Pestalone 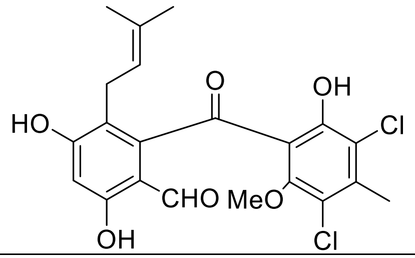	*E. faecium* (MIC = 0.078 μg/mL) MRSA (MIC = 0.037 μg/mL)	Co-culture of *Pestalotia* sp. CNL365, isolated from the marine alga *Rosenvingea* sp. collected in Bahamas Islands, with an unidentified bacterium.	[47]

BZ015	Penibenzophenone A 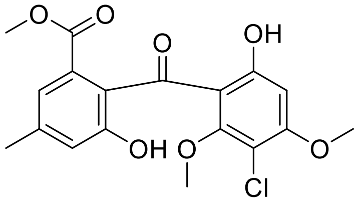	*E. coli* (ATCC 25922) (MIC = 0 μg/mL)<br>*B. cereus* (ATCC 11778) (MIC = 0 μg/mL)<br>*S. epidermis* (ATCC 12228) (MIC = 0 μg/mL)<br>*S. albus* (ATCC 8799) (MIC = 0 μg/mL)<br>*S. aureus* (ATCC 25923) (MIC = 20 μg/mL)	*Penicillium citrinum* HL-5126 isolated from the mangrove plant *Bruguiera sexangula var. rhynchopetala* collected in the South China Sea.	[48]
BZ016	Penibenzophenone B 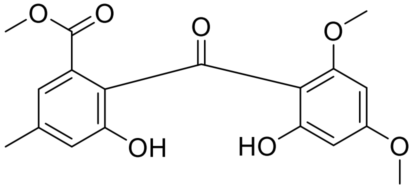	*E. coli* (ATCC 25922) (MIC = 0 μg/mL)<br>*B. cereus* (ATCC 11778) (MIC = 0 μg/mL)<br>*S. epidermis* (ATCC 12228) (MIC = 0 μg/mL)<br>*S. albus* (ATCC 8799) (MIC = 0 μg/mL)<br>*S. aureus* (ATCC 25923) (MIC = 0 μg/mL)		
DP001	Tenellic acid C 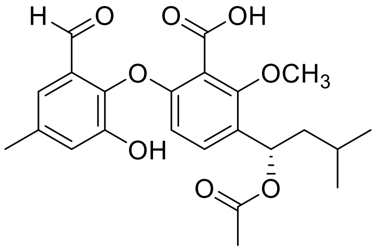	*S. aureus* (ATCC 29213) (MIC > 64 μg/mL)<br>MRSA (MIC > 64 μg/mL)<br>*E. faecalis* (ATCC 29212) (MIC > 64 μg/mL)<br>*E. faecalis* (B3/101, VRE) (MIC > 64 μg/mL)	*Neosartorya spinosa* KUFA 1047 isolated from the marine sponge *Mycale* sp. collected in Samae San Island, Chonburi province, Thailand.	[13]
DP002	Neospinosic Acid	*S. aureus* (ATCC 29213) (MIC > 64 μg/mL)<br>MRSA (MIC > 64 μg/mL)		

	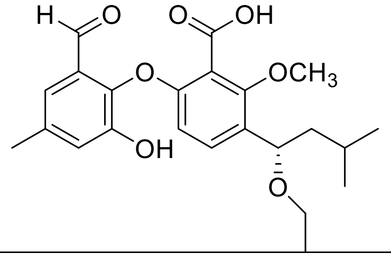	*E. faecalis* (ATCC 29212) (MIC > 64 µg/mL)<br>*E. faecalis* (B3/101, VRE) (MIC > 64 µg/mL)		
DP003	Circinophoric acid<br>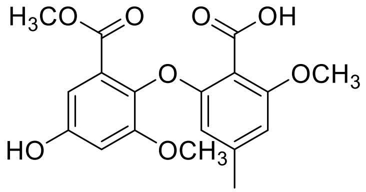	*S. aureus* (ATCC 25923) (MIC > 256 µg/mL)<br>*B. subtilis* (ATCC 6633) (MIC > 256 µg/mL)<br>*E. coli* (ATCC 25922) (MIC > 256 µg/mL)<br>*P. aeruginosa* (ATCC 27853) (MIC > 256 µg/mL)	*Sporidesmium circinophorum* KUFA 0043 isolated from the marine sponge *Petrosia* sp. collected in Samae San Island, Chonburi province, Thailand.	[49]
DP004	Diorcinol D<br>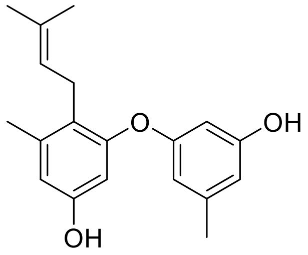	*E. faecium* (ATCC 35667) (MIC = 12.5 µM)<br>*S. aureus* (ATCC 29213) (MIC = 25 µM)<br>*E. faecalis* (ATCC 29212) (MIC = 50 µM)<br>*E. faecalis* (ATCC 51299) (MIC = 50 µM)<br>*E. faecium* (ATCC 700221) (MIC = 50 µM)	Co-culture of *Aspergillus versicolor*, isolated from the marine sponge *Agelas oroides* and collected in Aliağa-İzmir, Turkey, with *Bacillus subtilis* 168 trpC2.	[32]
DP005	Diorcinol G	*E. faecium* (ATCC 35667) (MIC = 12.5 µM)<br>*S. aureus* (ATCC 29213) (MIC = 12.5 µM)<br>*E. faecalis* (ATCC 29212) (MIC = 12.5 µM)		

	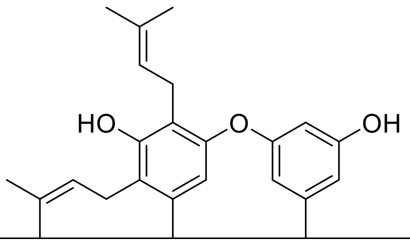	*E. faecalis* (ATCC 51299) (MIC = 12.5 μM)<br>*E. faecium* (ATCC 700221) (MIC = 12.5 μM)		
DP006	Diorcinol I<br>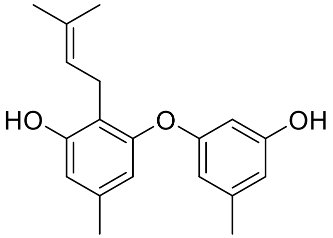	*E. faecium* (ATCC 35667) (MIC = 25 μM)<br>*S. aureus* (ATCC 29213) (MIC = 50 μM)<br>*E. faecalis* (ATCC 29212) (MIC = 50 μM)<br>*E. faecalis* (ATCC 51299) (MIC = 50 μM)<br>*E. faecium* (ATCC 700221) (MIC = 50 μM)		
DP007	Pseudogymnoascin A<br>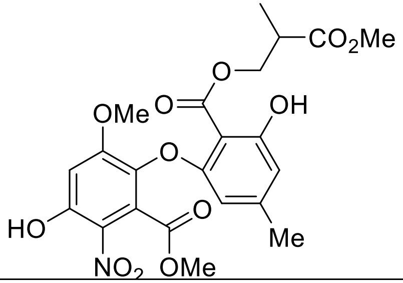	MSSA (EPI167) (MIC > 64 μg/mL)<br>MRSA (MB5393) (MIC > 64 μg/mL)<br>*P. aeruginosa* (PAO1) (MIC > 64 μg/mL)<br>A. *baumannii* (CL 5973) (MIC > 64 μg/mL)<br>*E. coli* (MB2884) (MIC > 64 μg/mL)	*Pseudogymnoascus* sp. F09-T18-1 isolated from the marine sponge *Hymeniacidon* sp. Collected in King George Island.	[50]
DP008	Pseudogymnoascin B	MSSA (EPI167) (MIC > 64 μg/mL)<br>MRSA (MB5393) (MIC > 64 μg/mL)<br>*P. aeruginosa* (PAO1) (MIC > 64 μg/mL)		

	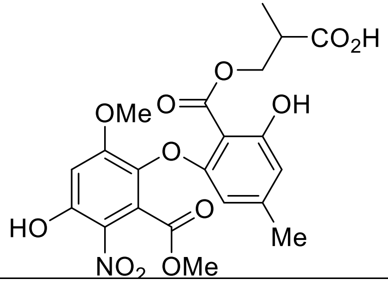	A. *baumannii* (CL 5973) (MIC > 64 μg/mL)<br>*E. coli* (MB2884) (MIC > 64 μg/mL)		
DP009	Pseudogymnoascin C<br>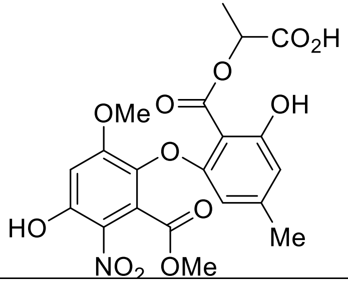	MSSA (EPI167) (MIC > 64 μg/mL)<br>MRSA (MB5393) (MIC > 64 μg/mL)<br>*P. aeruginosa* (PAO1) (MIC > 64 μg/mL)<br>A. *baumannii* (CL 5973) (MIC > 64 μg/mL)<br>*E. coli* (MB2884) (MIC > 64 μg/mL)		
DP010	3-Nitroasterric acid<br>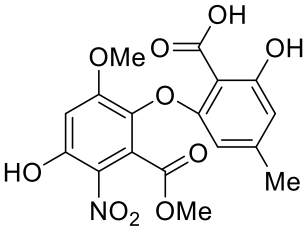	MSSA (EPI167) (MIC > 64 μg/mL)<br>MRSA (MB5393) (MIC > 64 μg/mL)<br>*P. aeruginosa* (PAO1) (MIC > 64 μg/mL)<br>A. *baumannii* (CL 5973) (MIC > 64 μg/mL)<br>*E. coli* (MB2884) (MIC > 64 μg/mL)		
DP011	Penikellide A	*V. alginolyticus* (MIC = 0 μg/mL)		[42]

	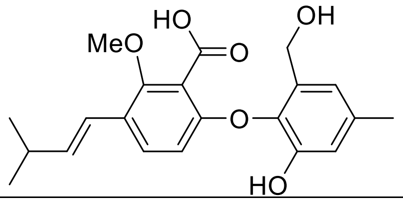	*V. anguillarum* (MIC = 0 µg/mL)<br>*V. harveyi* (MIC = 0 µg/mL)<br>*E. tarda* (MIC = 0 µg/mL)<br>*A. hydrophilia* (MIC = 0 µg/mL)	*Penicillium* sp. MA-37 isolated from the mangrove plant *Bruguiera gymnorrhiza* collected in Hainan Island, China.	
DP012	Penikellide B<br>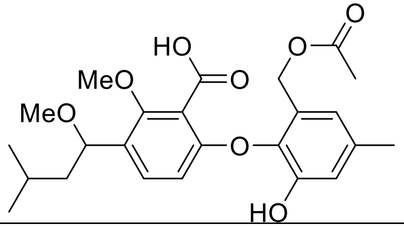			
DP013	2,3,5-tribromo-6-(3,5-dibromo-2-methoxyphenoxy)phenol<br>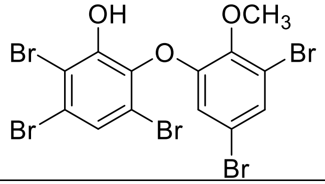	MRSA (ATCC 43300) (IC_{50} = 0.98 µM)<br>*E. coli* (ATCC 35218) (IC_{50} = 0 µM)<br>*P. aeruginosa* (ATCC 27853) (IC_{50} = 0 µM)<br>*M. intracellulare* (ATCC 23068) (IC_{50} = 0 µM)	*Lendenfeldia dendyi* collected in Papua New Guinea.	
DP014	2,3,4,5-tetrabromo-6-(3,5-dibromo-2-hydroxyphenoxy)phenol<br>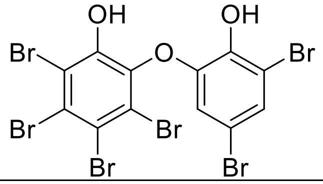	MRSA (ATCC 43300) (IC_{50} = 0.29 µM)<br>*E. coli* (ATCC 35218) (IC_{50} = 0 µM)<br>*P. aeruginosa* (ATCC 27853) (IC_{50} = 0 µM)<br>*M. intracellulare* (ATCC 23068) (IC_{50} = 0 µM)		[51]
DP015	2-(2-acetoxy-3,4,5,6-tetrabromophenoxy)-4,6-dibromophenyl acetate	MRSA (ATCC 43300) (IC_{50} = 1.18 µM)<br>*E. coli* (ATCC 35218) (IC_{50} = 0 µM)	Semi-syntetic compound obtained from DP014.	

	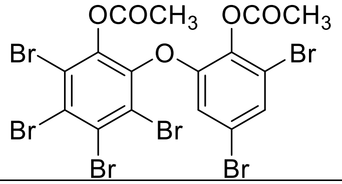	*P. aeruginosa* (ATCC 27853) (IC_{50} = 0 μM)<br>*M. intracellulare* (ATCC 23068) (IC_{50} = 0 μM)		
DP016	2,3,5-tribromo-6-(3,5-dibromo-2-hydroxyphenoxy)phenol<br>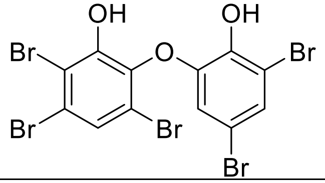	MRSA (ATCC 43300) (IC_{50} = 0.1 μM)<br>*E. coli* (ATCC 35218) (IC_{50} = 0 μM)<br>*P. aeruginosa* (ATCC 27853) (IC_{50} = 0 μM)<br>*M. intracellulare* (ATCC 23068) (IC_{50} = 0 μM)	*Lendenfeldia dendyi* collected in Papua New Guinea.	
DP017	2,3,4-tribromo-6-(3,5-dibromo-2-hydroxyphenoxy)phenol<br>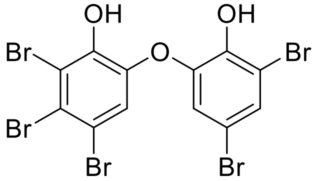	MRSA (ATCC 43300) (IC_{50} = 0.05 μM)<br>*E. coli* (ATCC 35218) (IC_{50} = 25.1 μM)<br>*P. aeruginosa* (ATCC 27853) (IC_{50} = 0 μM)<br>*M. intracellulare* (ATCC 23068) (IC_{50} = 0 μM)		
DP018	3,5-dibromo-2-(2,4-dibromophenoxy)phenol<br>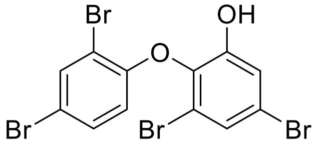	MRSA (ATCC 43300) (IC_{50} < 0.04 μM)<br>*E. coli* (ATCC 35218) (IC_{50} = 2.99 μM)<br>*P. aeruginosa* (ATCC 27853) (IC_{50} = 0 μM)<br>*M. intracellulare* (ATCC 23068) (IC_{50} = 0 μM)<br>*B. subtilis* (NBRC 13719) (MIC > 50 μM)	*Arenosclera* sp. collected in Phu Quoc, Vietnam.	[51,52]

		K. pneumoniae (NBRC 14940) (MIC > 50 μM)<br>*E. coli* (NBRC 102203) (MIC > 50 μM)<br>*S. aureus* (NBRC 100910) (MIC > 50 μM)		
DP018	3,5-dibromo-2-(2,4-dibromophenoxy)phenol<br>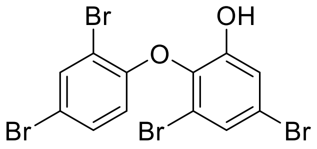	*Streptomyces* 85E (zone of inhibition 20 mm)<br>*Streptomyces* 85E (zone of inhibition 17 mm)<br>*Streptomyces* 85E (zone of inhibition 16 mm)<br>*Streptomyces* 85E (zone of inhibition 13 mm)	*Dysidea* sp. collected in Federated States of Micronesia.	[53]
DP019	Diorcinol L<br>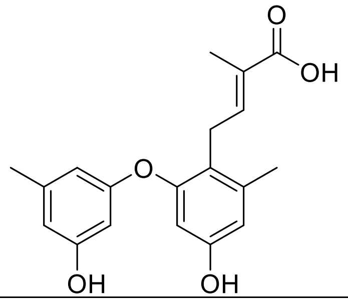	*R. solanacearum* (MIC = 8 μg/mL)<br>*E. coli* (MIC = 8 μg/mL)<br>*P. aeruginosa* (MIC = 16 μg/mL)<br>*B. subtilis* (MIC > 64 μg/mL)	*Aspergillus tennesseensis* OUCMB I 140430 isolated from a marine alga collected in Qingdao, China.	[54]
DP020	(R)-diorcinol B	*E. coli* (MIC = 4 μg/mL)<br>*B. subtilis* (MIC = 8 μg/mL)<br>*P. aeruginosa* (MIC = 16 μg/mL)<br>*R. solanacearum* (MIC = 16 μg/mL)		

	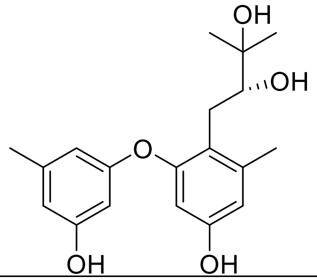			
DP021	(S)-diorcinol B 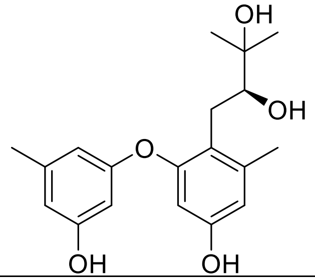	*P. aeruginosa* (MIC = 16 μg/mL)<br>*R. solanacearum* (MIC = 32 μg/mL)<br>*E. coli* (MIC > 64 μg/mL)<br>*B. subtilis* (MIC > 64 μg/mL)		
DP022	9-acetyldiorcinol B 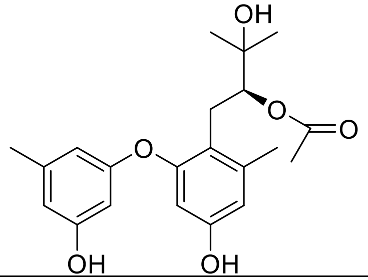	*R. solanacearum* (MIC = 8 μg/mL)<br>*B. subtilis* (MIC = 16 μg/mL)<br>*E. coli* (MIC = 16 μg/mL)<br>*P. aeruginosa* (MIC = 32 μg/mL)		
DP023	Diorcinol C	*B. subtilis* (MIC = 8 μg/mL)<br>*P. aeruginosa* (MIC = 32 μg/mL)<br>*E. coli* (MIC > 64 μg/mL)<br>*R. solanacearum* (MIC > 64 μg/mL)		

	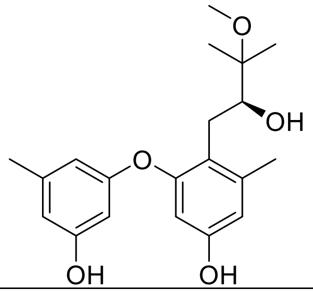			
DP024	Diorcinol E<br>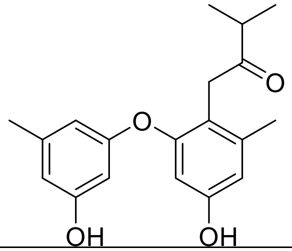	*B. subtilis* (MIC = 8 μg/mL)<br>*R. solanacearum* (MIC = 32 μg/mL)<br>*E. coli* (MIC = 64 μg/mL)<br>*P. aeruginosa* (MIC > 64 μg/mL)		
DP025	Diorcinol J<br>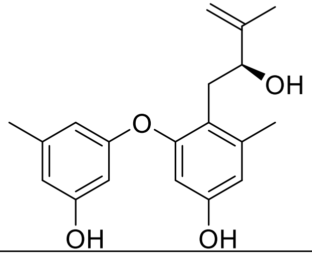	*B. subtilis* (MIC = 4 μg/mL)<br>*R. solanacearum* (MIC = 16 μg/mL)<br>*P. aeruginosa* (MIC = 64 μg/mL)<br>*E. coli* (MIC > 64 μg/mL)		
DP026	3-((2-(2-hydroxypropan-2-yl)-6-methyl-2,3-dihydrobenzofuran-4-yl)oxy)-5-methylphenol	*B. subtilis* (MIC = 2 μg/mL)<br>*P. aeruginosa* (MIC = 32 μg/mL)<br>*E. coli* (MIC = 64 μg/mL)<br>*R. solanacearum* (MIC > 64 μg/mL)		

	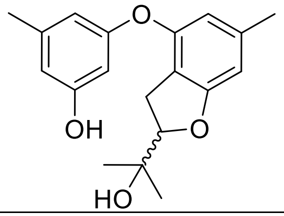			
DP027	Diorcinol K 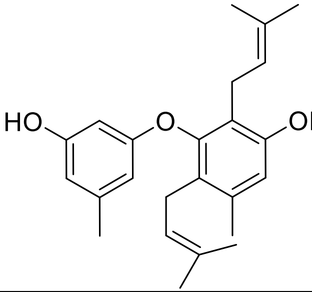	*S. aureus* (ATCC 6538) (MIC = 3.125 μg/mL)<br>MRSA (ATCC 700698) (MIC = 3.125 μg/mL)	*Aspergillus* sp. CUGB-F046 isolated from a marine sediment collected in Bohai Sea, China.	[55]
DP028	Diorcinol F 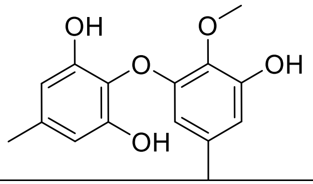	*S. aureus* (ATCC 6538) (MIC > 50 μg/mL)<br>MRSA (ATCC 700698) (MIC > 50 μg/mL)		
DP029	Tribromoiododiphenyl ether 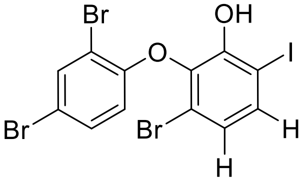	*B. subtilis* (NBRC 13719) (MIC = 3.1 μM)<br>*K. pneumoniae* (NBRC 14940) (MIC = 6.3 μM)<br>*S. aureus* (NBRC 100910) (MIC = 6.3 μM)<br>*E. coli* (NBRC 102203) (MIC > 50 μM)	*Arenosclera* sp. collected in Phu Quoc, Vietnam.	[52]
DP030	2-(2',4'-dibromophenoxy)-4,6-dibromophenol	*B. subtilis* (NBRC 13719) (MIC = 0.5 μM)		

	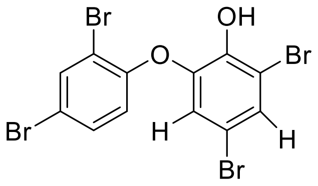	*S. aureus* (NBRC 100910) (MIC = 0.5 μM)<br>*K. pneumoniae* (NBRC 14940) (MIC = 3.1 μM)<br>*E. coli* (NBRC 102203) (MIC = 6.3 μM)		
DP031	2-(4'-bromophenoxy)-3,5-dibromophenol<br>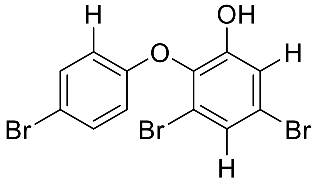	*S. aureus* (NBRC 100910) (MIC = 0.5 μM)<br>*B. subtilis* (NBRC 13719) (MIC = 0.8 μM)<br>*K. pneumoniae* (NBRC 14940) (MIC = 6.3 μM)<br>*E. coli* (NBRC 102203) (MIC = 12.5 μM)		
DP032	5-bromo-2-(2',4'-dibromophenoxy) phenol<br>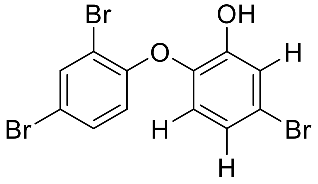	*B. subtilis* (NBRC 13719) (MIC = 12.5 μM)<br>*K. pneumoniae* (NBRC 14940) (MIC = 25 μM)<br>*S. aureus* (NBRC 100910) (MIC = 25 μM)<br>*E. coli* (NBRC 102203) (MIC > 50 μM)		
DP033	2-(2',4'-dibromophenoxy)-5,6-dibromo-3-chlorophenol<br>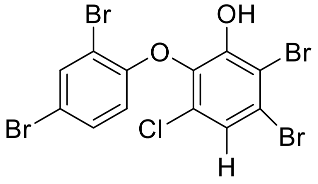	*B. subtilis* (NBRC 13719) (MIC = 0.5 μM)<br>*S. aureus* (NBRC 100910) (MIC = 1.6 μM)<br>*K. pneumoniae* (NBRC 14940) (MIC > 50 μM)<br>*E. coli* (NBRC 102203) (MIC > 50 μM)		

DP035	2-(2',4'-dibromophenoxy)-3,5,6-tribromophenol 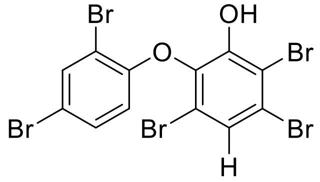	*B. subtilis* (NBRC 13719) (MIC = 0.5 μM)<br>*S. aureus* (NBRC 100910) (MIC = 0.8 μM)<br>*K. pneumoniae* (NBRC 14940) (MIC > 50 μM)<br>*E. coli* (NBRC 102203) (MIC > 50 μM)		
DP036	3,6-dibromo-2-(2',4'-dibromophenoxy) phenol 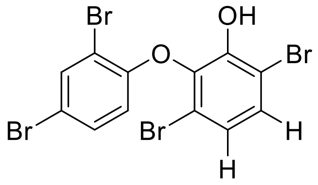	*B. subtilis* (NBRC 13719) (MIC = 3.1 μM)<br>*K. pneumoniae* (NBRC 14940) (MIC = 6.3 μM)<br>*S. aureus* (NBRC 100910) (MIC = 6.3 μM)<br>*E. coli* (NBRC 102203) (MIC > 50 μM)		
DP037	2-(2',4'-dibromophenoxy)-4,6-dibromoanisole 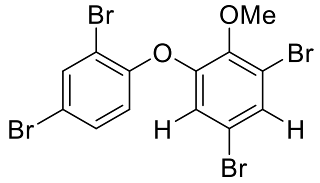	*B. subtilis* (NBRC 13719) (MIC > 50 μM)<br>*K. pneumoniae* (NBRC 14940) (MIC > 50 μM)<br>*S. aureus* (NBRC 100910) (MIC > 50 μM)<br>*E. coli* (NBRC 102203) (MIC > 50 μM)<br>*B. subtilis* (zone of inhibition 0 mm)<br>*B. subtilis* (zone of inhibition 0 mm)<br>*B. subtilis* (zone of inhibition 0 mm)	*Lamellodysidea herbacea* collected in Sangiang Island, West Java, Indonesia.	[52,56]

		B. subtilis (zone of inhibition 0 mm)		
DP038	2-(2',4'-dibromophenoxy)-3,4,5-tribromophenol 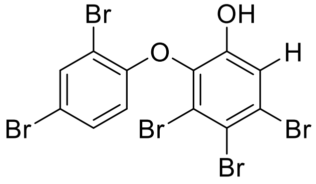	*L. monocytogenes* (MIC = 0.1 mg/L)<br>MRSA (MIC = 0.1 mg/L)<br>*C. jejuni* (ATCC 33291) (MIC = 5 mg/L)<br>*C. difficile* (ATCC 9689) (MIC = 10 mg/L)<br>*Salmonella* sp. (MIC = 10 mg/L)<br>MSSA (MIC = 0 mg/L)<br>*B. cereus* (MIC = 0 mg/L)<br>*S. pneumoniae* (ATCC 49136) (MIC = 0 mg/L)<br>*E. coli* (O157:H7) (MIC = 0 mg/L)<br>*Pseudomonas* sp. (MIC = 0 mg/L)<br>*K. pneumoniae* (ATCC 13883) (MIC = 0 mg/L)<br>*N. gonorrhoeae* (MIC = 0 mg/L)<br>*A. baumannii* (ATCC 19606) (MIC = 0 mg/L)<br>*P. aeruginosa* (MIC = 0 mg/L)	*Dysidea granulosa.*	[57]
DP039	2,5-dibromo-6-(3′,5′-dibromo-2′-hydroxyphenoxy)phenol 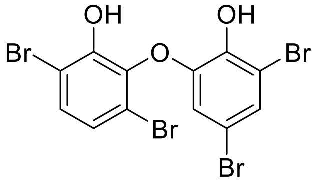	*B. subtilis* (zone of inhibition 7 mm)<br>*B. subtilis* (zone of inhibition 13 mm)<br>*B. subtilis* (zone of inhibition 20 mm)<br>*B. subtilis* (zone of inhibition 20 mm)	*Lamellodysidea herbacea* collected in Sangiang Island, West Java, Indonesia.	[56]
DP040	2,4,5-tribromo-6-(5′-bromo-2′-hydroxyphenoxy)phenol	*B. subtilis* (zone of inhibition 7 mm)		

	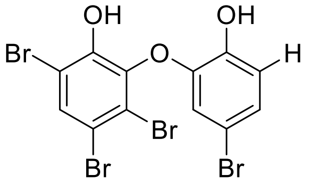	*B. subtilis* (zone of inhibition 8 mm)<br>*B. subtilis* (zone of inhibition 16 mm)<br>*B. subtilis* (zone of inhibition 17 mm)		
DP041	2,4,5-tribromo-6-(3′,5′- dibromo-2′-hydroxyphenoxy)anisole<br>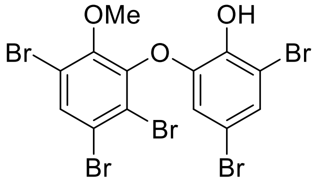	*B. subtilis* (zone of inhibition 6 mm)<br>*B. subtilis* (zone of inhibition 7 mm)<br>*B. subtilis* (zone of inhibition 10 mm)<br>*B. subtilis* (zone of inhibition 13 mm)		
DP042	2,3,4,5-tetrabromo-6-(3,5-dibromo-2-methoxyphenoxy)phenol<br>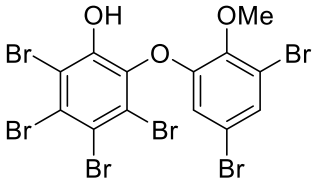	*B. subtilis* (zone of inhibition 10 mm)<br>*B. subtilis* (zone of inhibition 10 mm)<br>*B. subtilis* (zone of inhibition 13 mm)<br>*B. subtilis* (zone of inhibition 0 mm)		
DP043	3,4-dibromo-2-(5-bromo-2-hydroxyphenoxy)phenol<br>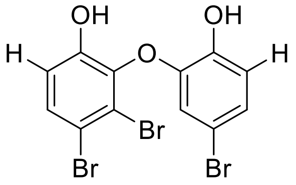	*B. subtilis* (zone of inhibition 7 mm)<br>*B. subtilis* (zone of inhibition 13 mm)<br>*B. subtilis* (zone of inhibition 16 mm)<br>*B. subtilis* (zone of inhibition 18 mm)		

DP045	1,5-dibromo-2-(3,5-dibromo-2-methoxyphenoxy)-3-methoxybenzene 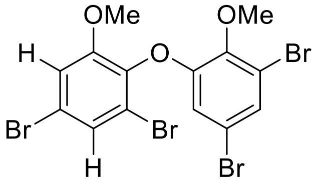	*B. subtilis* (zone of inhibition 0 mm) *B. subtilis* (zone of inhibition 0 mm) *B. subtilis* (zone of inhibition 0 mm) *B. subtilis* (zone of inhibition 0 mm)		
DP046	1,2,3-tribromo-4-(3,5-dibromo-2-methoxyphenoxy)-5-methoxybenzene 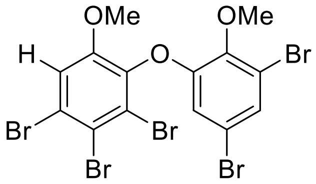	*B. subtilis* (zone of inhibition 0 mm) *B. subtilis* (zone of inhibition 0 mm) *B. subtilis* (zone of inhibition 0 mm) *B. subtilis* (zone of inhibition 0 mm)		
DP047	2,4-dibromo-6-(3,4,6-tribromo-2-methoxyphenoxy)phenol 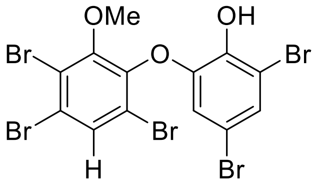	*B. subtilis* (zone of inhibition 6 mm) *B. subtilis* (zone of inhibition 9 mm) *B. subtilis* (zone of inhibition 9 mm) *B. subtilis* (zone of inhibition 9 mm)		
DP048	2,4-dibromo-6-(2,3,4-tribromo-6-methoxyphenoxy)phenol	*B. subtilis* (zone of inhibition 0 mm) *B. subtilis* (zone of inhibition 0 mm) *B. subtilis* (zone of inhibition 0 mm)		

	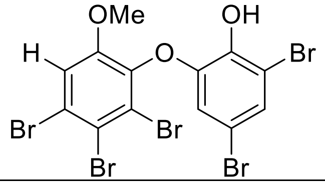	*B. subtilis* (zone of inhibition 0 mm)		
DP049	3,4,5-tribromo-2-(3,5-dibromo-2-methoxyphenoxy)phenol 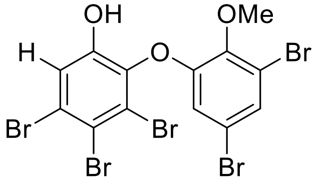	*B. subtilis* (zone of inhibition 6 mm)<br>*B. subtilis* (zone of inhibition 9 mm)<br>*B. subtilis* (zone of inhibition 10 mm)<br>*B. subtilis* (zone of inhibition 10 mm)		
DP050	1,2,5-tribromo-4-(3,5-dibromo-2-methoxyphenoxy)-3-methoxybenzene 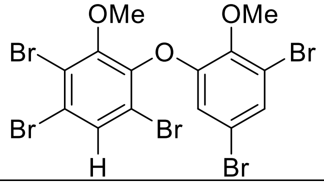	*B. subtilis* (zone of inhibition 0 mm)	Semi-synthetic compound obtained from DP013.	
DP051	1,4-dibromo-2-(3,5-dibromo-2-methoxyphenoxy)-3-methoxybenzene 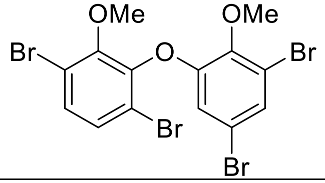		Semi-synthetic compound obtained from DP039.	
DP052	1,2,5-tribromo-3-(5-bromo-2-methoxyphenoxy)-4-methoxybenzene		Semi-synthetic compound obtained from DP040.	

	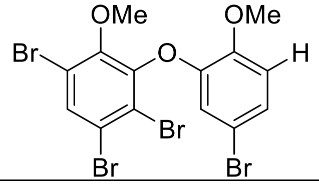			
DP053	1,2,3,4-tetrabromo-5-(3,5-dibromo-2-methoxyphenoxy)-6-methoxybenzene 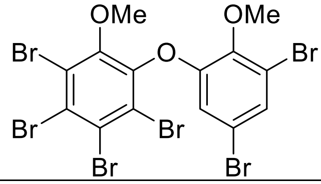		Semi-synthetic compound obtained from DP014.	
DP054	2,4-dibromo-6-(2,3,4,5-tetrabromo-6-methoxyphenoxy)phenol 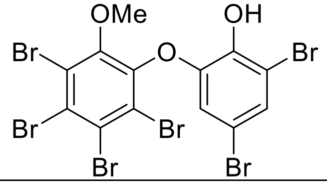		Semi-synthetic compound obtained from DP014.	
DP055	1,2-dibromo-3-(5-bromo-2-methoxyphenoxy)-4-methoxybenzene hydrobromide 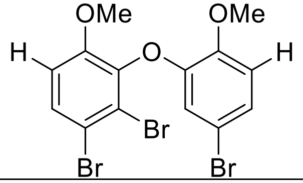		Semi-synthetic compound obtained from DP043.	
DP056	3,4,5-tribromo-2-(3,5-dibromo-2-hydroxyphenoxy)phenol	*B. subtilis* (zone of inhibition 7 mm)<br>*B. subtilis* (zone of inhibition 10 mm)	Semi-synthetic compound obtained from DP014.	

	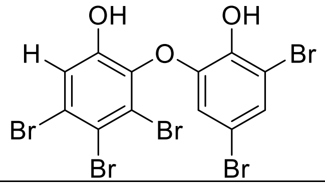	*B. subtilis* (zone of inhibition 13 mm)<br>*B. subtilis* (zone of inhibition 16 mm)		
DP057	2,3,4-tribromo-6-(3-bromo-2-hydroxyphenoxy)phenol<br>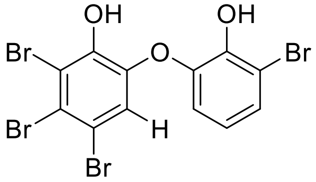	*B. subtilis* (zone of inhibition 8 mm)<br>*B. subtilis* (zone of inhibition 13 mm)<br>*B. subtilis* (zone of inhibition 0 mm)<br>*B. subtilis* (zone of inhibition 0 mm)	Semi-synthetic compound obtained from DP014.	
DP058	3,5-dibromo-2-(3,5-dibromo-2-hydroxyphenoxy)phenol<br>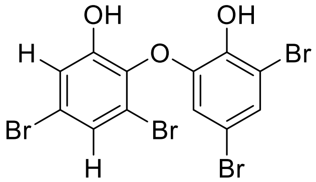	*B. subtilis* (zone of inhibition 16 mm)<br>*B. subtilis* (zone of inhibition 18 mm)<br>*B. subtilis* (zone of inhibition 0 mm)<br>*B. subtilis* (zone of inhibition 0 mm)	Semi-synthetic compound obtained from DP014.	
DP059	2,4-dibromo-6-(4-bromo-2-hydroxyphenoxy)phenol<br>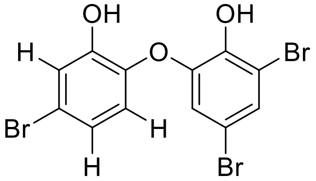	*B. subtilis* (zone of inhibition 7 mm)<br>*B. subtilis* (zone of inhibition 13 mm)<br>*B. subtilis* (zone of inhibition 0 mm)<br>*B. subtilis* (zone of inhibition 0 mm)	Semi-synthetic compound obtained from DP014.	
DP061	2,4-dibromo-6-(5-bromo-2-hydroxyphenoxy)phenol	*B. subtilis* (zone of inhibition 6 mm)	Semi-synthetic compound obtained from DP014.	

	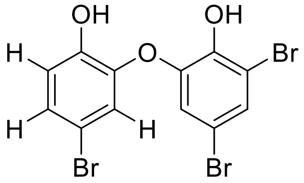	*B. subtilis* (zone of inhibition 6 mm)<br>*B. subtilis* (zone of inhibition 8 mm)<br>*B. subtilis* (zone of inhibition 11 mm)		
DP062	2,3,4,5-tetrabromo-6-(2-bromophenoxy)phenol<br>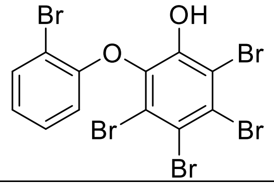	*B. subtilis* (168) (MIC = 0.2 μg/mL)	*Dysidea herbacea* collected in West Sumatra, Indonesia.	[58]
DP063	3,4,5-tribromo-2-(2'-bromophenoxy)phenol<br>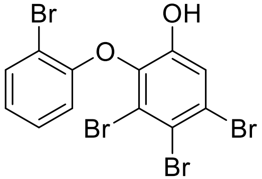	*B. subtilis* (168) (MIC = 6.25 μg/mL)		
DP064	3,5,6-tribromo-2-(2'-bromophenoxy)-phenol<br>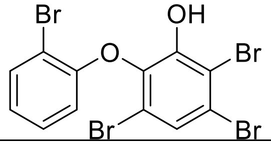	Mixture of DP064 + DP065 (3:2)<br>*B. subtilis* (168) (MIC = 1.56 μg/mL)		
DP065	3,4,6-tribromo-2-(2'-bromophenoxy)phenol<br>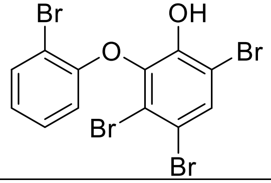			

DP066	3,5,6-Tribromo-1-(2'-bromophenoxy)-2-benzene methyl ether 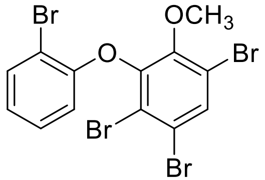	*B. subtilis* (168) (MIC = 104 μg/mL)		
DP067	2,3,4,5-tetrabromo-6-(2,4-dibromophenoxy)phenol 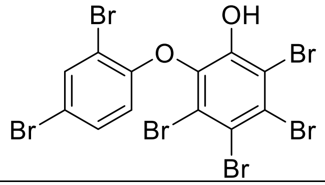	*B. subtilis* (168) (MIC = 3.13 μg/mL)		
DP068	2,4-dibromo-6-(2,4-dibromophenoxy)phenol 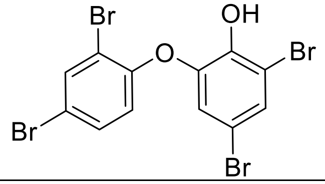	*B. subtilis* (168) (MIC = 25 μg/mL)		
DP069	Δ1′,3′ -1′-dehydroxypenicillide 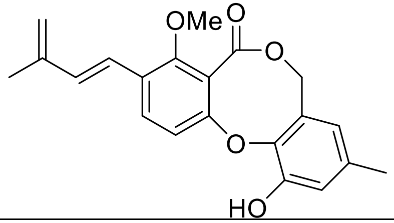	*M. luteus* (MIC = 0 μg/mL)<br>*E. coli* (MIC = 0 μg/mL)	*Penicillium* sp. MA-37 isolated from the mangrove plant *Bruguiera gymnorrhiza* collected in Hainan Island, China.	[59]
DP070	7-O-acetylsecopenicillide C	*M. luteus* (MIC = 16 μg/mL)<br>*E. coli* (MIC = 64 μg/mL)		

	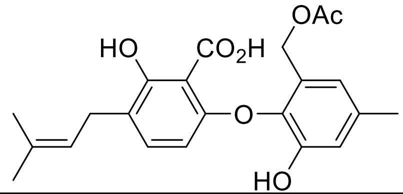			
DP071	Hydroxytenellic acid B 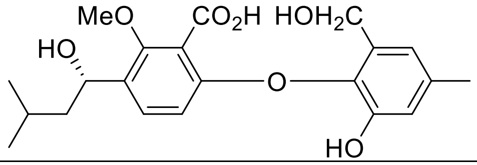	*M. luteus* (MIC = 0 μg/mL)<br>*E. coli* (MIC = 0 μg/mL)		
DP072	6-[2-hydroxy-6-(hydroxymethyl)-4-methylphenoxy]-2-methoxy-3-(1-methoxy-3-methylbutyl)benzoic acid 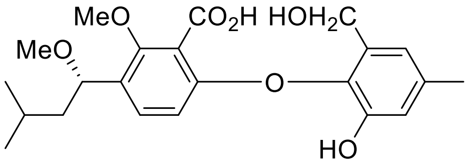	*M. luteus* (MIC = 32 μg/mL)<br>*E. coli* (MIC = 256 μg/mL)		
DP074	Tenellic acid B 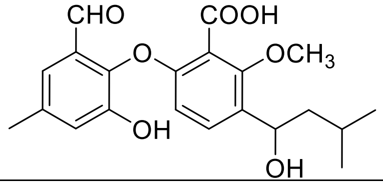	B. subtilis (ATCC 6051) (zone of inhibition 9 mm)	*Dendrospora tenella* collected from foam in Wood Point, New Brunswick, Canada.	[60]
DP075	3,4,5,6-tetrabromo-2-(2',4'-dibromophenoxy)phenol	*B. subtilis* (MIC = 0.5 μg/mL)<br>*S. aureus* (MIC = 2.1 μg/mL)<br>*E. coli* (MIC > 66.7 μg/mL)<br>*A. baumannii* (MIC > 66.7 μg/mL)	*Lamellodysidea herbacea* collected in Kepulauan Seribu, Indonesia.	[61]

	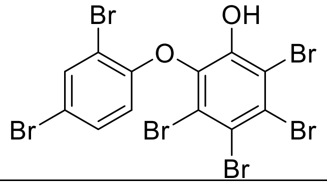			
DP076	Cordyol E 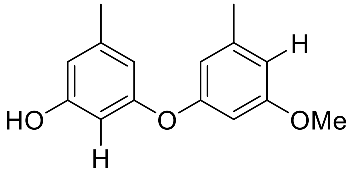	*M. tuberculosis* (MIC > 100 μM) *E. faecium* (ATCC 35667; ATCC 700221) (MIC > 100 μM) *E. faecalis* (ATCC 29212; ATCC 51299) (MIC > 100 μM) *A. baumannii* (ATCCBAA 1605) (MIC > 100 μM) *S. aureus* (ATCC 25923; ATCC 700699) (MIC > 100 μM)	*Aspergillus* sp. isolated from the marine sponge *Chondrilla nucula* collected in the Aegean Sea, Turkey.	[62]
DP077	Cordyol C 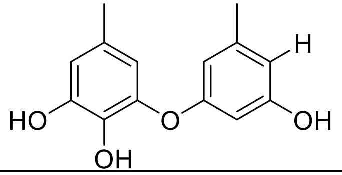			
DP078	4-methoxycarbonyldiorcinol 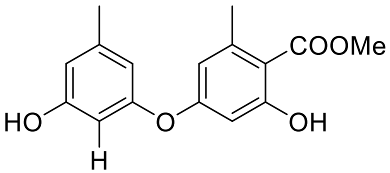			
DP079	Pseudopithoether A 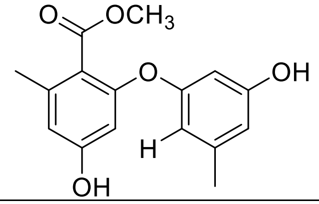	*P. aeruginosa* (ATCC 27853) (MIC > 200 μg/mL) *E. coli* (ATCC 25922) (MIC > 200 μg/mL) *A. baumannii* (NPRC 005) (MIC > 200 μg/mL)	*Pseudopithomyces maydicus* PSU-AMF350 isolated from the marine bryozoan *Schizoporella* sp. collected in the Phuket Province, Thailand.	[63]

DP080	Pseudopithoether B<br>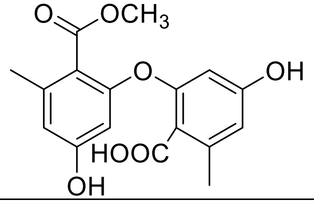	*A. baumannii* (NPRC 007) (MIC > 200 μg/mL)<br>S. aureus (MIC > 200 μg/mL)<br>MRSA (MIC > 200 μg/mL)		
DP081	Methyl diorcinol-4-carboxylate<br>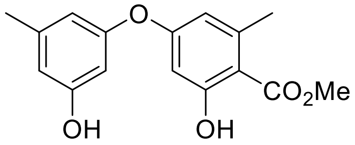	*P. aeruginosa* (ATCC 10145) (MIC = 13.9 μM)<br>*S. aureus* (ATCC 25923) (MIC = 55.6 μM)<br>*C. perfringens* (ATCC 13048) (MIC = 55.6 μM)<br>*S. aureus* (ATCC 6538) (MIC > 200 μM)<br>*E. coli* (ATCC 11775) (MIC > 200 μM)<br>*B. subtilis* (ATCC 6051) (MIC > 128 μM)	*Aspergillus versicolor* OUCMDZ-2738 isolated from *Enteromorpha prolifera* collected in Shilaoren beach, Qingdao, China.	[64]
DP082	Diorcinolic acid<br>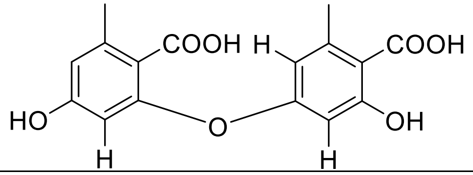	*S. aureus* (SG 503) (MIC > 40 μg/mL)<br>*S. aureus* (SG 511) (MIC > 40 μg/mL)<br>*S. iniae* (FP3187) (MIC > 40 μg/mL)<br>*V. ichthyoenteri* (Vi0917-1) (MIC > 40 μg/mL)<br>*V. ichthyoenteri* (Vi099-7) (MIC > 40 μg/mL)	*Aspergillus sydowii* J05B-7F-4 isolated from the marine sponge *Stelletta* sp.	[65]
DP083	4-carboxydiorcinal<br>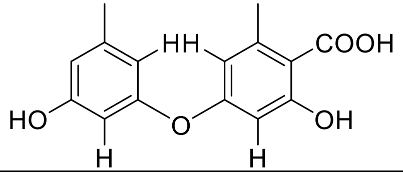			

DP084	Violaceol II 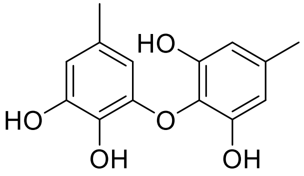	*S. aureus* (SG 503) (MIC = 40 μg/mL) *S. aureus* (SG 511) (MIC = 40 μg/mL) *S. iniae* (FP3187) (MIC = 40 μg/mL) *V. ichthyoenteri* (Vi0917-1) (MIC > 40 μg/mL) *V. ichthyoenteri* (Vi099-7) (MIC > 40 μg/mL)		
DP085	Violaceol I 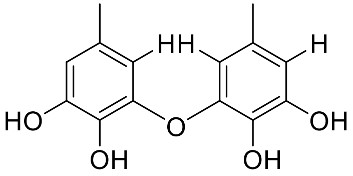	*S. aureus* (SG 511) (MIC = 40 μg/mL) *V. ichthyoenteri* (Vi0917-1) (MIC > 40 μg/mL) *V. ichthyoenteri* (Vi099-7) (MIC > 40 μg/mL) *S. aureus* (SG 503) (MIC > 40 μg/mL) *S. iniae* (FP3187) (MIC > 40 μg/mL)		
DP086	Peniciaculin A 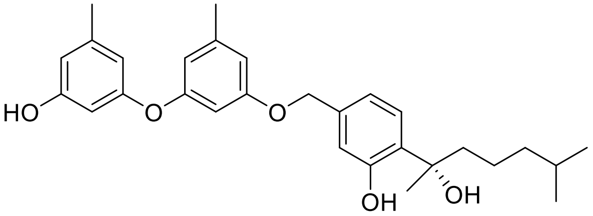	*M. luteus* (QDIO-3) (MIC = 1 μg/mL) *V. alginolyticus* (QDIO-5) (MIC = 2 μg/mL) *P. aeruginosa* (QDIO-4) (MIC = 32 μg/mL) *E. coli* (EMBLC-1) (MIC > 32 μg/mL)	*Penicillium aculeatum* SD-321 isolated from a marine sediment collected in the South China Sea.	[66]

		A. hydrophilia (QDIO-1) (MIC > 32 μg/mL)<br>*E. tarda* (QDIO-2) (MIC > 32 μg/mL)<br>*V.anguillarum* (QDIO-6) (MIC > 32 μg/mL)<br>*V. harveyi* (QDIO-7) (MIC > 32 μg/mL)<br>*V. parahemolyticus* (QDIO-8) (MIC > 32 μg/mL)<br>*S. aureus* (EMBLC-2) (MIC > 32 μg/mL)		
DP087	Crossbyanol A<br>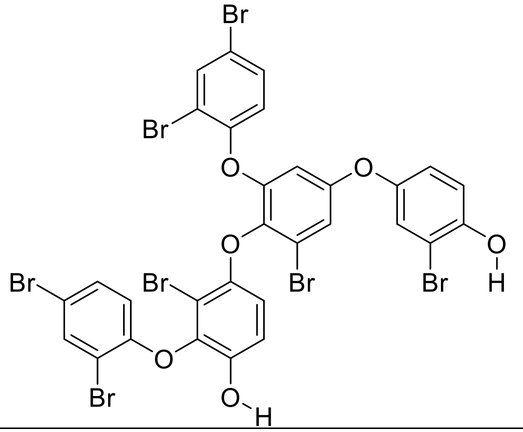	MRSA (MIC = 0 μg/mL)	*Leptolyngbya crossbyana* isolated from a coral collected in Honaunau reef, Hawai'i.	[67]
DP088	Crossbyanol B	MRSA (MIC = 2.0-3.9 μg/mL)		

	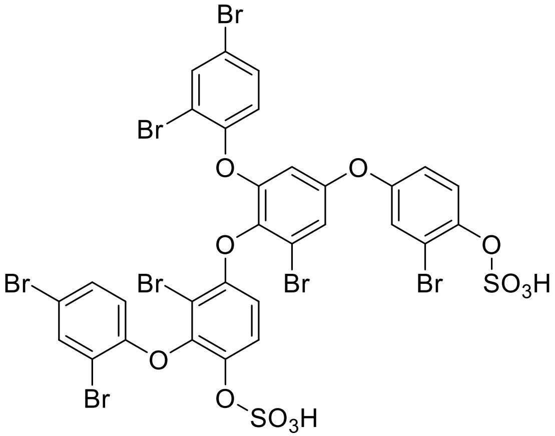			
DP089	Crossbyanol C<br>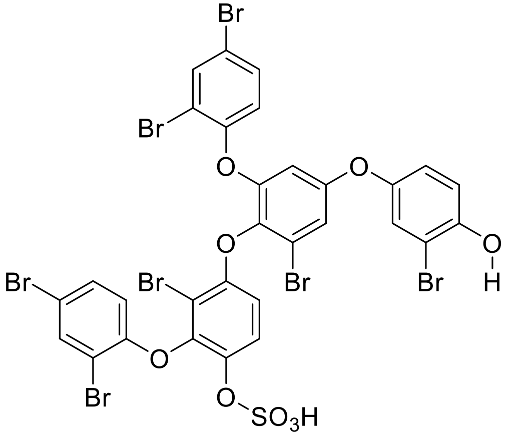	MRSA (MIC = 15.6-31.3 μg/mL)		
DP090	Crossbyanol D	MRSA (MIC = 7.81-15.6 μg/mL)		

	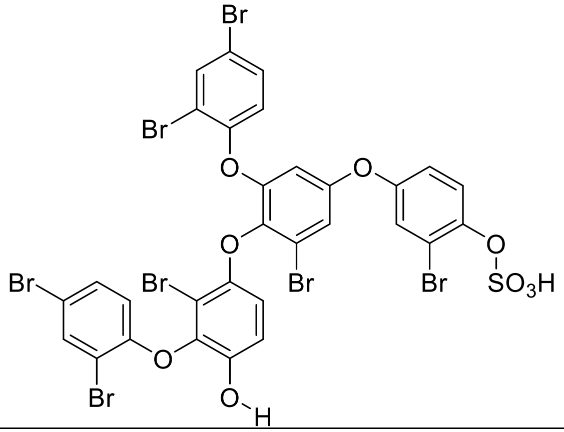			
DP092	4′-chloroasterric acid 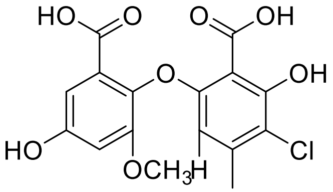	*S. aureus* (ATCC 25923) (MIC = 12.5 μg/mL)<br>*S. aureus* (ATCC 33591) (MIC = 12.5 μg/mL)<br>*S. aureus* (ATCC 29213) (MIC = 25 μg/mL)<br>*S. aureus* (ATCC 43300) (MIC > 100 μg/mL)<br>*E. faecalis* (ATCC 51299) (MIC > 100 μg/mL)<br>*E. faecium* (ATCC 35667) (MIC > 100 μg/mL)<br>*V. parahemolyticus* (ATCC 17802) (MIC > 100 μg/mL)	*Aspergillus flavipes* DL-11 isolated from a marine sediment collected in Dalian, Liaoning province, China.	[44]
DP093	Methyl chloroasterrate	*S. aureus* (ATCC 33591) (MIC = 50 μg/mL)<br>*S. aureus* (ATCC 29213) (MIC = 50 μg/mL)		

	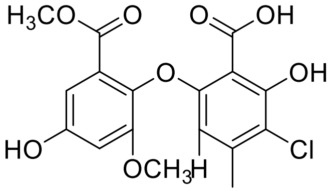	*S. aureus* (ATCC 25923) (MIC > 100 μg/mL)<br>*S. aureus* (ATCC 43300) (MIC > 100 μg/mL)<br>*E. faecalis* (ATCC 51299) (MIC > 100 μg/mL)<br>*E. faecium* (ATCC 35667) (MIC > 100 μg/mL)<br>*V. parahemolyticus* (ATCC 17802) (MIC > 100 μg/mL)		
DP094	Penicillither<br>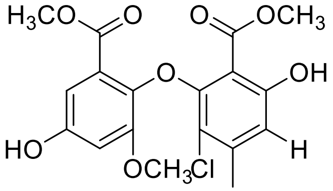	*S. aureus* (ATCC 43300) (MIC = 12.5 μg/mL)<br>*S. aureus* (ATCC 29213) (MIC = 50 μg/mL)<br>*E. faecium* (ATCC 35667) (MIC = 50 μg/mL)<br>*E. faecalis* (ATCC 51299) (MIC > 100 μg/mL)<br>*V. parahemolyticus* (ATCC 17802) (MIC > 100 μg/mL)		
DP095	Iizukine A<br>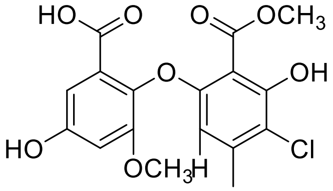	*E. faecium* (ATCC 35667) (MIC = 3.13 μg/mL)<br>*S. aureus* (ATCC 43300) (MIC = 12.5 μg/mL)<br>*S. aureus* (ATCC 29213) (MIC = 50 μg/mL)<br>*E. faecalis* (ATCC 51299) (MIC > 100 μg/mL)<br>*V. parahemolyticus* (ATCC 17802) (MIC > 100 μg/mL)		

DP096	Asterric acid 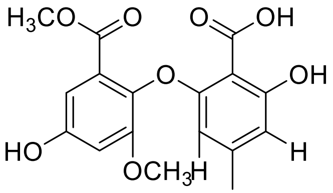	E. faecium (ATCC 35667) (MIC = 3.13 μg/mL)<br>*S. aureus* (ATCC 29213) (MIC > 100 μg/mL)<br>*S. aureus* (ATCC 43300) (MIC > 100 μg/mL)<br>*E. faecalis* (ATCC 51299) (MIC > 100 μg/mL)<br>*V. parahemolyticus* (ATCC 17802) (MIC > 100 μg/mL)		
DP097	Monomethylosoic acid 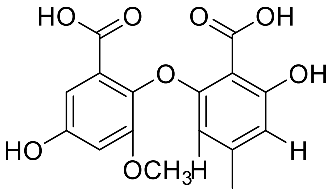	*S. aureus* (ATCC 33591) (MIC = 12.5 μg/mL)<br>*S. aureus* (ATCC 29213) (MIC = 50 μg/mL)<br>*S. aureus* (ATCC 25923) (MIC > 100 μg/mL)<br>*S. aureus* (ATCC 43300) (MIC > 100 μg/mL)<br>*E. faecalis* (ATCC 51299) (MIC > 100 μg/mL)<br>*E. faecium* (ATCC 35667) (MIC > 100 μg/mL)<br>*V. parahemolyticus* (ATCC 17802) (MIC > 100 μg/mL)		
DP098	Butyl 2,4-dichloroasterrate 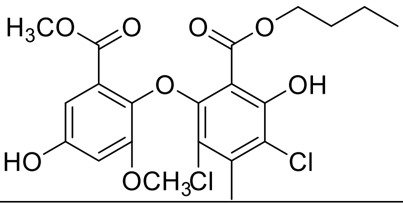	*S. aureus* (ATCC 43300) (MIC = 6.25 μg/mL)<br>*E. faecium* (ATCC 35667) (MIC = 6.25 μg/mL)<br>*S. aureus* (ATCC 29213) (MIC = 12.5 μg/mL)		

		E. faecalis (ATCC 51299) (MIC = 12.5 µg/mL)<br>*V. parahemolyticus* (ATCC 17802) (MIC > 100 µg/mL)		
DP099	2,4-dichloroasterric acid<br>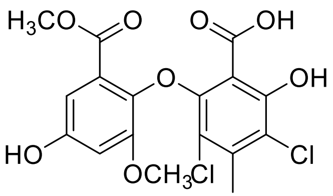 	*S. aureus* (ATCC 43300) (MIC = 12.5 µg/mL)<br>*E. faecium* (ATCC 35667) (MIC = 50 µg/mL)<br>*S. aureus* (ATCC 29213) (MIC > 100 µg/mL)<br>*E. faecalis* (ATCC 51299) (MIC > 100 µg/mL)<br>*V. parahemolyticus* (ATCC 17802) (MIC > 100 µg/mL)		
DP100	Methyl dichloroasterrate<br>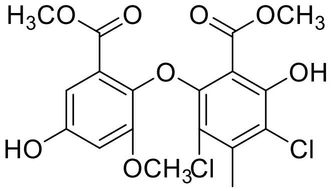 	*S. aureus* (ATCC 43300) (MIC = 3.13 µg/mL)<br>*E. faecium* (ATCC 35667) (MIC = 25 µg/mL)<br>*S. aureus* (ATCC 29213) (MIC > 100 µg/mL)<br>*E. faecalis* (ATCC 51299) (MIC > 100 µg/mL)<br>*V. parahemolyticus* (ATCC 17802) (MIC > 100 µg/mL)		
DP101	Geodin hydrate<br>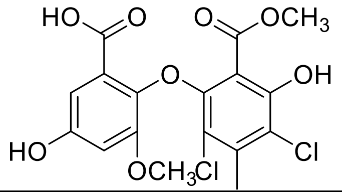 	*S. aureus* (ATCC 33591) (MIC = 3.13 µg/mL)<br>*S. aureus* (ATCC 29213) (MIC = 25 µg/mL)<br>*S. aureus* (ATCC 25923) (MIC = 50 µg/mL)		

		S. aureus (ATCC 43300) (MIC > 100 μg/mL)<br>*E. faecalis* (ATCC 51299) (MIC > 100 μg/mL)<br>*E. faecium* (ATCC 35667) (MIC > 100 μg/mL)<br>*V. parahemolyticus* (ATCC 17802) (MIC > 100 μg/mL)		
XT018	Yicathin C<br>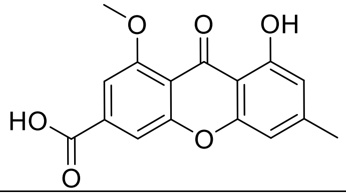	*E. coli* (zone of inhibition 12 mm)<br>*S. aureus* (zone of inhibition 7.5 mm)	*Aspergillus wentii* pt-1 isolated from the marine alga *Gymnogongrus flabelliformis* collected in the Coast of Pingtan Island, China.	[68]
XT019	Yicathin B<br>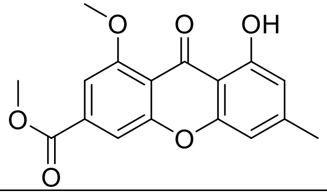	*E. coli* (zone of inhibition 9 mm)		
XT021	Aloesone<br>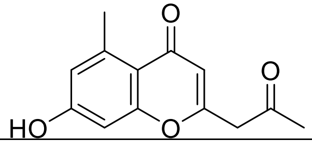	*S. aureus* (MIC > 200 μg/mL)<br>MRSA (MIC = 200 μg/mL)		
XT022	2-(2'S-Hydroxypropyl)-5-methyl-7-hydroxychromone	*S. aureus* (MIC > 200 μg/mL)<br>MRSA (MIC > 200 μg/mL)		

	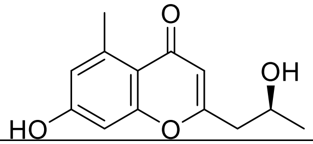			
XT023	Oxisterigmatocystin I 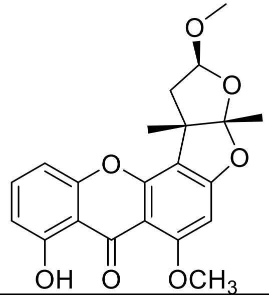	*S. aureus* (ATCC 25923) (MIC > 192 μg/mL) *V. parahemolyticus* (ATCC 17802) (MIC > 192 μg/mL)	*Aspergillus* sp. F40 isolated from the marine sponge *Callyspongia* sp. collected in Xuwen County, Guangdong Province, China.	[29]
XT024	Oxisterigmatocystin C 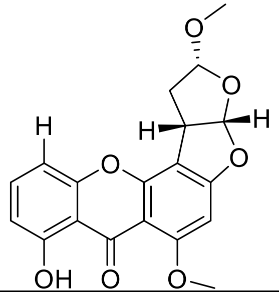	*V. parahemolyticus* (ATCC 17802) (MIC > 192 μg/mL) *S. aureus* (ATCC 25923) (MIC = 48 μg/mL)	*Aspergillus versicolor* CXCTD-06-6a isolated from the marine sponge *Callyspongia* sp. collected in the Pacific Ocean.	[29,69]
XT025	Aspergilluone A 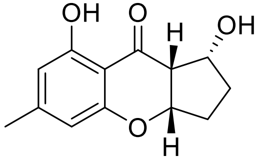	*P. aeruginosa* (ATCC 10145) (MIC = 0 μg/mL) *M. tuberculosis* (H37Rv ATCC 27294) (MIC = 32 μg/mL) *S. aureus* (ATCC 6538) (MIC = 64 μg/mL) *B. subtilis* (JCM 1465) (MIC = 128 μg/mL)	*Aspergillus* sp. LS57 isolated from the marine sponge *Haliclona* sp. collected in Lingshui, Hainan Province, China.	[70]

		E. coli (JCM 1649) (MIC = 128 μg/mL)		
XT026	methyl (4aS,7aR,8S,15S)-8-acetoxy-1,4,12,14,16-pentahydroxy-18-methyl-13,17-dioxo-2,3,4,7,8,13,15,17-octahydro-4aH-7a,15-ethenonaphtho[2',3':4,5]cyclohepta[1,2-b]xanthene-4a-carboxylate<br>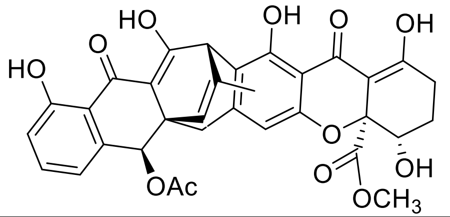 	MRSA (IC_{50} = 0.19 μM)<br>*S. epidermis* (IC_{50} = 0.2 μM)<br>*P. acnes* (IC_{50} = 11 μM)		
XT027	Engyodontochone A<br>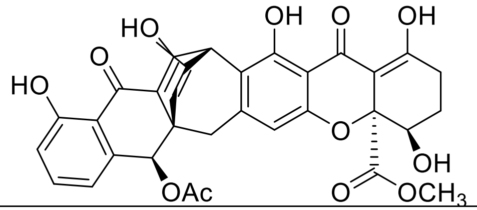 	MRSA (IC_{50} = 0.17 μM)<br>*S. epidermis* (IC_{50} = 0.19 μM)<br>*P. acnes* (IC_{50} = 13.8 μM)	*Engyodontium album* LF069 isolated from the marine sponge *Cacospinga scalaris* collected in Limski Fjord, Croatia.	[71]
XT028	methyl (1R,8aR,9S,16S,17aR)-9-acetoxy-1,4,6,13,15-pentahydroxy-18-methyl-5,14-dioxo-1,2,3,5,8,9,14,16-octahydro-17aH-8a,16-ethenonaphtho[2',3':5,6]cyclohepta[1,2-c]xanthene-17a-carboxylate	*S. epidermis* (IC_{50} = 0.21 μM)<br>MRSA (IC_{50} = 0.25 μM)<br>*P. acnes* (IC_{50} = 14.1 μM)		

	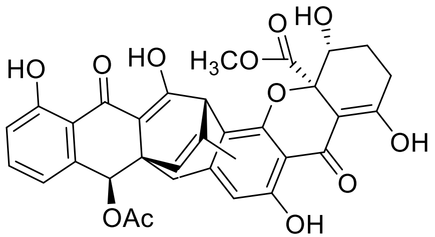			
XT029	Engyodontochone B 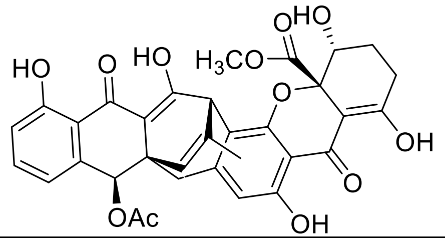	*S. epidermis* (IC_{50} = 0.22 μM)<br>MRSA (IC_{50} = 0.24 μM)<br>*P. acnes* (IC_{50} = 11.7 μM)		
XT030	Engyodontochone C 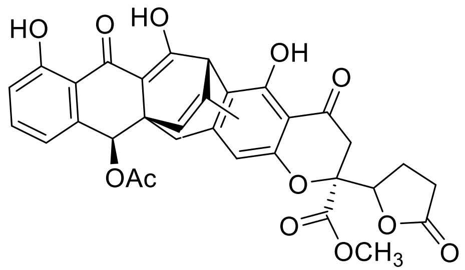	*S. epidermis* (IC_{50} = 1.8 μM)<br>MRSA (IC_{50} = 2.39 μM)<br>*P. acnes* (IC_{50} > 100 μM)		
XT032	Engyodontochone E 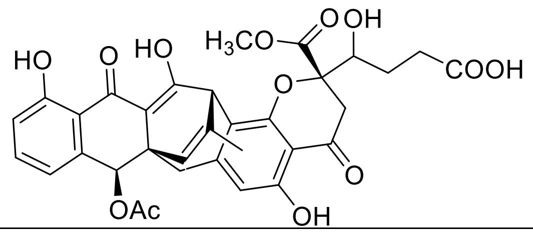	MRSA (IC_{50} = 6.74 μM)<br>*S. epidermis* (IC_{50} = 6.77 μM)<br>*P. acnes* (IC_{50} > 100 μM)		

XT033	Engyodontochone F 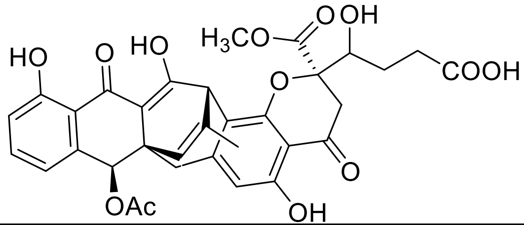	MRSA (IC_{50} = 3.13 μM)<br>*S. epidermis* (IC_{50} = 3.41 μM)<br>*P. acnes* (IC_{50} > 100 μM)		
XT034	Penicillixanthone A 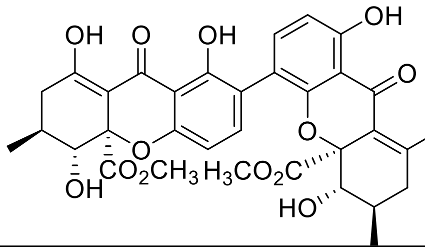	*M. luteus* (UST950701-006) (MIC = 24.4 μg/mL)<br>*E. coli* (JVC1228) (MIC = 24.4 μg/mL)<br>*B. subtilis* (MIC = 24.4 μg/mL)<br>*P. nigrifaciens* (UST010620-005) (MIC = 97.5 μg/mL)	*Penicillium sp.* SCSGAF 0023 isolated from a marine gorgonian coral *Dichotella gemmacea* collected in the South China Sea, Hainan Province, China.	[72]
XT035	Pseudopithoxanthone 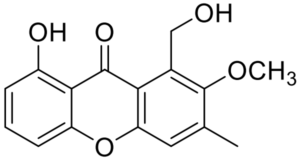	*P. aeruginosa* (ATCC 27853) (MIC > 200 μg/mL)<br>*E. coli* (ATCC 25922) (MIC > 200 μg/mL)<br>*A. baumannii* (NPRC005) (MIC > 200 μg/mL)<br>*A. baumannii* (NPRC007) (MIC > 200 μg/mL)<br>*S. aureus* (MIC > 200 μg/mL)<br>MRSA (MIC > 200 μg/mL)	*Pseudopithomyces maydicus* PSU-AMF350 isolated from the bryozoan *Schizoporella* sp. collected in Phuket Province, Thailand.	[63]
XT036	13-O-acetylsydowinin B	*S. aureus* (SG 503) (MIC > 40 μg/mL)<br>*S. aureus* (SG 511) (MIC > 40 μg/mL)<br>*S. iniae* (FP3187) (MIC > 40 μg/mL)	*Aspergillus sydowii* isolated from the marine sponge *Stelletta* sp.	[65]

	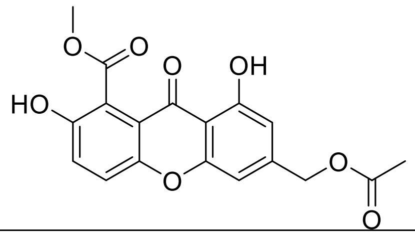	*V. ichthyoenteri* (Vi0917-1) (MIC > 40 μg/mL)<br>*V. ichthyoenteri* (Vi099-7) (MIC > 40 μg/mL)		
XT037	Methyl-(2-chloro-1,6-dihydroxy-3-methylxanthone)-8-carboxylate<br>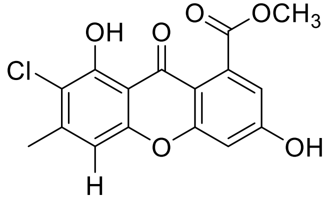	*S. aureus* (ATCC 25923) (MIC = 3.13 μg/mL)<br>*S. aureus* (ATCC 33591) (MIC = 3.13 μg/mL)<br>*S. aureus* (ATCC 29213) (MIC = 6.25 μg/mL)<br>*S. aureus* (ATCC 43300) (MIC = 6.25 μg/mL)<br>*E. faecalis* (ATCC 51299) (MIC > 100 μg/mL)<br>*E. faecium* (ATCC 35667) (MIC > 100 μg/mL)<br>*V. parahemolyticus* (ATCC 17802) (MIC > 100 μg/mL)	*Aspergillus flavipes* DL-11 isolated from a marine sediment collected in Dalian, Liaoning province, China.	[44]
XT038	Methyl-(4-chloro-1,6-dihydroxy-3-methylxanthone)-8-carboxylate<br>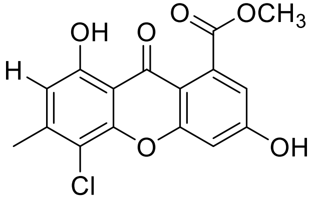	*S. aureus* (ATCC 33591) (MIC = 1.56 μg/mL)<br>*S. aureus* (ATCC 25923) (MIC = 3.13 μg/mL)<br>*S. aureus* (ATCC 29213) (MIC = 3.13 μg/mL)<br>*S. aureus* (ATCC 43300) (MIC = 3.13 μg/mL)<br>*E. faecalis* (ATCC 51299) (MIC = 25 μg/mL)		

		E. faecium (ATCC 35667) (MIC = 50 μg/mL)<br>*V. parahemolyticus* (ATCC 17802) (MIC > 100 μg/mL)		
XT039	1,4,7-Trihydroxy-6-methylxanthone<br>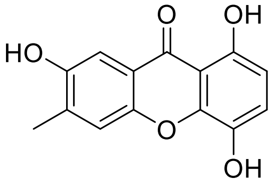 	*E. coli* (MIC = 32 μg/mL)<br>*P. aeruginosa* (MIC = 32 μg/mL)<br>*V. alginolyticus* (MIC = 32 μg/mL)<br>*V. harveyi* (MIC = 32 μg/mL)<br>*V. parahaemolyticus* (MIC = 32 μg/mL)<br>*S. aureus* (MIC > 64 μg/mL)	*Talaromyces islandicus* EN-501 isolated from the marine alga *Laurencia okamurai* collected in Qingdao, China.	[45]
XT040	1,4,5-Trihydroxy-2-methylxanthone<br>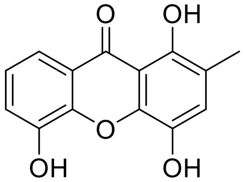 	*E. coli* (MIC = 4 μg/mL)<br>*P. aeruginosa* (MIC = 4 μg/mL)<br>*V. alginolyticus* (MIC = 4 μg/mL)<br>*V. parahaemolyticus* (MIC =4 μg/mL)<br>*S. aureus* (MIC = 8 μg/mL)<br>*V. harveyi* (MIC = 8 μg/mL)		
XT081	Sterigmatocystin<br>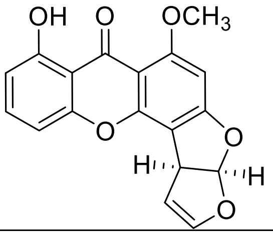 	*S. aureus* (zone of inhibition 9 mm)<br>*B. subtilis* (MIC = 12.5 μM)<br>*P. aeruginosa* (MIC = 12.5 μM)<br>*E. coli* (MIC = 12.5 μM)<br>*S. aureus* (MIC > 25 μM)	*Aspergillus versicolor* HBU-2017-7 collected in the Bohai Sea, China.	[1,2]
XT170	Variecoxanthone A	*S. aureus* (zone of inhibition 0 mm)	*Aspergillus versicolor* isolated from a marine clam collected in the East China Sea, China.	[1]

	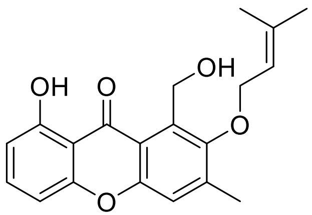			
XT241	5-methoxysterigmatocystin 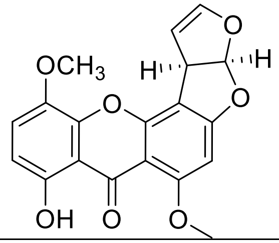	*B. subtilis* (MIC = 6.13 μM)<br>*S. aureus* (MIC = 6.13 μM)<br>*P. aeruginosa* (MIC = 12.5 μM)<br>*E. coli* (MIC > 25 μM)	*Aspergillus versicolor* HBU-2017-7 collected at the Bohai Sea.	[2]
XT886	Arugosin C 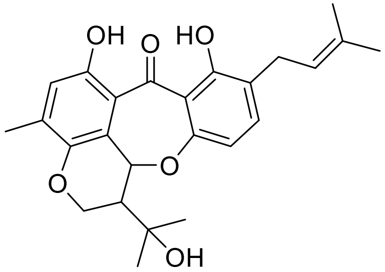	*B. megaterium* (zone of inhibition 0 mm)<br>*B. cereus* (zone of inhibition 0 mm)<br>*B. subtilis* (zone of inhibition 0 mm)<br>*S. aureus* (zone of inhibition 0 mm)<br>*E. cloacae* (zone of inhibition 0 mm)<br>*K. pneumoniae* (zone of inhibition 0 mm)<br>*E. coli* (zone of inhibition 0 mm)	*Aspergillus versicolor* isolated from the marine alga *Halimeda opuntia* collected in the Red Sea, South Sinai, Egypt.	[22]

MIC: Minimum inhibitory concentration, IC_{50}: Half maximal inhibitory concentration; MRSA: Methicillin-resistant *Staphylococcus aureus*, VRE: Vancomycin-resistant *Enterococcus*, MRCNS: Methicillin-resistant coagulase-negative staphylococci; MDR: Multidrug-resistant bacteria.

Table S2. Molecular descriptors calculated for the studied marine polyketides.

ID	MW	Fraction Csp3	Rotatable bonds	TPSA	iLOGP	XLOGP3	WLOGP	MLOGP	Silicos-IT Log P	Mean Log P	ESOL Log S	Ali Log S	Mean Log S
AQ001	754,78	0,33	14	217,35	6,11	8,35	7,15	0,32	8,15	6,31	-9,18	-12,78	-10,98
AQ002	726,72	0,3	12	239,35	4,9	8,38	5,84	-0,02	7,01	5,43	-9,17	-13,27	-11,22
AQ003	386,4	0,33	6	124,29	2,95	4,02	3,23	0,43	3,79	3,00	-4,69	-6,33	-5,51
AQ004	372,37	0,3	5	135,29	2,54	4,03	2,57	0,21	3,24	2,58	-4,69	-6,57	-5,63
AQ005	354,35	0,2	4	115,06	2,4	4,52	3,38	0,93	3,83	3,09	-4,96	-6,66	-5,81
AQ006	326,3	0,17	3	111,9	1,77	4,12	2,52	1,02	3,24	2,53	-4,63	-6,18	-5,405
AQ007	368,34	0,2	5	117,97	1,72	3,68	2,74	1,42	3,73	2,67	-4,44	-5,85	-5,145
AQ008	396,39	0,27	7	95,97	3,19	3,78	3,14	1,86	4,84	3,32	-4,52	-5,49	-5,005
AQ009	296,32	0,22	3	63,6	2,92	4,37	3,13	1,65	4,32	3,25	-4,64	-5,42	-5,03
AQ010	338,35	0,25	5	69,67	3,25	3,93	3,35	2,02	4,8	3,45	-4,44	-5,09	-4,765
AQ011	282,29	0,18	2	74,6	2,31	4,05	2,83	1,42	3,79	2,87	-4,43	-5,32	-4,875
AQ012	310,34	0,26	4	52,6	3	4,15	3,43	1,89	4,86	3,46	-4,5	-4,96	-4,73
AQ014	338,31	0,5	1	144,52	1,74	-0,95	-1	-1,82	0,05	-0,50	-1,46	-1,6	-1,53
AQ015	356,32	0,56	1	167,91	1,09	-2,35	-2,36	-2,51	-1,03	-1,53	-0,68	-0,64	-0,66
AQ016	340,33	0,56	1	147,68	1,37	-1,58	-1,47	-1,73	-0,54	-0,85	-1,07	-1,01	-1,04
AQ017	372,75	0,5	1	144,52	1,37	-0,44	-0,64	-1,57	0,68	-0,21	-1,99	-2,13	-2,06
AQ018	338,31	0,56	1	139,98	1,74	-1,35	-1,31	-1,73	0,2	-0,57	-1,21	-1,09	-1,15
AQ019	304,29	0,38	1	104,06	2,17	0,88	0,98	-0,31	1,81	1,09	-2,42	-2,65	-2,535
AQ020	320,29	0,38	1	124,29	1,79	-0,23	-0,05	-1,11	0,92	0,21	-1,81	-1,92	-1,865

AQ021	254,24	0,07	0	74,6	1,21	3,07	2,18	0,92	3,04	2,10	-3,82	-4,3	-4,06
AQ022	254,24	0,07	0	74,6	2,22	3,53	2,18	0,92	3,04	2,35	-4,11	-4,78	-4,445
AQ023	400,42	0,36	7	113,29	3,25	4,69	3,18	0,65	4,34	3,27	-5,12	-6,8	-5,96
AQ024	388,37	0,3	6	133,52	2,21	1,69	1,35	-0,58	2,94	1,49	-3,23	-4,11	-3,67
AQ025	396,39	0,36	2	91,29	3,37	3,63	3,21	1,06	4,02	3,14	-4,76	-5,24	-5
AQ026	382,36	0,33	1	102,29	3,15	3,3	2,91	0,84	3,48	2,82	-4,54	-5,12	-4,83
AQ027	412,39	0,36	2	111,52	3,05	2,65	2,18	0,27	3,13	2,30	-4,23	-4,64	-4,435
AQ028	368,34	0,3	2	91,29	2,86	2,95	2,41	0,61	3,29	2,42	-4,18	-4,53	-4,355
AQ029	366,32	0,2	2	91,29	2,92	3,25	2,53	0,54	2,88	2,44	-4,36	-4,84	-4,6
AQ030	276,28	0,4	0	97,99	1,9	0,37	0,13	0,07	0,41	0,56	-2,01	-1,99	-2
AQ031	290,27	0,33	0	115,06	1,51	-0,02	-0,14	-0,83	0,89	0,21	-1,84	-1,95	-1,895
AQ032	270,24	0,07	0	94,83	1,8	2,72	1,89	0,36	2,55	1,87	-3,67	-4,37	-4,02
AQ042	284,26	0,12	1	83,83	2,32	3,04	2,19	0,61	3,07	2,24	-3,87	-4,47	-4,17
AQ043	432,38	0,33	5	162,98	2,05	0,61	-0,67	-1,77	1,04	0,12	-2,86	-3,61	-3,235
AQ050	402,35	0,25	6	150,59	1,41	1,59	0,7	-0,62	2,5	1,05	-3,25	-4,36	-3,805
AQ051	394,37	0,23	3	125,04	2,31	2,04	2,12	-0,14	4,15	2,14	-3,78	-4,29	-4,035
AQ052	410,37	0,23	3	145,27	2,03	0,9	1,09	-0,93	3,38	1,30	-3,15	-3,54	-3,345
AQ053	376,36	0,23	1	97,11	3,02	3,14	3,11	0,66	5,18	3,05	-4,51	-4,85	-4,68
AQ054	324,33	0,21	3	91,67	2,4	4,14	3,16	0,98	4,24	3,01	-4,63	-5,77	-5,2
AQ055	532,56	0,5	2	207,12	2,48	-0,94	0,15	-1,65	1,48	0,33	-2,54	-2,93	-2,735
AQ056	514,54	0,42	2	186,89	2,45	-0,1	0,96	-0,96	1,96	0,93	-2,96	-3,37	-3,165
AQ057	434,44	0,28	1	124,29	3,48	3,34	2,58	0,56	3,42	2,71	-4,94	-5,63	-5,285
AQ058	890,99	0,7	8	282,73	3,76	0,65	1,85	-2,38	1,41	1,24	-5,32	-6,16	-5,74
AQ059	328,32	0,22	3	104,06	2,23	2,69	1,81	0,28	3,06	1,98	-3,74	-4,53	-4,135

AQ060	342,3	0,17	4	110,13	2,35	2,37	1,79	0,22	2,94	1,94	-3,55	-4,32	-3,935
AQ063	290,27	0,33	1	115,06	1,32	-0,16	-0,08	-0,83	1,18	0,25	-1,68	-1,8	-1,74
AQ064	274,27	0,33	0	94,83	1,68	1,09	0,89	-0,02	1,79	1,05	-2,45	-2,67	-2,56
AQ065	314,29	0,18	2	115,06	1,95	2,36	1,5	0,04	2,53	1,65	-3,53	-4,42	-3,975
AQ066	238,24	0,07	0	54,37	2,25	3,88	2,48	1,51	3,53	2,69	-4,25	-4,72	-4,485
AQ067	286,24	0,07	1	115,06	1,54	1,47	0,92	-0,45	1,94	1,08	-2,9	-3,49	-3,195
AQ068	298,29	0,18	3	72,83	2,3	2,7	2,13	0,99	3,17	2,29	-3,6	-3,88	-3,74
AQ069	282,25	0,06	2	80,67	1,19	2,5	1,99	0,27	3,25	1,86	-3,46	-3,84	-3,65
AQ070	240,21	0	0	74,6	1,64	3,16	1,87	0,67	2,55	1,96	-3,81	-4,4	-4,105
AQ071	386,4	0,33	6	124,29	2,95	4,02	3,23	0,43	3,79	3,00	-4,69	-6,33	-5,51
AQ072	368,34	0,3	0	113,29	2,45	2,98	2,6	0,61	2,95	2,42	-4,33	-5,02	-4,675
AQ073	384,34	0,3	0	133,52	2,16	2	1,58	-0,17	2,05	1,59	-3,8	-4,43	-4,115
AQ074	340,33	0,56	1	147,68	1,12	-0,88	-0,88	-1,46	-0,52	-0,53	-1,51	-1,74	-1,625
AQ075	324,33	0,56	1	127,45	2,08	0,09	0,15	-0,67	0,37	0,42	-2,03	-2,32	-2,175
AQ076	344,27	0,12	2	141,36	1,52	2,76	1,5	-1,08	2,06	1,38	-3,94	-5,38	-4,66
AQ077	304,3	0,05	0	74,6	2,61	4,32	3,33	1,7	4,08	3,23	-4,96	-5,6	-5,28
AQ078	304,3	0,05	0	74,6	2,66	4,87	3,33	1,7	4,08	3,33	-5,31	-6,17	-5,74
AQ079	340,33	0,26	0	115,06	2,14	1,82	1,28	0,24	2,6	1,61	-3,45	-3,86	-3,655
AQ080	296,27	0,12	1	91,67	1,95	2,76	2,38	0,51	3,46	2,24	-3,75	-4,34	-4,045
AQ081	328,32	0,22	3	104,06	2	2,69	1,81	0,28	3,06	1,94	-3,74	-4,53	-4,135
AQ082	284,26	0,12	1	83,83	2,02	3,04	2,19	0,61	3,07	2,19	-3,87	-4,47	-4,17
AQ088	336,29	0,05	0	115,06	1,84	3,61	2,75	0,59	3,11	2,44	-4,67	-5,71	-5,19
AQ089	336,29	0,05	0	115,06	1,76	3,61	2,75	0,59	3,11	2,43	-4,67	-5,71	-5,19
AQ092	272,25	0,13	0	86,99	2,03	3,18	2,24	1,79	2,48	2,33	-3,98	-4,68	-4,33

AQ094	300,22	0	1	132,13	0,93	1,88	1,28	-0,24	1,48	1,10	-3,22	-4,28	-3,75
AQ095	338,35	0,59	2	116,45	2,07	0,63	0,8	-0,43	0,9	0,85	-2,39	-2,65	-2,52
AQ098	324,33	0,56	1	127,45	2,08	0,09	0,15	-0,67	0,37	0,42	-2,03	-2,32	-2,175
AQ099	340,33	0,56	1	147,68	1,12	-0,88	-0,88	-1,46	-0,52	-0,53	-1,51	-1,74	-1,625
AQ104	300,26	0,12	1	104,06	2,13	3,24	1,9	0,07	2,6	1,97	-4,08	-5,1	-4,59
AQ105	342,3	0,17	3	110,13	2,45	2,8	2,12	0,49	3,07	2,17	-3,88	-4,77	-4,325
AQ106	342,3	0,17	3	110,13	2,32	2,8	2,12	0,49	3,07	2,15	-3,88	-4,77	-4,325
AQ107	342,3	0,17	3	110,13	2,39	3,35	2,12	0,49	3,07	2,26	-4,23	-5,34	-4,785
AQ108	384,34	0,2	5	116,2	2,72	2,35	2,34	0,9	3,56	2,37	-3,69	-4,43	-4,06
AQ109	384,34	0,2	5	116,2	2,87	2,9	2,34	0,9	3,56	2,48	-4,04	-5	-4,52
AQ110	384,34	0,2	5	116,2	2,91	2,9	2,34	0,9	3,56	2,49	-4,04	-5	-4,52
AQ111	426,37	0,23	7	122,27	3,32	2,46	2,55	1,3	4,08	2,71	-3,86	-4,67	-4,265
AQ112	284,26	0,12	1	83,83	2,43	4,52	2,19	0,61	3,07	2,50	-4,81	-6	-5,405
AQ113	326,3	0,17	3	89,9	2,79	3,15	2,41	1,02	3,54	2,55	-4,02	-4,71	-4,365
AQ114	340,28	0,22	0	113,29	2,03	2,3	1,8	0,16	2,23	1,72	-3,75	-4,32	-4,035
AQ123	344,32	0,22	4	135,29	1,78	1,39	1,04	-0,52	2,31	1,17	-2,94	-3,83	-3,385
AQ125	286,24	0,07	1	104,06	1,82	2,32	1,59	-0,18	2,09	1,54	-3,43	-4,14	-3,785
AQ126	574,49	0,33	2	208,12	2,2	0,4	1,41	-1,87	1,31	0,81	-3,73	-4,34	-4,035
AQ127	296,27	0,12	1	91,67	1,96	2,76	2,38	0,51	3,46	2,24	-3,75	-4,34	-4,045
AQ128	270,24	0,07	0	94,83	2,22	3,27	1,89	0,36	2,55	2,03	-4,02	-4,94	-4,48
AQ129	296,27	0,12	1	91,67	1,95	2,76	2,38	0,51	3,46	2,24	-3,75	-4,34	-4,045
AQ130	340,28	0,22	0	113,29	1,53	2,3	1,8	0,16	2,23	1,64	-3,75	-4,32	-4,035
AQ131	370,35	0,3	3	102,29	2,58	2,82	2,42	0,21	3,44	2,37	-4,04	-4,63	-4,335
AQ132	284,27	0,2	0	89	1,77	-0,69	0,16	0,61	3,42	0,96	-1,66	-0,7	-1,18

AQ133	342,35	0,39	0	101,53	1,83	-1,92	-0,2	0,23	2	0,33	-1,11	0,31	-0,4
AQ134	356,37	0,42	1	101,53	2,04	-1,39	0,19	0,46	2,39	0,68	-1,45	-0,24	-0,845
AQ136	268,26	0,12	1	63,6	2,4	3,4	2,48	1,17	3,56	2,58	-4,02	-4,41	-4,215
AQ137	506,46	0,07	1	149,2	3,37	5,85	4,34	0,92	5,8	4,10	-7,07	-8,75	-7,91
AQ140	552,53	0,28	0	150,59	3,53	4,21	3,75	1,13	4,57	3,51	-6,24	-7,08	-6,66
AQ141	568,53	0,28	0	170,82	3,14	3,16	2,87	0,36	4,08	2,76	-5,67	-6,42	-6,045
AQ142	566,55	0,3	1	139,59	3,48	3,99	4,06	1,32	5,13	3,69	-6,12	-6,62	-6,37
AQ143	582,55	0,3	1	159,82	3,49	2,93	3,17	0,55	4,63	3,01	-5,54	-5,95	-5,745
AQ144	534,51	0,22	0	130,36	3,78	4,89	4,56	1,84	5,06	4,13	-6,57	-7,36	-6,965
AQ145	550,51	0,22	0	150,59	3,49	3,81	3,67	1,06	4,56	3,40	-5,98	-6,67	-6,325
AQ146	550,51	0,22	1	150,59	3,33	3,64	3,53	1,06	4,45	3,28	-5,81	-6,49	-6,15
AQ147	550,51	0,22	1	150,59	3,33	3,64	3,53	1,06	4,45	3,28	-5,81	-6,49	-6,15
AQ148	904,99	0,65	9	208,88	5,72	4,82	5,35	-0,66	5,24	4,36	-8,03	-8,94	-8,485
AQ149	678,72	0,57	5	175,12	4,38	3,1	3,32	-0,43	4,13	3,02	-5,85	-6,45	-6,15
AQ150	598,59	0,48	7	197,12	3,35	2,46	2,01	-0,85	2,9	2,03	-4,85	-6,24	-5,545
AQ151	710,72	0,57	5	215,58	3,53	0,55	1,27	-1,93	1,57	1,10	-4,35	-4,65	-4,5
AQ152	504,48	0,64	1	191,05	1	-1,81	-1,1	-2,46	0,86	-0,71	-1,88	-1,69	-1,785
AQ153	465,48	0,17	6	166,3	2,52	3,78	3,02	0,91	3,82	2,84	-5,07	-6,97	-6,02
AQ154	300,26	0,12	1	104,06	2,04	3,24	1,9	0,07	2,6	1,96	-4,08	-5,1	-4,59
AQ155	284,26	0,12	1	83,83	2,45	3,04	2,19	0,61	3,07	2,26	-3,87	-4,47	-4,17
AQ156	364,73	0,18	2	135,29	1,97	1,88	1,32	-0,25	2,4	1,49	-3,51	-4,34	-3,925
XT026	616,57	0,33	4	197,12	3,49	3,27	3,13	-0,02	2,83	2,69	-5,66	-7,08	-6,37
XT027	642,65	0,39	6	176,89	4,45	5,03	5,18	1,28	4,67	4,35	-6,79	-8,49	-7,64
XT028	616,57	0,33	4	197,12	3,67	3,27	3,13	-0,02	2,83	2,72	-5,66	-7,08	-6,37

XT029	616,57	0,33	4	197,12	3,86	3,27	3,13	-0,02	2,83	2,75	-5,66	-7,08	-6,37
XT030	616,57	0,36	5	182,96	3,42	3,03	3,25	0,79	3,75	2,97	-5,44	-6,54	-5,99
XT032	634,58	0,36	8	214,19	3,4	2,25	2,77	0,04	3,23	2,46	-4,86	-6,38	-5,62
XT033	634,58	0,36	8	214,19	2,43	2,25	2,77	0,04	3,23	2,30	-4,86	-6,38	-5,62
BZ001	288,25	0,07	3	115,06	1,2	3,19	2,04	1,02	1,85	1,89	-3,86	-5,28	-4,57
BZ002	409,26	0,25	2	86,99	2,98	5,93	4,9	2,8	5,49	4,53	-6,31	-7,53	-6,92
BZ004	366,75	0,18	5	113,29	1,97	3,57	2,79	1,49	3,12	2,62	-4,39	-5,63	-5,01
BZ005	401,19	0,18	5	113,29	1,93	4,19	3,44	1,72	3,78	3,08	-4,98	-6,28	-5,63
BZ008	260,24	0,07	2	97,99	1,36	2,76	2,05	0,84	1,93	1,83	-3,53	-4,47	-4
BZ009	274,27	0,13	3	86,99	1,83	3,09	2,35	1,1	2,46	2,20	-3,73	-4,58	-4,155
BZ010	374,34	0,21	7	119,36	2,77	3,05	2,36	1,4	2,97	2,48	-3,95	-5,22	-4,585
BZ011	416,38	0,24	9	125,43	3,93	3,15	2,58	1,81	3,49	2,92	-4,11	-5,45	-4,78
BZ012	332,3	0,18	5	113,29	2,21	2,94	2,14	0,98	2,46	2,14	-3,79	-4,98	-4,385
BZ013	348,3	0,18	6	133,52	2,26	1,69	1,17	0,2	1,87	1,42	-3,02	-4,11	-3,565
BZ014	439,29	0,24	6	104,06	3,14	6,28	4,98	2,52	5,78	4,61	-6,43	-8,25	-7,34
BZ015	380,78	0,22	6	102,29	2,29	3,89	3,09	1,72	3,66	2,96	-4,6	-5,74	-5,17
BZ016	346,33	0,22	6	102,29	2,72	3,27	2,44	1,22	3	2,52	-4,01	-5,09	-4,55
DP001	430,45	0,35	10	119,36	3,3	3,83	4,34	2,02	4,41	3,76	-4,55	-6,03	-5,29
DP002	416,46	0,39	10	102,29	3,18	4,16	4,81	2,07	4,85	4,03	-4,68	-6,02	-5,35
DP003	362,33	0,22	7	111,52	2,2	2,78	2,99	1,51	2,56	2,51	-3,72	-4,78	-4,25
DP004	298,38	0,26	4	49,69	3,24	5,4	5,02	3,74	4,73	4,52	-5,23	-6,2	-5,715
DP005	366,49	0,33	6	49,69	4,15	7,32	6,52	4,76	6,55	5,97	-6,66	-8,19	-7,425
DP006	298,38	0,26	4	49,69	3,43	5,4	5,02	3,74	4,73	4,56	-5,23	-6,2	-5,715
DP007	493,42	0,32	12	183,64	3,38	4,11	2,87	0,55	1,05	2,47	-4,95	-7,67	-6,31

DP008	479,39	0,29	11	194,64	2,64	3,78	2,78	0,33	0,49	2,13	-4,73	-7,56	-6,145
DP009	465,36	0,25	10	194,64	2,13	3,71	2,53	0,11	0,08	1,85	-4,67	-7,49	-6,08
DP010	393,3	0,18	7	168,34	2,46	3,39	2,6	0,18	-0,09	1,86	-4,27	-6,6	-5,435
DP011	372,41	0,29	7	96,22	3,14	3,84	4,1	2,7	4,16	3,72	-4,44	-5,56	-5
DP012	446,49	0,42	11	111,52	3,85	4,02	4,52	2,57	4,67	4,10	-4,69	-6,06	-5,375
DP013	610,71	0,08	3	38,69	4,08	6,52	7,01	5,5	5,94	6,01	-7,96	-7,13	-7,545
DP014	675,58	0	2	49,69	3,62	6,89	7,47	5,88	6,12	6,24	-8,66	-7,74	-8,2
DP015	759,66	0,12	6	61,83	4,46	6,83	7,9	6,52	7,06	6,78	-8,79	-7,94	-8,365
DP016	596,69	0	2	49,69	3,58	6,2	6,7	5,28	5,42	5,65	-7,76	-7,03	-7,395
DP017	596,69	0	2	49,69	3,56	6,2	6,7	5,28	5,42	5,64	-7,76	-7,03	-7,395
DP018	501,79	0	2	29,46	3,69	5,79	6,23	5,28	5,21	5,41	-6,96	-6,18	-6,57
DP019	328,36	0,21	5	86,99	2,45	4,09	4,08	2,8	3,68	3,53	-4,49	-5,62	-5,055
DP020	332,39	0,37	5	90,15	2,46	3,05	3,18	2,14	3,37	2,90	-3,86	-4,61	-4,235
DP021	332,39	0,37	5	90,15	2,53	3,05	3,18	2,14	3,37	2,91	-3,86	-4,61	-4,235
DP022	374,43	0,38	7	96,22	2,54	3,62	3,75	2,51	3,88	3,34	-4,31	-5,33	-4,82
DP023	346,42	0,4	6	79,15	3,01	3,58	3,84	2,37	3,92	3,43	-4,2	-4,93	-4,565
DP024	314,38	0,32	5	66,76	2,67	4,13	4,27	2,89	4,45	3,78	-4,45	-5,24	-4,845
DP025	314,38	0,26	5	69,92	2,82	4,35	3,99	2,89	4,13	3,69	-4,59	-5,53	-5,06
DP026	314,38	0,37	3	58,92	3,28	3,83	3,88	2,58	3,96	3,57	-4,39	-4,76	-4,575
DP027	366,49	0,33	6	49,69	4,21	7,32	6,52	4,76	6,55	5,98	-6,66	-8,19	-7,425
DP028	276,28	0,2	3	79,15	2,89	3,09	3,22	1,72	2,58	2,79	-3,75	-4,42	-4,085
DP029	548,79	0	2	29,46	3,41	5,82	6,08	5,39	5,5	5,38	-7,27	-6,21	-6,74
DP030	501,79	0	2	29,46	3,63	5,79	6,23	5,28	5,21	5,40	-6,96	-6,18	-6,57
DP031	422,89	0	2	29,46	3,38	5,17	5,47	4,65	4,54	4,78	-6,11	-5,53	-5,82

DP032	422,89	0	2	29,46	3,33	4,95	5,47	4,65	4,54	4,74	-5,97	-5,31	-5,64
DP033	536,24	0	2	29,46	3,58	6,49	6,89	5,78	5,86	5,91	-7,59	-6,9	-7,245
DP035	580,69	0	2	29,46	3,87	6,48	7	5,89	5,89	6,02	-7,86	-6,89	-7,375
DP036	501,79	0	2	29,46	3,65	5,86	6,23	5,28	5,21	5,41	-7	-6,25	-6,625
DP037	515,82	0,08	3	18,46	3,97	6,12	6,54	5,51	5,72	5,73	-7,16	-6,29	-6,725
DP038	580,69	0	2	29,46	3,87	6,48	7	5,89	5,89	6,02	-7,86	-6,89	-7,375
DP039	517,79	0	2	49,69	3,37	5,51	5,94	4,66	4,74	5,03	-6,86	-6,31	-6,585
DP040	517,79	0	2	49,69	3,05	5,51	5,94	4,66	4,74	4,97	-6,86	-6,31	-6,585
DP041	610,71	0,08	3	38,69	3,93	6,52	7,01	5,5	5,94	5,98	-7,96	-7,13	-7,545
DP042	689,61	0,08	3	38,69	4,22	7,21	7,77	6,1	6,63	6,62	-8,86	-7,85	-8,355
DP043	438,89	0	2	49,69	2,76	4,65	5,18	4,04	4,06	4,31	-5,85	-5,42	-5,635
DP045	545,84	0,14	4	27,69	4,13	6,16	6,55	5,12	5,77	5,71	-7,26	-6,52	-6,89
DP046	624,74	0,14	4	27,69	4,34	6,85	7,31	5,72	6,46	6,33	-8,17	-7,24	-7,705
DP047	610,71	0,08	3	38,69	3,99	6,52	7,01	5,5	5,94	5,99	-7,96	-7,13	-7,545
DP048	610,71	0,08	3	38,69	3,91	6,52	7,01	5,5	5,94	5,98	-7,96	-7,13	-7,545
DP049	610,71	0,08	3	38,69	4,11	6,52	7,01	5,5	5,94	6,01	-7,96	-7,13	-7,545
DP050	624,74	0,14	4	27,69	4,19	6,58	7,31	5,72	6,46	6,26	-8	-6,96	-7,48
DP051	545,84	0,14	4	27,69	3,9	6,16	6,55	5,12	5,77	5,67	-7,26	-6,52	-6,89
DP052	545,84	0,14	4	27,69	4,08	6,09	6,55	5,12	5,77	5,69	-7,22	-6,45	-6,835
DP053	703,64	0,14	4	27,69	4,48	7,27	8,07	6,32	7,16	6,90	-8,9	-7,68	-8,29
DP054	689,61	0,08	3	38,69	4,03	7,21	7,77	6,1	6,63	6,58	-8,86	-7,85	-8,355
DP055	466,95	0,14	4	27,69	3,76	5,3	5,78	4,51	5,09	5,04	-6,25	-5,63	-5,94
DP056	596,69	0	2	49,69	3,55	6,2	6,7	5,28	5,42	5,64	-7,76	-7,03	-7,395
DP057	517,79	0	2	49,69	3,31	5,51	5,94	4,66	4,74	5,02	-6,86	-6,31	-6,585

DP058	517,79	0	2	49,69	3,45	5,51	5,94	4,66	4,74	5,04	-6,86	-6,31	-6,585
DP059	438,89	0	2	49,69	3,15	4,81	5,18	4,04	4,06	4,40	-5,95	-5,59	-5,77
DP061	438,89	0	2	49,69	3,16	4,81	5,18	4,04	4,06	4,40	-5,95	-5,59	-5,77
DP062	580,69	0	2	29,46	3,75	6,55	7	5,89	5,89	6,01	-7,9	-6,97	-7,435
DP063	501,79	0	2	29,46	3,55	5,86	6,23	5,28	5,21	5,39	-7	-6,25	-6,625
DP064	501,79	0	2	29,46	3,58	5,86	6,23	5,28	5,21	5,40	-7	-6,25	-6,625
DP065	501,79	0	2	29,46	3,64	5,86	6,23	5,28	5,21	5,41	-7	-6,25	-6,625
DP066	515,82	0,08	3	18,46	3,66	6,19	6,54	5,51	5,72	5,69	-7,21	-6,36	-6,785
DP067	659,58	0	2	29,46	3,82	7,18	7,76	6,49	6,58	6,60	-8,76	-7,62	-8,19
DP068	501,79	0	2	29,46	3,63	5,79	6,23	5,28	5,21	5,40	-6,96	-6,18	-6,57
DP069	352,38	0,19	3	64,99	3,55	4,9	4,5	2,78	4,81	4,22	-5,26	-6	-5,63
DP070	400,42	0,27	8	113,29	3,24	4,8	4,32	2,59	4,14	3,93	-5,12	-6,91	-6,015
DP071	390,43	0,38	8	116,45	3,23	2,91	3,3	1,98	3,57	3,13	-3,88	-5,02	-4,45
DP072	404,45	0,41	9	105,45	3,26	3,45	3,95	2,2	4,13	3,57	-4,23	-5,35	-4,79
DP074	388,41	0,33	8	113,29	2,66	3,26	3,77	1,64	3,88	3,22	-4,09	-5,31	-4,7
DP075	659,58	0	2	29,46	3,82	7,18	7,76	6,49	6,58	6,60	-8,76	-7,62	-8,19
DP076	244,29	0,2	3	38,69	3	3,8	3,81	2,86	3,55	3,47	-4,04	-4,31	-4,175
DP077	246,26	0,14	2	69,92	2,05	3,11	3,21	2,02	2,54	2,69	-3,69	-4,25	-3,97
DP078	288,3	0,19	4	75,99	2,42	3,87	3,29	2,16	2,99	3,00	-4,22	-5,16	-4,69
DP079	288,3	0,19	4	75,99	2,33	3,32	3,29	2,43	2,99	2,94	-3,88	-4,59	-4,235
DP080	332,3	0,18	5	113,29	1,9	2,85	2,99	1,8	2,46	2,50	-3,74	-4,89	-4,315
DP081	288,3	0,19	4	75,99	2,42	3,87	3,29	2,16	2,99	3,00	-4,22	-5,16	-4,69
DP082	318,28	0,12	4	124,29	0,2	3,07	2,9	1,55	1,92	2,09	-3,87	-5,35	-4,61
DP083	274,27	0,13	3	86,99	2,06	3,55	3,21	2,18	2,46	2,78	-4,02	-5,06	-4,54

DP084	262,26	0,14	2	90,15	2,21	2,76	2,92	1,46	2,06	2,39	-3,54	-4,31	-3,925
DP085	262,26	0,14	2	90,15	2,26	2,76	2,92	1,46	2,06	2,40	-3,54	-4,31	-3,925
DP086	464,59	0,38	10	79,15	4,92	7,41	6,86	4,28	6,81	6,23	-7,12	-8,9	-8,01
DP087	1030,76	0	8	77,38	6,68	12,17	13,6	8,34	10,1	10,75	-13,89	-13,8	-13,845
DP088	1190,89	0	12	180,88	4,46	10,98	14,72	7,48	6,95	9,52	-13,78	-14,74	-14,26
DP089	1110,83	0	10	129,13	5,59	11,57	14,16	7,87	8,52	10,13	-13,83	-14,27	-14,05
DP090	1110,83	0	10	129,13	5,48	11,57	14,16	7,87	8,52	10,11	-13,83	-14,27	-14,05
DP092	368,72	0,12	5	133,52	1,05	3,31	3,26	1,54	2,13	2,42	-4,24	-5,79	-5,015
DP093	382,75	0,18	6	122,52	2,24	3,63	3,35	1,78	2,67	2,84	-4,45	-5,89	-5,17
DP094	396,78	0,22	7	111,52	2,73	3,96	3,43	2,01	3,22	3,13	-4,66	-6	-5,33
DP095	382,75	0,18	6	122,52	1,99	3,63	3,35	1,78	2,67	2,79	-4,45	-5,89	-5,17
DP096	348,3	0,18	6	122,52	1,91	3,01	2,69	1,28	2,02	2,27	-3,85	-5,25	-4,55
DP097	334,28	0,12	5	133,52	1,46	2,68	2,6	1,03	1,47	1,97	-3,64	-5,14	-4,39
DP098	473,3	0,33	10	111,52	3,58	5,84	5,26	3,17	5,1	4,70	-6,08	-7,95	-7,015
DP099	417,19	0,18	6	122,52	0,66	4,26	4	2,28	3,34	3,09	-5,04	-6,54	-5,79
DP100	431,22	0,22	7	111,52	2,57	4,59	4,09	2,51	3,88	3,62	-5,26	-6,66	-5,96
DP101	417,19	0,18	6	122,52	1,89	4,26	4	2,28	3,34	3,29	-5,04	-6,54	-5,79
XT018	300,26	0,12	2	96,97	2,28	3,02	2,67	1	2,9	2,42	-3,94	-4,72	-4,33
XT019	314,29	0,18	3	85,97	2,41	3,35	2,76	1,24	3,43	2,66	-4,15	-4,83	-4,49
XT021	232,23	0,23	2	67,51	1,81	1,18	1,94	0,44	3,13	1,74	-2,33	-2,19	-2,26
XT022	234,25	0,31	2	70,67	2,01	1,57	1,73	0,52	2,81	1,73	-2,58	-2,66	-2,62
XT023	384,38	0,38	2	87,36	3,52	3,82	3,42	1,03	3,76	3,16	-4,87	-5,35	-5,11
XT024	356,33	0,32	2	87,36	3,14	3,09	2,86	0,58	2,77	2,55	-4,26	-4,59	-4,425
XT025	234,25	0,46	0	66,76	2,05	1,91	1,42	0,73	1,77	1,55	-2,76	-2,94	-2,85

XT034	638,57	0,38	5	226,58	4,32	3,8	2,16	-1,36	1,85	2,16	-6,06	-8,25	-7,155
XT035	286,28	0,19	2	79,9	2,57	2,61	2,31	0,81	3,35	2,35	-3,62	-3,94	-3,78
XT036	358,3	0,17	5	123,27	3,15	2,9	2,06	0,36	2,77	2,24	-3,96	-5,15	-4,555
XT037	334,71	0,12	2	96,97	2,49	3,65	3,11	1,51	3,54	2,90	-4,53	-5,38	-4,955
XT038	334,71	0,12	2	96,97	2,54	3,65	3,11	1,51	3,54	2,91	-4,53	-5,38	-4,955
XT039	258,23	0,07	0	90,9	1,91	2,81	2,37	0,57	2,47	2,08	-3,76	-4,38	-4,07
XT040	258,23	0,07	0	90,9	2,04	2,81	2,37	0,57	2,47	2,11	-3,76	-4,38	-4,07
XT081	324,28	0,17	1	78,13	2,89	3,37	3,01	1,07	2,74	2,68	-4,34	-4,69	-4,515
XT170	340,37	0,25	4	79,9	3,32	4,11	3,65	1,67	4,58	3,52	-4,69	-5,49	-5,09
XT241	354,31	0,21	2	87,36	3,15	3,34	3,02	0,77	2,77	2,68	-4,41	-4,85	-4,63
XT886	424,49	0,4	3	96,22	3,89	5	4,03	2,08	4,76	4,02	-5,71	-6,76	-6,235

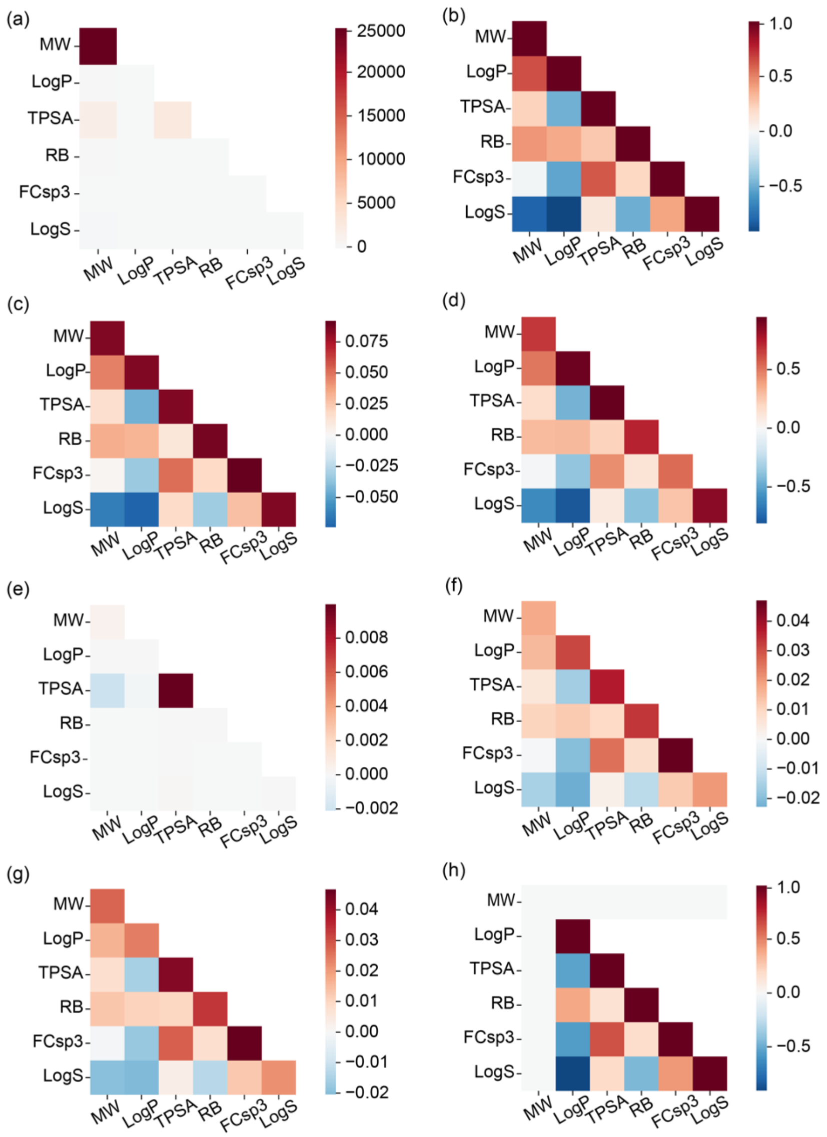

Figure S1. Heatmap of the distribution of the scaled dataset using different scaling techniques: a) without scaling; b) StandardScaler; (c) QuantileTransformer; (d) RobustScaler; (e) Normalizer; (f) MaxAbsScaler; (g) MinMaxScaler; (h) PowerTransformer.

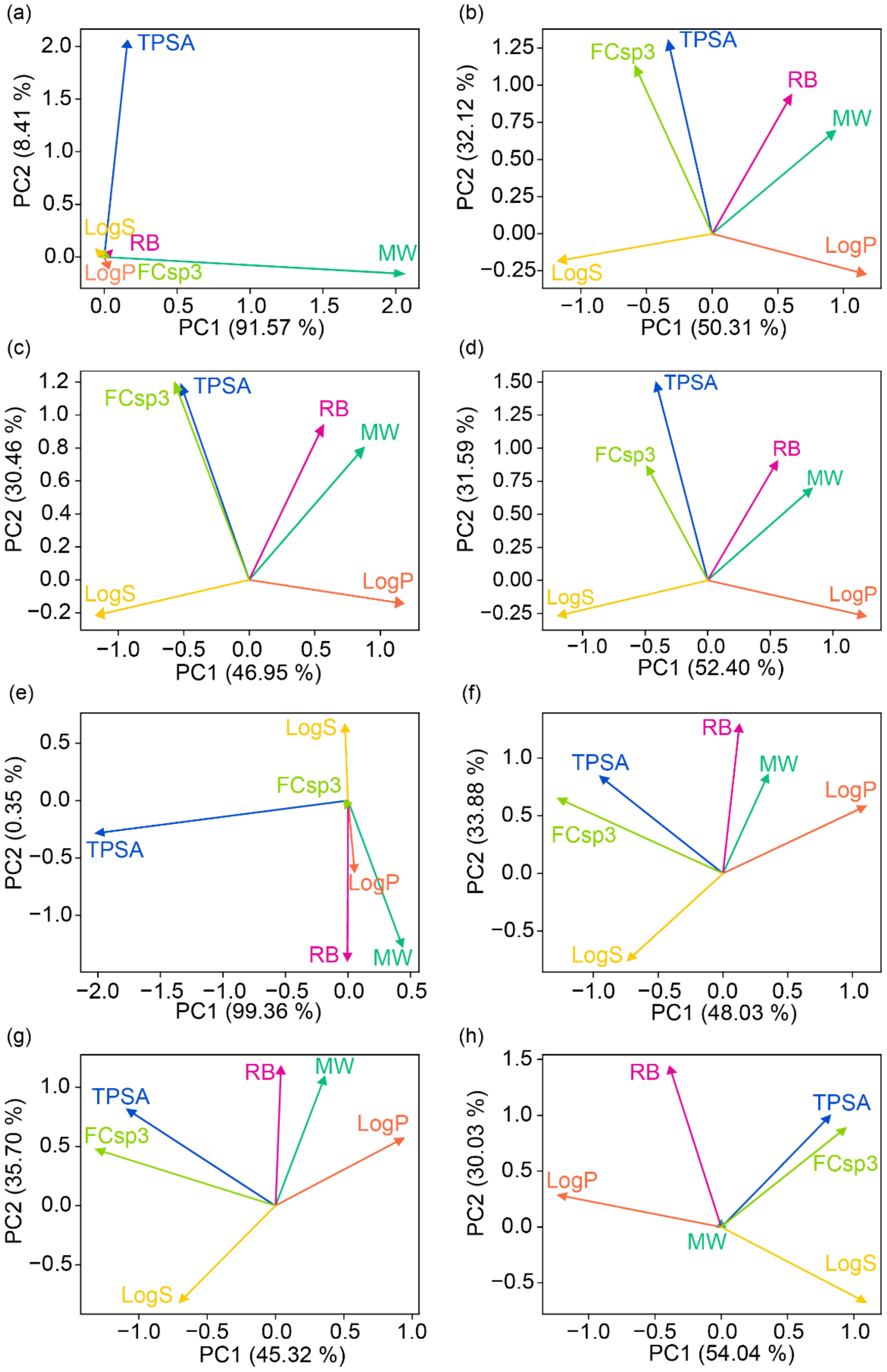

Figure S2. Loading plots obtained using different scaling techniques: a) without scaling; b) StandardScaler; (c) QuantileTransformer; (d)

RobustScaler; (e) Normalizer; (f) MaxAbsScaler; (g) MinMaxScaler; (h) PowerTransformer.

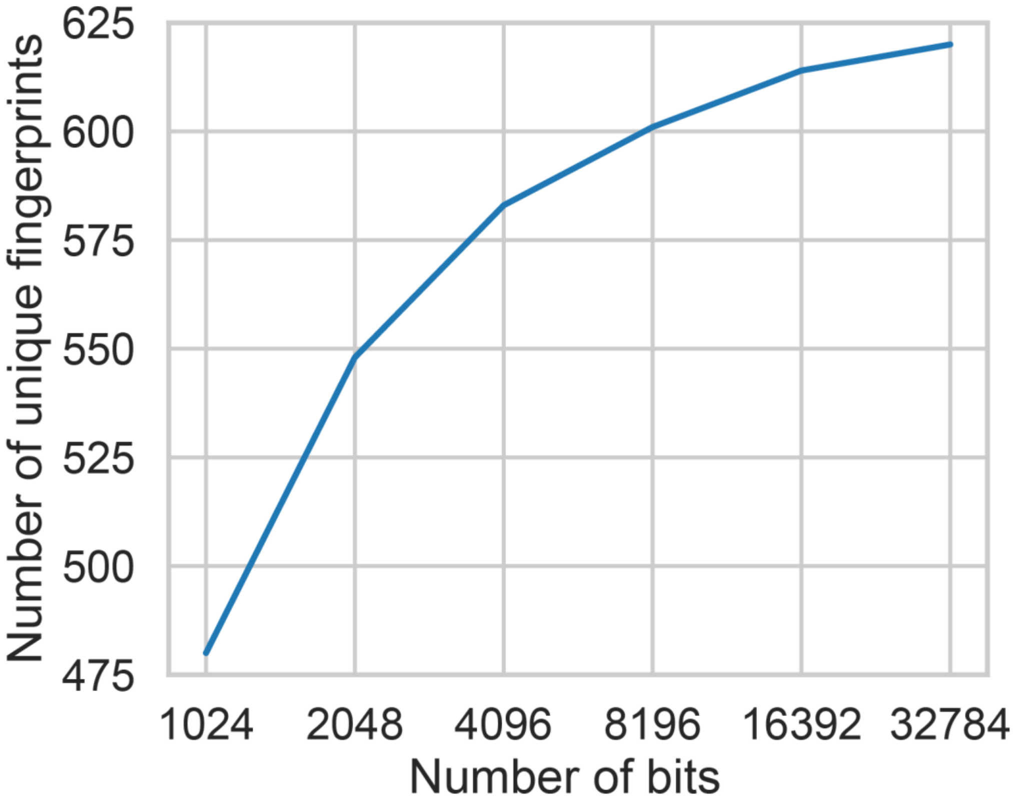

Figure S3. Number of unique fingerprints using different bits lengths.

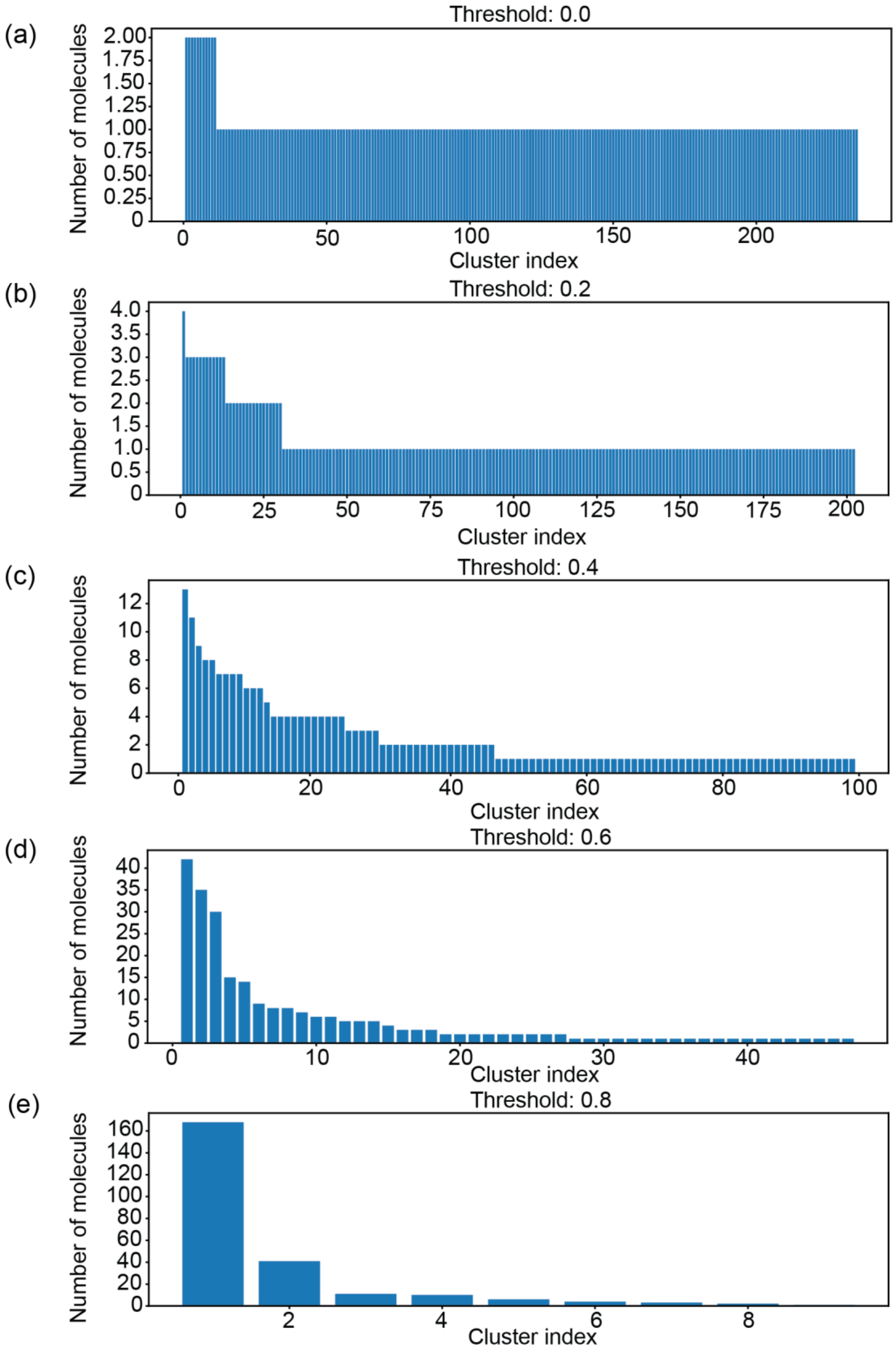

Figure S4. Number of clusters and the number of compounds per cluster using different threshold values.

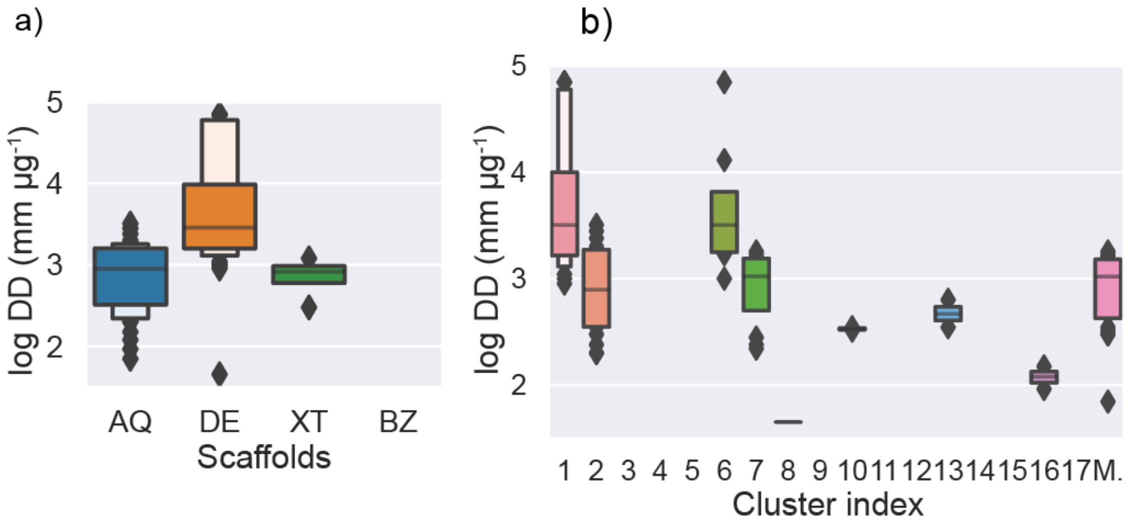

Figure S5. Distribution of log DD values according to the MP scaffolds (a) and clusters (b).

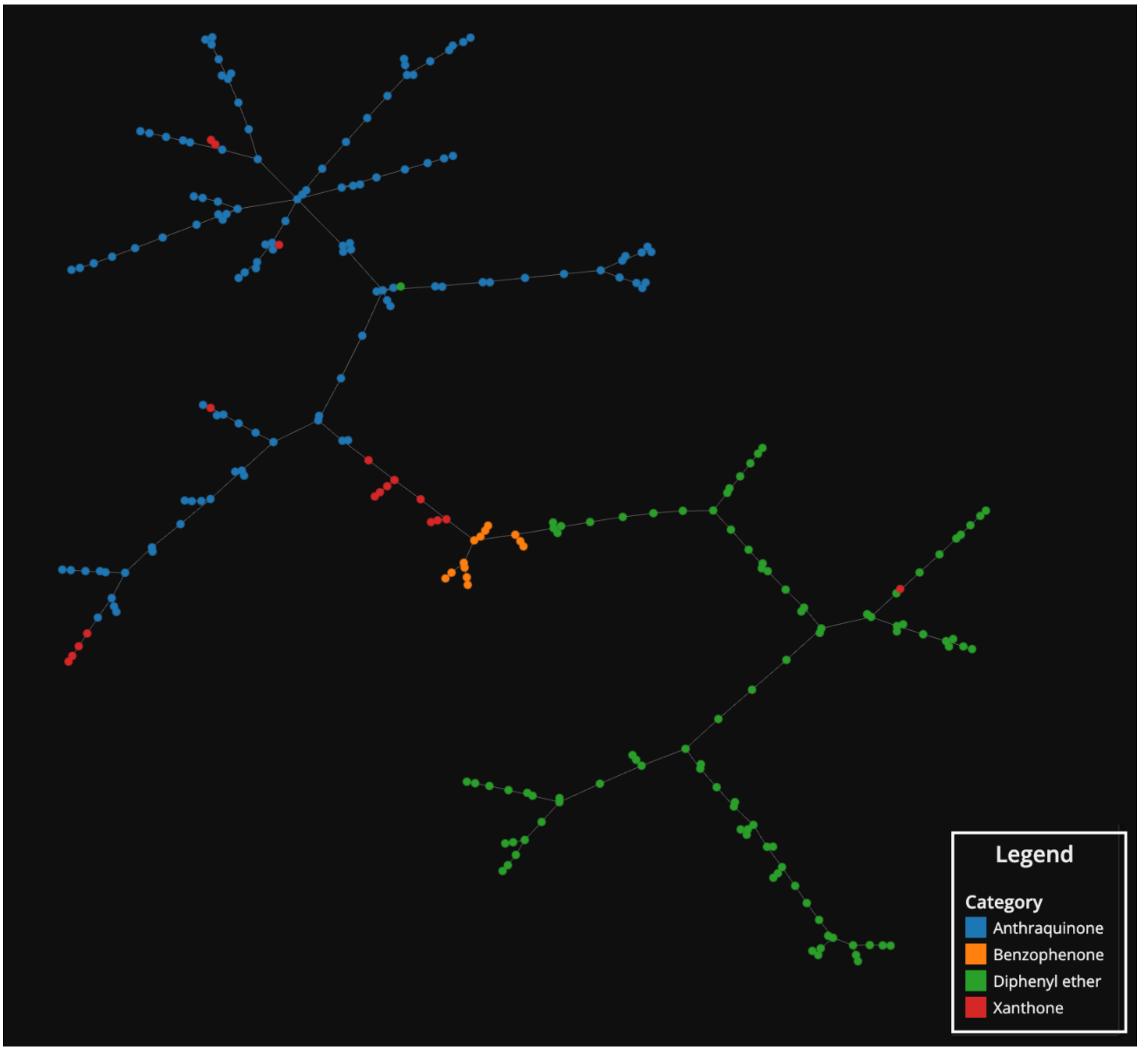

Figure S6. TMAP visualization of the studied MPs colored according to the scaffold.

References:

1. Li, J.L.; Jiang, X.; Liu, X.; He, C.; Di, Y.; Lu, S.; Huang, H.; Lin, B.; Wang, D.; Fan, B. Antibacterial Anthraquinone Dimers from Marine Derived Fungus Aspergillus Sp. *Fitoterapia* **2019**, *133*, 1–4, doi:10.1016/j.fitote.2018.11.015.
2. Zhang, S.-S.; Zhu, A.; Bai, X.; Zhu, H.-J.; Cao, F. Alkaloids and Polyketides from the Marine-Derived Fungus Aspergillus Versicolor. *Chem. Nat. Compd.* **2020**, *56*, 964–967, doi:10.1007/s10600-020-03203-y.
3. Poumale, H.M.P.; Ngadjui, B.T.; Helmke, E.; Laatscha, H. New Anthraquinones from a Marine Streptomyces Sp. – Isolation, Structure Determination and Biological Activities. **2006**, *61*, 1450–1454, doi:10.1515/znb-2006-1122.
4. Ge, X.; Sun, C.; Feng, Y.; Wang, L.; Peng, J.; Che, Q.; Gu, Q.; Zhu, T.; Li, D.; Zhang, G. Anthraquinone Derivatives from a Marine-Derived Fungus Sporendonema Casei HDN16-802. *Mar. Drugs* **2019**, *17*, doi:10.3390/md17060334.
5. Cui, H.X.; Shaaban, K.A.; Schiebel, M.; Qin, S.; Laatsch, H. New Antibiotic with Typical Plant Anthraquinone Structure Obtained Studying Terrestrial and Marine Streptomycetes. *World J. Microbiol. Biotechnol.* **2008**, *24*, 419–421, doi:10.1007/s11274-007-9477-5.
6. Abdissa, D.; Geleta, G.S.; Bacha, K.; Abdissa, N. Phytochemical Investigation of Aloe Pulcherrima Roots and Evaluation for Its Antibacterial and Antiplasmodial Activities. *PLoS ONE* **2017**, *12*, doi:10.1371/journal.pone.0173882.
7. Qi, J.; Zhao, P.; Zhao, L.; Jia, A.; Liu, C.; Zhang, L.; Xia, X. Anthraquinone Derivatives from a Sea Cucumber-Derived Trichoderma Sp. Fungus with Antibacterial Activities. *Chem. Nat. Compd.* **2020**, *56*, 112–114, doi:10.1007/s10600-020-02956-w.
8. ZHANG, Y.; LI, X.-M.; WANG, B.-G. Anthraquinone Derivatives Produced by Marine-Derived Fungus Aspergillus Versicolor EN-7. *Biosci. Biotechnol. Biochem.* **2012**, *76*, 1774–1776, doi:10.1271/bbb.120047.
9. El-Beih, A.A.; Kawabata, T.; Koimaru, K.; Ohta, T.; Tsukamoto, S. Monodictyquinone A: A New Antimicrobial Anthraquinone from a Sea Urchin-Derived Fungus Monodictys Sp. *Chem. Pharm. Bull. (Tokyo)* **2007**, *55*, 1097–1098, doi:10.1248/cpb.55.1097.
10. Du, F.-Y.; Li, X.-M.; Song, J.-Y.; Li, C.-S.; Wang, B.-G. Anthraquinone Derivatives and an Orsellinic Acid Ester from the Marine Alga-Derived Endophytic Fungus Eurotium Cristatum EN-220. *Helv. Chim. Acta* **2014**, *97*, 973–978, doi:10.1002/hlca.201300358.
11. Zhou, L.; Chen, X.; Sun, C.; Chang, Y.; Huang, X.; Zhu, T.; Zhang, G.; Che, Q.; Li, D. Saliniquinone Derivatives, Saliniquinones G−I and Heraclemycin E, from the Marine Animal-Derived Nocardiopsis Aegyptia HDN19-252. *Mar. Drugs* **2021**, *19*, doi:10.3390/md19100575.
12. Supong, K.; Thawai, C.; Suwanborirux, K.; Choowong, W.; Supothina, S.; Pittayakhajonwut, P. Antimalarial and Antitubercular C-Glycosylated Benz[α]Anthraquinones from the Marine-Derived Streptomyces Sp. BCC45596. *Phytochem. Lett.* **2012**, *5*, 651–656, doi:10.1016/j.phytol.2012.06.015.
13. de Sá, J.D.M.; Pereira, J.A.; Dethoup, T.; Cidade, H.; Sousa, M.E.; Rodrigues, I.C.; Costa, P.M.; Mistry, S.; Silva, A.M.S.; Kijjoa, A. Anthraquinones, Diphenyl Ethers, and Their Derivatives from the Culture of the Marine Sponge-Associated Fungus Neosartorya Spinosa KUFA 1047. *Mar. Drugs* **2021**, *19*, doi:10.3390/md19080457.

14. May Zin, W.W.; Buttachon, S.; Dethoup, T.; Pereira, J.A.; Gales, L.; Inácio, Â.; Costa, P.M.; Lee, M.; Sekeroglu, N.; Silva, A.M.S.; et al. Antibacterial and Antibiofilm Activities of the Metabolites Isolated from the Culture of the Mangrove-Derived Endophytic Fungus Eurotium Chevalieri KUFA 0006. *Phytochemistry* **2017**, *141*, 86–97, doi:10.1016/j.phytochem.2017.05.015.
15. Khamthong, N.; Rukachaisirikul, V.; Tadpetch, K.; Kaewpet, M.; Phongpaichit, S.; Preedanon, S.; Sakayaroj, J. Tetrahydroanthraquinone and Xanthone Derivatives from the Marine-Derived Fungus Trichoderma Aureoviride PSU-F95. *Arch. Pharm. Res.* **2012**, *35*, 461–468, doi:10.1007/s12272-012-0309-2.
16. Wang, W.; Chen, R.; Luo, Z.; Wang, W.; Chen, J. Antimicrobial Activity and Molecular Docking Studies of a Novel Anthraquinone from a Marine-Derived Fungus Aspergillus Versicolor. *Nat. Prod. Res.* **2018**, *32*, 558–563, doi:10.1080/14786419.2017.1329732.
17. Lee, Y.M.; Li, H.; Hong, J.; Cho, H.Y.; Bae, K.S.; Kim, M.A.; Kim, D.-K.; Jung, J.H. Bioactive Metabolites from the Sponge-Derived Fungus Aspergillus Versicolor. *Arch. Pharm. Res.* **2010**, *33*, 231–235, doi:10.1007/s12272-010-0207-4.
18. Xu, J.; Nakazawa, T.; Ukai, K.; Kobayashi, H.; Mangindaan, R.E.P.; Wewengkang, D.S.; Rotinsulu, H.; Namikoshi, M. Tetrahydrobostrycin and 1-Deoxytetrahydrobostrycin, Two New Hexahydroanthrone Derivatives, from a Marine-Derived Fungus Aspergillus Sp. *J. Antibiot. (Tokyo)* **2008**, *61*, 415–419, doi:10.1038/ja.2008.57.
19. Sibero, M.T.; Zhou, T.; Fukaya, K.; Urabe, D.; Radjasa, O.K.K.; Sabdono, A.; Trianto, A.; Igarashi, Y. Two New Aromatic Polyketides from a Sponge-Derived Fusarium. *Beilstein J. Org. Chem.* **2019**, *15*, 2941–2947, doi:10.3762/bjoc.15.289.
20. Shaaban, K.A.; Shaaban, M.; Meiners, M.; Schüffler, A.; Kelter, G.; Fiebig, H.-H.; Laatsch, H. Boshramycinones A-C: New Anthracyclinones Produced by a Marine-Derived Streptomyces Sp.: Isolation, Structure Elucidation and Biological Activities. *Nat. Prod. Res.* **2021**, *35*, 1281–1291, doi:10.1080/14786419.2019.1645658.
21. Abdelfattah, M.S. Mansoquinone: Isolation and Structure Elucidation of New Antibacterial Aromatic Polyketides from Terrestrial Streptomyces Sp. Eg5. *Nat. Prod. Res.* **2009**, *23*, 212–218, doi:10.1080/14786410801961550.
22. Hawas, U.W.; El-Beih, A.A.; El-Halawany, A.M. Bioactive Anthraquinones from Endophytic Fungus Aspergillus Versicolor Isolated from Red Sea Algae. *Arch. Pharm. Res.* **2012**, *35*, 1749–1756, doi:10.1007/s12272-012-1006-x.
23. Kim, M.C.; Cullum, R.; Hebishy, A.M.S.; Mohamed, H.A.; Faraag, A.H.I.; Salah, N.M.; Abdelfattah, M.S.; Fenical, W. Mersaquinone, A New Tetracene Derivative from the Marine-Derived Streptomyces Sp. EG1 Exhibiting Activity against Methicillin-Resistant Staphylococcus Aureus (MRSA). *Antibiotics* **2020**, *9*, doi:10.3390/antibiotics9050252.
24. Adinarayana, G.; Venkateshan, M.R.; Bapiraju, V.V.S.N.K.; Sujatha, P.; Premkumar, J.; Ellaiah, P.; Zeeck, A. Cytotoxic Compounds from the Marine Actinobacterium Streptomyces Corchorusii AUBN1/71. *Russ. J. Bioorganic Chem.* **2006**, *32*, 295–300, doi:10.1134/S1068162006030125.
25. Yan, H.-J.; Li, X.-M.; Li, C.-S.; Wang, B.-G. Alkaloid and Anthraquinone Derivatives Produced by the Marine-Derived Endophytic Fungus Eurotium Rubrum. *Helv. Chim. Acta* **2012**, *95*, 163–168, doi:10.1002/hlca.201100255.
26. Wang, J.; He, W.; Huang, X.; Tian, X.; Liao, S.; Yang, B.; Wang, F.; Zhou, X.; Liu, Y. Antifungal New Oxepine-Containing Alkaloids and Xanthones from the Deep-

Sea-Derived Fungus Aspergillus Versicolor SCSIO 05879. *J. Agric. Food Chem.* **2016**, *64*, 2910–2916, doi:10.1021/acs.jafc.6b00527.
27. Yang, K.-L.; Wei, M.-Y.; Shao, C.-L.; Fu, X.-M.; Guo, Z.-Y.; Xu, R.-F.; Zheng, C.-J.; She, Z.-G.; Lin, Y.-C.; Wang, C.-Y. Antibacterial Anthraquinone Derivatives from a Sea Anemone-Derived Fungus Nigrospora Sp. *J. Nat. Prod.* **2012**, *75*, 935–941, doi:10.1021/np300103w.
28. Hu, J.; Li, Z.; Gao, J.; He, H.; Dai, H.; Xia, X.; Liu, C.; Zhang, L.; Song, F. New Diketopiperazines from a Marine-Derived Fungus Strain Aspergillus Versicolor MF180151. *Mar. Drugs* **2019**, *17*, doi:10.3390/md17050262.
29. Tian, Y.-Q.; Lin, S.-T.; Kumaravel, K.; Zhou, H.; Wang, S.-Y.; Liu, Y.-H. Polyketide-Derived Metabolites from the Sponge-Derived Fungus Aspergillus Sp. F40. *Phytochem. Lett.* **2018**, *27*, 74–77, doi:10.1016/j.phytol.2018.06.009.
30. Jadulco, R.; Brauers, G.; Edrada, R.A.; Ebel, R.; Wray, V.; Sudarsono; Proksch, P. New Metabolites from Sponge-Derived Fungi Curvularia Lunata and Cladosporium Herbarum. *J. Nat. Prod.* **2002**, *65*, 730–733, doi:10.1021/np010390i.
31. Dong, Y.; Ding, W.; Sun, C.; Ji, X.; Ling, C.; Zhou, Z.; Chen, Z.; Chen, X.; Ju, J. Julichrome Monomers from Marine Gastropod Mollusk-Associated Streptomyces and Stereochemical Revision of Julichromes Q3 · 5 and Q3 · 3. *Chem. Biodivers.* **2020**, *17*, e2000057, doi:10.1002/cbdv.202000057.
32. Abdel-Wahab, N.M.; Scharf, S.; Özkaya, F.C.; Kurtán, T.; Mándi, A.; Fouad, M.A.; Kamel, M.S.; Müller, W.E.G.; Kalscheuer, R.; Lin, W.; et al. Induction of Secondary Metabolites from the Marine-Derived Fungus Aspergillus Versicolor through Co-Cultivation with Bacillus Subtilis. *Planta Med.* **2019**, *85 6*, 503–512.
33. Li, S.; Tian, X.; Niu, S.; Zhang, W.; Chen, Y.; Zhang, H.; Yang, X.; Zhang, W.; Li, W.; Zhang, S.; et al. Pseudonocardians A–C, New Diazaanthraquinone Derivatives from a Deap-Sea Actinomycete Pseudonocardia Sp. SCSIO 01299. *Mar. Drugs* **2011**, *9*, 1428–1439, doi:10.3390/md9081428.
34. El-Gendy, M.M.A.; Hawas, U.W.; Jaspars, M. Novel Bioactive Metabolites from a Marine Derived Bacterium Nocardia Sp. ALAA 2000. *J. Antibiot. (Tokyo)* **2008**, *61*, 379–386, doi:10.1038/ja.2008.53.
35. Socha, A.M.; LaPlante, K.L.; Rowley, D.C. New Bisanthraquinone Antibiotics and Semi-Synthetic Derivatives with Potent Activity against Clinical Staphylococcus Aureus and Enterococcus Faecium Isolates. *Bioorg. Med. Chem.* **2006**, *14*, 8446–8454, doi:10.1016/j.bmc.2006.08.038.
36. Lai, Z.; Yu, J.; Ling, H.; Song, Y.; Yuan, J.; Ju, J.; Tao, Y.; Huang, H. Grincamycins I−K, Cytotoxic Angucycline Glycosides Derived from Marine-Derived Actinomycete Streptomyces Lusitanus SCSIO LR32. *Planta Med* **2018**, *84*, 201–207.
37. Zhou, B.; Ji, Y.-Y.; Zhang, H.-J.; Shen, L. Gephyyamycin and Cysrabelomycin, Two New Angucyclinone Derivatives from the Streptomyces Sp. HN-A124. *Nat. Prod. Res.* **2021**, *35*, 2117–2122, doi:10.1080/14786419.2019.1660336.
38. Wang, S.; Li, X.-M.; Teuscher, F.; Li; Diesel, A.; Ebel, R.; Proksch, P.; Wang, B.-G. Chaetopyranin, a Benzaldehyde Derivative, and Other Related Metabolites from Chaetomium Globosum, an Endophytic Fungus Derived from the Marine Red Alga Polysiphonia Urceolata. *J. Nat. Prod.* **2006**, *69*, 1622–1625, doi:10.1021/np060248n.
39. Manojlovic, N.; Solujic, S.R.; Sukdolak, S.; Krstić, L. Isolation and Antimicrobial Activity of Anthraquinones from Some Species of the Lichen Genus Xanthoria. *J. Serbian Chem. Soc.* **2000**, *65*, 555–560.

40. Basile, A.; Rigano, D.; Loppi, S.; Di Santi, A.; Nebbioso, A.; Sorbo, S.; Conte, B.; Paoli, L.; De Ruberto, F.; Molinari, A.M.; et al. Antiproliferative, Antibacterial and Antifungal Activity of the Lichen Xanthoria Parietina and Its Secondary Metabolite Parietin. *Int. J. Mol. Sci.* **2015**, *16*, 7861–7875, doi:10.3390/ijms16047861.
41. Song, X.; Tu, R.; Mei, X.; Wu, S.; Lan, B.; Zhang, L.; Luo, X.; Liu, J.; Luo, M. A Mycophenolic Acid Derivative from the Fungus Penicillium Sp. SCSIO Sof101. *Nat. Prod. Res.* **2020**, *34*, 1206–1212, doi:10.1080/14786419.2018.1553881.
42. Luo, H.; Li, X.-M.; Li, C.-S.; Wang, B.-G. Diphenyl Ether and Benzophenone Derivatives from the Marine Mangrove-Derived Fungus Penicillium Sp. MA-37. *Phytochem. Lett.* **2014**, *9*, 22–25, doi:10.1016/j.phytol.2014.03.012.
43. Lei, H.; Lin, X.; Han, L.; Ma, J.; Ma, Q.; Zhong, J.; Liu, Y.; Sun, T.; Wang, J.; Huang, X. New Metabolites and Bioactive Chlorinated Benzophenone Derivatives Produced by a Marine-Derived Fungus Pestalotiopsis Heterocornis. *Mar. Drugs* **2017**, *15*, doi:10.3390/md15030069.
44. Ji, Y.-B.; Chen, W.-J.; Shan, T.-Z.; Sun, B.-Y.; Yan, P.-C.; Jiang, W. Antibacterial Diphenyl Ether, Benzophenone and Xanthone Derivatives from Aspergillus Flavipes. *Chem. Biodivers.* **2020**, *17*, e1900640, doi:10.1002/cbdv.201900640.
45. Li, H.-L.; Li, X.-M.; Liu, H.; Meng, L.-H.; Wang, B.-G. Two New Diphenylketones and a New Xanthone from Talaromyces Islandicus EN-501, an Endophytic Fungus Derived from the Marine Red Alga Laurencia Okamurai. *Mar. Drugs* **2016**, *14*, doi:10.3390/md14120223.
46. Bai, M.; Gao, C.-H.; Liu, K.; Zhao, L.-Y.; Tang, Z.-Z.; Liu, Y.-H. Two New Benzophenones Isolated from a Mangrove-Derived Fungus Penicillium Sp. *J. Antibiot. (Tokyo)* **2021**, *74*, 821–824, doi:10.1038/s41429-021-00464-9.
47. Cueto, M.; Jensen, P.R.; Kauffman, C.; Fenical, W.; Lobkovsky, E.; Clardy, J. Pestalone, a New Antibiotic Produced by a Marine Fungus in Response to Bacterial Challenge. *J. Nat. Prod.* **2001**, *64*, 1444–1446, doi:10.1021/np0102713.
48. Zheng, C.-J.; Liao, H.-X.; Mei, R.-Q.; Huang, G.-L.; Yang, L.-J.; Zhou, X.-M.; Shao, T.-M.; Chen, G.-Y.; Wang, C.-Y. Two New Benzophenones and One New Natural Amide Alkaloid Isolated from a Mangrove-Derived Fungus Penicillium Citrinum. *Nat. Prod. Res.* **2019**, *33*, 1127–1134, doi:10.1080/14786419.2018.1460832.
49. Buttachon, S.; May Zin, W.W.; Dethoup, T.; Gales, L.; Pereira, J.A.; Silva, A.M.S.; Kijjoa, A. Secondary Metabolites from the Culture of the Marine Sponge-Associated Fungi Talaromyces Tratensis and Sporidesmium Circinophorum. *Planta Med* **2016**, *82*, 888–896.
50. Figueroa, L.; Jiménez, C.; Rodríguez, J.; Areche, C.; Chávez, R.; Henríquez, M.; de la Cruz, M.; Díaz, C.; Segade, Y.; Vaca, I. 3-Nitroasterric Acid Derivatives from an Antarctic Sponge-Derived Pseudogymnoascus Sp. Fungus. *J. Nat. Prod.* **2015**, *78*, 919–923, doi:10.1021/np500906k.
51. Radwan, M.M.; Wanas, A.S.; Fronczek, F.R.; Jacob, M.R.; Ross, S.A. Polybrominated Diphenyl Ethers from the Marine Organisms Lendenfeldia Dendyi and Sinularia Dura with Anti-MRSa Activity. *Med. Chem. Res.* **2015**, *24*, 3398–3404, doi:10.1007/s00044-015-1386-9.
52. Ki, D.-W.; Awouafack, M.D.; Wong, C.P.; Nguyen, H.M.; Thai, Q.M.; Ton Nu, L.H.; Morita, H. Brominated Diphenyl Ethers Including a New Tribromoiododiphenyl Ether from the Vietnamese Marine Sponge Arenosclera Sp. and Their Antibacterial Activities. *Chem. Biodivers.* **2019**, *16*, e1800593, doi:10.1002/cbdv.201800593.

53. Zhang, H.; Skildum, A.; Stromquist, E.; Rose-Hellekant, T.; Chang, L.C. Bioactive Polybrominated Diphenyl Ethers from the Marine Sponge Dysidea Sp. *J. Nat. Prod.* **2008**, *71*, 262–264, doi:10.1021/np070244y.
54. Li, Z.-X.; Wang, X.-F.; Ren, G.-W.; Yuan, X.-L.; Deng, N.; Ji, G.-X.; Li, W.; Zhang, P. Prenylated Diphenyl Ethers from the Marine Algal-Derived Endophytic Fungus Aspergillus Tennesseensis. *Molecules* **2018**, *23*, doi:10.3390/molecules23092368.
55. Xu, X.; Yang, H.; Xu, H.; Yin, L.; Chen, Z.; Shen, H. Diphenyl Ethers from a Marine-Derived Isolate of Aspergillus Sp. CUGB-F046. *Nat. Prod. Res.* **2018**, *32*, 821–825, doi:10.1080/14786419.2017.1363754.
56. Hanif, N.; Tanaka, J.; Setiawan, A.; Trianto, A.; de Voogd, N.J.; Murni, A.; Tanaka, C.; Higa, T. Polybrominated Diphenyl Ethers from the Indonesian Sponge Lamellodysidea Herbacea. *J. Nat. Prod.* **2007**, *70*, 432–435, doi:10.1021/np0605081.
57. Sun, S.; Canning, C.B.; Bhargava, K.; Sun, X.; Zhu, W.; Zhou, N.; Zhang, Y.; Zhou, K. Polybrominated Diphenyl Ethers with Potent and Broad Spectrum Antimicrobial Activity from the Marine Sponge Dysidea. *Bioorg. Med. Chem. Lett.* **2015**, *25*, 2181–2183, doi:10.1016/j.bmcl.2015.03.057.
58. Handayani, D.; Edrada, R.A.; Proksch, P.; Wray, V.; Witte, L.; Van Soest, R.W.M.; Kunzmann, A.; Soedarsono Four New Bioactive Polybrominated Diphenyl Ethers of the Sponge Dysidea Herbacea from West Sumatra, Indonesia. *J. Nat. Prod.* **1997**, *60*, 1313–1316, doi:10.1021/np970271w.
59. Zhang, Y.; Li, X.-M.; Shang, Z.; Li, C.-S.; Ji, N.-Y.; Wang, B.-G. Meroterpenoid and Diphenyl Ether Derivatives from Penicillium Sp. MA-37, a Fungus Isolated from Marine Mangrove Rhizospheric Soil. *J. Nat. Prod.* **2012**, *75*, 1888–1895, doi:10.1021/np300377b.
60. Oh, H.; Kwon, T.O.; Gloer, J.B.; Marvanová, L.; Shearer, C.A. Tenellic Acids A−D: New Bioactive Diphenyl Ether Derivatives from the Aquatic Fungus Dendrospora Tenella. *J. Nat. Prod.* **1999**, *62*, 580–583, doi:10.1021/np980496m.
61. Faisal, M.R.; Kellermann, M.Y.; Rohde, S.; Putra, M.Y.; Murniasih, T.; Risdian, C.; Mohr, K.I.; Wink, J.; Praditya, D.F.; Steinmann, E.; et al. Ecological and Pharmacological Activities of Polybrominated Diphenyl Ethers (PBDEs) from the Indonesian Marine Sponge Lamellodysidea Herbacea. *Mar. Drugs* **2021**, *19*, doi:10.3390/md19110611.
62. Liu, S.; Dai, H.; Konuklugil, B.; Orfali, R.S.; Lin, W.; Kalscheuer, R.; Liu, Z.; Proksch, P. Phenolic Bisabolanes from the Sponge-Derived Fungus Aspergillus Sp. *Phytochem. Lett.* **2016**, *18*, 187–191, doi:10.1016/j.phytol.2016.10.015.
63. Ningsih, B.N.S.; Rukachaisirikul, V.; Pansrinun, S.; Phongpaichit, S.; Preedanon, S.; Sakayaroj, J. New Aromatic Polyketides from the Marine-Derived Fungus Pseudopithomyces Maydicus PSU-AMF350 and Their Antimicrobial Activity. *Nat. Prod. Res.* **2021**, 1–8, doi:10.1080/14786419.2021.1915309.
64. Liu, W.; Wang, L.; Wang, B.; Xu, Y.; Zhu, G.; Lan, M.; Zhu, W.; Sun, K. Diketopiperazine and Diphenylether Derivatives from Marine Algae-Derived Aspergillus Versicolor OUCMDZ-2738 by Epigenetic Activation. *Mar. Drugs* **2019**, *17*, doi:10.3390/md17010006.
65. Liu, S.; Wang, H.; Su, M.; Hwang, G.J.; Hong, J.; Jung, J.H. New Metabolites from the Sponge-Derived Fungus Aspergillus Sydowii J05B-7F-4. *Nat. Prod. Res.* **2017**, *31*, 1682–1686, doi:10.1080/14786419.2017.1289205.
66. Li, X.-D.; Li, X.-M.; Xu, G.-M.; Zhang, P.; Wang, B.-G. Antimicrobial Phenolic Bisabolanes and Related Derivatives from Penicillium Aculeatum SD-321, a Deep

Sea Sediment-Derived Fungus. *J. Nat. Prod.* **2015**, *78*, 844–849, doi:10.1021/acs.jnatprod.5b00004.
67. Choi, H.; Engene, N.; Smith, J.E.; Preskitt, L.B.; Gerwick, W.H. Crossbyanols A−D, Toxic Brominated Polyphenyl Ethers from the Hawai'ian Bloom-Forming Cyanobacterium Leptolyngbya Crossbyana. *J. Nat. Prod.* **2010**, *73*, 517–522, doi:10.1021/np900661g.
68. Sun, R.-R.; Miao, F.-P.; Zhang, J.; Wang, G.; Yin, X.-L.; Ji, N.-Y. Three New Xanthone Derivatives from an Algicolous Isolate of Aspergillus Wentii. *Magn. Reson. Chem.* **2013**, *51*, 65–68, doi:10.1002/mrc.3903.
69. Cai, S.; Zhu, T.; Du, L.; Zhao, B.; Li, D.; Gu, Q. Sterigmatocystins from the Deep-Sea-Derived Fungus Aspergillus Versicolor. *J. Antibiot. (Tokyo)* **2011**, *64*, 193–196, doi:10.1038/ja.2010.154.
70. Liu, Y.; Ding, L.; He, J.; Zhang, Z.; Deng, Y.; He, S.; Yan, X. A New Antibacterial Chromone from a Marine Sponge-Associated Fungus Aspergillus Sp. LS57. *Fitoterapia* **2021**, *154*, 105004, doi:10.1016/j.fitote.2021.105004.
71. Wu, B.; Wiese, J.; Wenzel-Storjohann, A.; Malien, S.; Schmaljohann, R.; Imhoff, J.F. Engyodontochones, Antibiotic Polyketides from the Marine Fungus Engyodontium Album Strain LF069. *Chem. – Eur. J.* **2016**, *22*, 7452–7462, doi:10.1002/chem.201600430.
72. Bao, J.; Sun, Y.-L.; Zhang, X.-Y.; Han, Z.; Gao, H.-C.; He, F.; Qian, P.-Y.; Qi, S.-H. Antifouling and Antibacterial Polyketides from Marine Gorgonian Coral-Associated Fungus Penicillium Sp. SCSGAF 0023. *J. Antibiot. (Tokyo)* **2013**, *66*, 219–223, doi:10.1038/ja.2012.110.
